# Predicting the Effects of Climate Change on the Fertility of Aquatic Animals Using a Meta‐Analytic Approach

**DOI:** 10.1111/ele.70054

**Published:** 2024-12-31

**Authors:** Amber Chatten, Isobel Grieve, Eirini Meligoniti, Claudia Hayward, Natalie Pilakouta

**Affiliations:** ^1^ Centre for Biological Diversity, School of Biology University of St Andrews St Andrews UK; ^2^ School of Biological Sciences University of Aberdeen Aberdeen UK

**Keywords:** fecundity, fertilisation, gamete, gonad, meta‐analysis, reproduction, temperature

## Abstract

Given that reproductive physiology is highly sensitive to thermal stress, there is increasing concern about the effects of climate change on animal fertility. Even a slight reduction in fertility can have consequences for population growth and survival, so it is critical to better understand and predict the potential effects of climate change on reproductive traits. We synthesised 1894 effect sizes across 276 studies on 241 species to examine thermal effects on fertility in aquatic animals. Our meta‐analysis revealed that external fertilisers tend to be more vulnerable to warming than internal fertilisers, especially in freshwater species. We also found that increased temperature is particularly detrimental for gametes and that under certain conditions, female fertility is more sensitive to warming than male fertility, challenging the prevailing view that males are more vulnerable. This work provides valuable new insights into the effects of temperature on fertility, with potential consequences for population viability.

## Graphical abstract

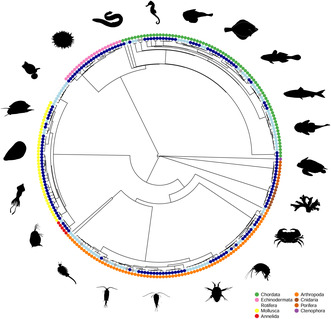

## 1 Introduction

Reproductive physiology is very sensitive to thermal stress (Yao et al. 2000; Wudarski et al. 2019; Servili et al. 2020; Han et al. 2020; Stubbs et al. 2020). There is thus increasing concern about the effects of rising temperatures on animal fertility in light of global climate change (Pankhurst et al. 2011; Walsh et al. 2019; Wild et al. 2019; van Heerwaarden and Sgrò 2021). Elevated temperatures can affect a range of reproductive traits, from gonad development to gamete quantity and quality to fertilisation success and offspring performance (Chen and Chen 1992; Barbraud and Weimerskirch 2001; Lin et al. 2006; Pörtner and Peck 2010; Pankhurst et al. 2011; Baumann, Talmage, and Gobler 2012; Rosa et al. 2014; Ciannelli, Bailey, and Olsen 2015; Pilakouta et al. 2023). In fact, recent work suggests that thermal fertility limits are much better predictors of extinction than upper critical thermal limits, which represent lethal temperatures (van Heerwaarden and Sgrò 2021). Ectothermic and externally fertilising species are predicted to be particularly vulnerable to warming, although fertility is also highly sensitive to heat stress in internal fertilisers, including humans (Walsh et al. 2019). Given that even a slight reduction in fertility can have consequences for population survival, there is a need to better understand and predict the effects of climate change on reproductive traits (Walsh et al. 2019; Dahlke et al. 2020).

Here, we use a meta‐analytic approach to examine the effects of temperature on fertility in freshwater and marine animals. Synthesising the findings of published studies allows us to reveal general patterns across taxa and can help us identify traits or attributes that influence a species' vulnerability to warming (Spector and Thompson 1991; Borenstein et al. 2021; Pilakouta and Baillet 2022). This approach therefore offers a powerful tool for improving our ability to make informed predictions about the consequences of climate change for animal fertility. This knowledge could be pivotal in informing conservation efforts and strategies to safeguard vulnerable species in a warming world (Pratchett et al. 2011).

In addition to testing for an overall effect of temperature on fertility across all species in our dataset, we tested the effects of several moderators (Table S1). We first examined whether the effects of temperature on fertility depend on a range of species characteristics relating to physiology and life history. For example, we expected that increased temperature would have a stronger negative effect on external fertilisers than internal fertilisers because gametes of these species are directly exposed to the environment and thus the ambient temperature (Walsh et al. 2019). We also predicted a larger reduction in fertility when the experimental temperature that animals were exposed to was closer to their critical thermal limit (CT_max_). Similarly, tropical species are predicted to be more vulnerable to elevated temperatures because they are more likely to be closer to their upper thermal limits than temperate or polar species (Deutsch et al. 2008; Nati et al. 2021). In addition, given that marine species typically experience more stable temperatures than freshwater species, they may be more negatively affected by increased temperature due to a slower rate of acclimation and lower level of phenotypic plasticity (Einum and Burton 2023).

We then tested whether differences in the effects of temperature on fertility could be explained by the type of fertility trait being examined. For example, we predicted that male fertility would be more negatively affected by increased temperature than female fertility, given accumulating evidence that sperm is highly sensitive to thermal stress (Mcdaniel et al. 1995; van Heerwaarden and Sgrò 2021; Wang and Gunderson 2022; Santos et al. 2023). We also expected that gametes would be the most vulnerable developmental stage, due to their lower thermal tolerance and lack of homeostatic systems (Andronikov 1975; Jonsson and Jonsson 2009; Elliott and Elliott 2010; Adriaenssens et al. 2012; Jatteau et al. 2017; Foo and Byrne 2017; Dahlke et al. 2020).

Lastly, we examined whether the effects of temperature on fertility vary based on a study's experimental design, focusing on the duration and magnitude of the temperature change, whether temperatures were constant or fluctuating and whether temperature variation was natural or due to experimental manipulation. We expected that larger increases in temperature and those that were longer in duration would have more harmful effects, particularly as temperatures approach the organisms' thermal limits (Walsh et al. 2019). By using a meta‐analytic approach, this work can provide novel insights into the effects of climate change on fertility in aquatic animals and offer enhanced predictive ability for how certain species traits may influence vulnerability to warming.

## 2 Material and Methods

### 2.1 Literature Search and Data Collection

This study was not pre‐registered. To identify relevant studies for our meta‐analysis, we used a large systematic map (Dougherty et al. 2024), which was created by searching published literature using the ISI Web of Science Core and following the ROSES Reporting Standards for Systematic Evidence Syntheses guidelines and the PRISMA guidelines (Haddaway et al. 2018; O'Dea et al. 2021; Table S8). Studies included in this systematic map were peer‐reviewed journal articles or book chapters that measured one or more reproductive traits at two or more temperatures in non‐human animals. The systematic map includes 427 papers that contain data on the effects of temperature on fertility in aquatic animals. We excluded 151 of these from our meta‐analysis for a variety of reasons (Figure S1; Table S9). For example, we excluded studies with data on gonad developmental stages and gonad gene expression, which have no clear link to fertility. We included gonad traits such as gonadosomatic index and gonad size, which have a clearer relationship with reproductive success. We also excluded studies examining seasonal temperature variations outside the breeding season, which was not relevant to our question. For studies with insufficient information for calculating effect sizes, we contacted the authors to request the missing data. Out of 37 authors, four responded, with one providing the necessary data. We thus had to exclude the remaining 36 studies (Figure S1).

Our final analysis included 276 papers, and each paper was assigned to one of the authors (AC, IG, EM or CH). The data were then compiled, cross‐checked and validated by the first (AC) and last author (NP). A total of 1894 effect sizes were extracted and used in the final analysis. We extracted data on the effects of temperature on gamete traits (e.g., gamete size, quantity or quality), gonad traits (e.g., gonad size, gonadosomatic index) and reproductive output (Table S2). For each effect size, we recorded information on taxonomic family, class and phylum, fertilisation mode, which sex was exposed to the temperature change, whether temperature variation was natural or experimentally manipulated, the duration of exposure to the temperature change and the developmental stage during which individuals were exposed to the temperature change.

### 2.2 Effect Size Calculation

Data were extracted from tables, text or figures using WebPlotDigitizer (Rohatgi 2022). For studies using two or three discrete temperature treatments (e.g., low versus high temperatures), we extracted the mean and standard deviation and used these to calculate a biserial correlation coefficient and its sampling variance using the ‘escalc’ function in the ‘metafor’ package vers.4.6‐0 (Viechtbauer 2010). The ‘escalc’ function uses equation 12 from Jacobs and Viechtbauer (2017) to calculate the sampling variance. To account for shared‐control non‐independence, we first identified all cases where a control group was compared to several treatment groups. We then reduced the weight of the control group in the analyses by dividing the sample size of such a group by as many times that group has been compared to a treatment group before calculating effect sizes.

For studies where fertility was measured across a range of temperatures (i.e., where it was appropriate to treat temperature as a continuous variable), we calculated a Pearson's correlation coefficient. For any studies that only provided test statistics, such as a chi‐square test, we converted these to correlation coefficients, using the formulas provided in Borenstein et al. (2011). For both biserial correlation coefficients and Pearson's correlation coefficients, a positive value indicated higher fertility at higher temperatures, whereas a negative value indicated a reduction in fertility at higher temperatures.

In total, we obtained 1326 effect sizes for marine species, 473 effect sizes for freshwater species and 95 effect sizes for species that were neither freshwater nor marine (e.g., hypersaline or estuarine). This included 241 species from nine phyla with Chordata (38.2%) having the largest representation, followed by Arthropoda (20.7%) and Mollusca (17.2%) (Figure S2). All species in our dataset were ectotherms.

### 2.3 Data Analysis

**FIGURE 1:**
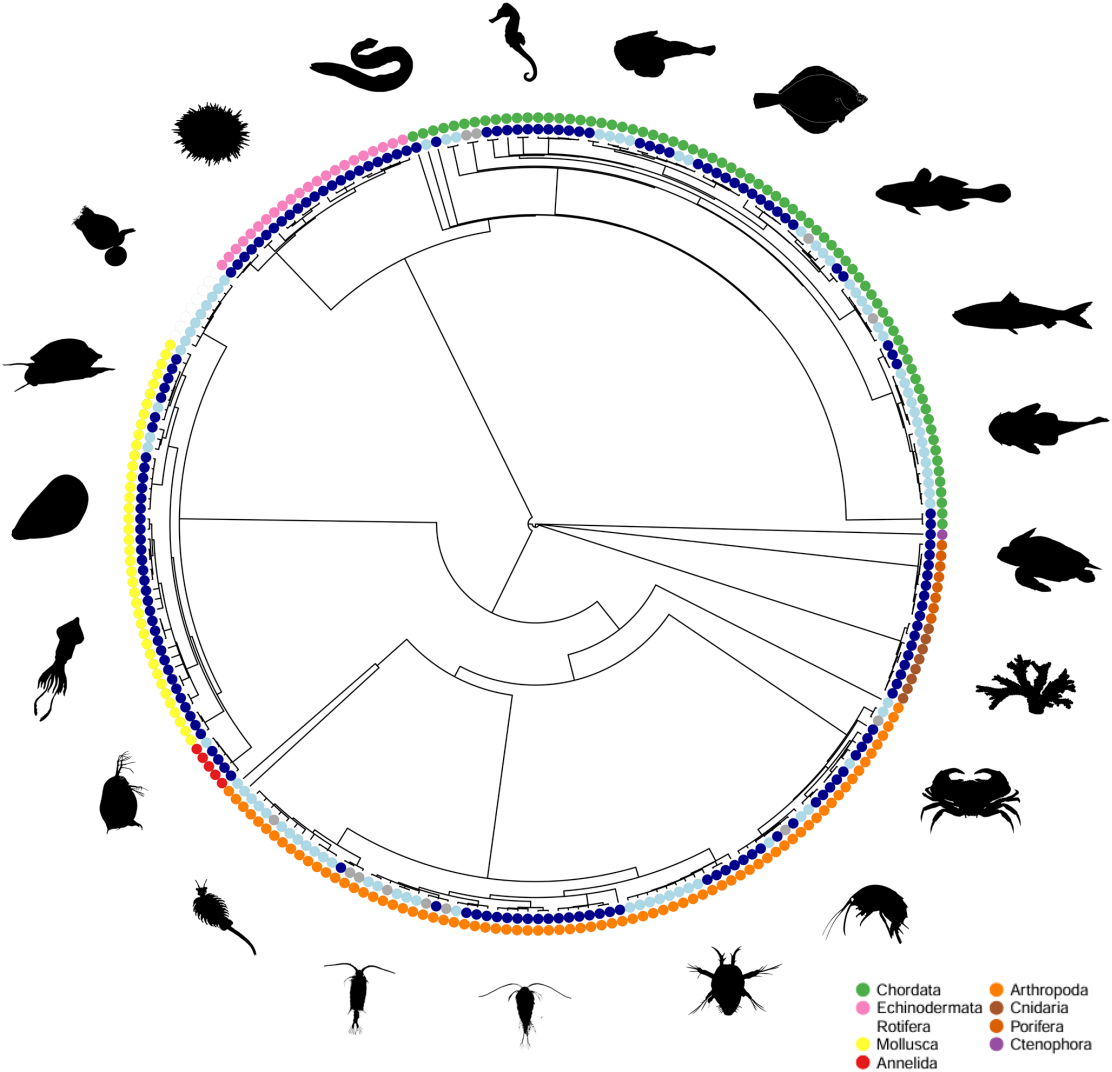
Phylogenetic tree of the species represented in this study. Light blue dots represent freshwater species (75 species), dark blue represents marine species (154 species) and dark grey dots represent other aquatic species (e.g., estuarine or hypersaline, 12 species). The other coloured dots represent the different phyla. Silhouettes around the phylogenetic tree are representative of some of the species included in the meta‐analysis for each taxon.

All analyses were completed in R version 4.3.1 (R Core Team 2023), and figures were generated using the ‘orchaRd’ package vers. 2 (Nakagawa et al. 2023) and the ‘ggplot2’ package vers. 3.5.1 (Wickham 2016). These analyses and all figures use a combination of biserial correlation coefficients (where means and standard deviations were available, *k* = 1583) and Pearson's correlation coefficients (*k* = 311). To construct a phylogenetic tree for the species included in our dataset, we used the tree for the 1191 species in the full systematic map (Dougherty et al. 2024) and then used the ‘set.diff’ and ‘drop.tip’ functions in the ‘ape’ package vers. 5.7‐1 (Paradis, Claude, and Strimmer 2004) to remove non‐aquatic species (Figure 1). The variance–covariance matrix representing the phylogenetic relatedness between all species in our dataset was used as a random effect in all of our meta‐analytic models described below (Paradis, Claude, and Strimmer 2004). We also included the following random effects in all models: ‘paper code’ to account for multiple effect sizes from the same study, ‘animal group ID’ to control for effect sizes from the same paper that used different groups of animals, ‘species ID’ to account for the same species being used across different papers, ‘shared control ID’ to account for effect sizes from the same paper which shared a control treatment and ‘effect size code’ as a unit level effect to measure residual heterogeneity (Viechtbauer 2010).

We first assessed the overall effect of temperature on fertility with a random effects model using the ‘rma.mv’ function in ‘metafor’. We estimated heterogeneity in our main effects model using *I* ^*2*^ as an estimate of the proportion of variance explained due to differences between the levels of a random effect (Nakagawa and Santos 2012). We also display prediction intervals on figures as a measure of heterogeneity (Cooper, Hedges, and Valentine 2019). We then tested a range of moderators that may influence the effect of temperature on fertility, using multi‐level meta‐regression models with the same random effects described above. For each moderator, we calculated a marginal *R* ^2^ to describe the percentage of heterogeneity explained by the moderator model compared to the main effects model. Statistical significance of moderators was determined using omnibus tests of meta‐regressions with intercepts. We obtained mean effect size estimates (*β*) and 95% confidence intervals for each level of the moderator by removing the intercept from the meta‐regression model. For statistically significant moderators with more than two levels, we then examined which factor levels differed from one another. We did this using the L argument in the ‘anova’ command to perform linear contrasts in meta‐regressions without an intercept.

We tested 10 categorical moderators and four continuous moderators (Table S1). We first looked at three categorical moderators to examine whether there were any differences in the effects of temperature on fertility that could be explained by the type of fertility trait being examined: (*i*) ‘sex of the trait’ (male, female, or both), (*ii*) ‘type of fertility trait’ (gamete, gonad or reproductive output), and (*iii*) 'sex exposed' (male, female, or both). The category ‘both’ for ‘sex of trait’ refers to combined fertility traits, influenced by both male and female fertility changes, such as fertilisation success. Sex of trait differs from the sex exposed to temperature as some papers only exposed one sex but measured traits that could be affected by both sexes. For fertility traits that could not be assigned to a specific sex, such as fertilisation success or offspring number (*k* = 479), we ran a model testing whether the effect on these fertility traits varied depending on whether only males, only females or both sexes had been exposed to increased temperature ('sex exposed'). This allowed us to test for additive or multiplicative effects of exposing both sexes to a higher temperature compared to just one sex.

Five categorical moderators and one continuous variable were used to test whether variation in the effect of temperature on fertility could be explained by the organism's physiological or life history traits: (*i*) ‘developmental stage of animal exposed’ to compare effects due to the exposure of gametes, embryos and juveniles, or adults, (*ii*) ‘habitat type’ to test for differences between freshwater and marine species, (*iii*) ‘fertilisation mode’ to test for differences between internal and external fertilisers, (*iv*) ‘phylum’ to test for different effects across taxonomic groups (*v*) ‘climate zone’ to test for differences among species from tropical, subtropical, temperate or polar regions and (*vi*) each species' thermal tolerance (CT_max_). To examine the relevance of thermal tolerance to effects on fertility, we subtracted the elevated temperature organisms were exposed to from that species' critical thermal limit (CT_max_), using data for 13 species from four phyla in our dataset from the GlobTherm database (Bennett et al. 2018; Figure S3). As with the main dataset, the majority of the effect sizes (*k* = 184 out of 250) came from Chordata species (*n* = 6), and four Mollusc species contributed 47 effect sizes. The other species were Echinoderms (*n* = 2, *k* = 18) and Arthropods (*n* = 1, *k* = 1). In addition to examining the effects of each of these moderators individually, we looked at the interaction between fertilisation mode and habitat type to test whether the effects of temperature on internal versus external fertilisers differed between freshwater and marine environments.

We then examined the effects of a study's experimental design, and the type of temperature exposure experienced by the organism, focusing on the duration of the temperature change, the magnitude of the temperature change, whether organisms experienced constant or fluctuating temperatures and whether the temperature variation was experimentally manipulated or experienced under natural conditions. We also looked at whether the interaction between the magnitude of temperature change and the duration of the temperature change influenced the effect of temperature on fertility.

Lastly, we examined sex‐specific effects using a series of multi‐level meta‐analytic models with interaction effects. More specifically, we tested the interaction between focal sex (male or female) and (*i*) ‘type of fertility trait’ (gamete, gonad or reproductive success), (*ii*) ‘fertilisation mode’ (internal or external fertilisation) and (*iii*) ‘developmental stage of animal exposed’ (gamete, early development or adulthood). Alluvial plots show the distribution of effect sizes across the moderator levels for these interactions (Figures S4–S6). For statistically significant interactions, we conducted a leave‐one‐out analysis to assess the stability of the interactions and determine if the results changed significantly when excluding one group. This was done using the ‘metafor’ and ‘dplyr’ packages vers. 1.1.4 for data manipulation (Wickham et al. 2023).

### 2.4 Publication Bias Tests

Given the well‐documented pattern that studies with statistically significant results or larger effects tend to be published earlier (Jennions and Møller 2002), we used a meta‐regression with mean‐centred publication year as a continuous moderator to test for time‐lag bias using the same random effects described above. We also used the square root of the inverse of the sample size for each effect size as a moderator to test for small‐study effects (Nakagawa et al. 2022).

### 2.5 Sensitivity Analyses

We chose to use effect sizes based on correlation coefficients rather than standardised mean differences for our analyses, because we were interested in examining the strength and direction of the relationship between temperature and fertility, rather than testing for differences between two groups. However, given that most of our effect sizes were based on means and standard deviations, we performed a sensitivity analysis to test whether our results are robust regardless of the type of effect size chosen. To this end, we re‐ran all of our models using standardised mean differences (Hedges' *g* effect sizes). Hedges' *g* is more robust to unequal sampling and small sample sizes than Cohen's *d* (Rosenberg, Rothstein, and Gurevitch 2013). Correlation coefficients or test statistics were converted to Hedges' *g* effect sizes using the equations provided by Borenstein et al. (2011).

To ensure our results were robust given non‐independence of effect sizes extracted from the same studies, we conducted cluster robust estimate of the variance models (RVE) for our moderator models using the ‘robust’ function in ‘metafor’ and compared them to the standard random effects models (Hedges, Tipton, and Johnson 2010). The RVE models provide a more conservative estimate of significance with larger standard errors and wider confidence intervals and can prevent Type 1 errors (Tanner‐Smith and Tipton 2014). Given that tolerance of exposure to a thermal stressor may depend on the duration of exposure (Bigelow 1921; Cerdá, Retana, and Cros 1998; Tang et al. 2000; Cerdá and Retana 2000; Armstrong, Tang, and Wang 2009; Rezende, Castañeda, and Santos 2014), we also conducted a sensitivity analysis using the duration of the temperature change as an additional fixed effect in our moderator models. These models only included 1362 out of the 1894 effect sizes in our full dataset, because we lacked information on the duration of temperature exposure for the remaining effect sizes; 28% of papers in our dataset did not report this information.

Lastly, we ran two additional multi‐level meta‐regression models to examine whether there were any systematic differences depending on how our effect sizes were calculated. The moderator for the first model was the final effect size type (biserial correlation coefficient or Pearson's correlation coefficient), and the moderator for the second one was the type of statistical test that the effect sizes were originally converted from (e.g., chi‐square test, Spearman's correlation). Both of these models included the same random effects as described above.

## 3 Results

Our dataset contained 1894 effect sizes from 276 studies on 241 species (Figure 1). Most of our data related to the effect of temperature on reproductive output (*k* = 911) and gamete traits (*k* = 763) with an additional 221 effect sizes on gonad traits. We report mean effect size estimates obtained from the statistical models with 95% confidence intervals in brackets. The statistical significance of moderators was determined using omnibus tests of meta‐regressions with intercepts.

### 3.1 Overall Effect of Temperature on Fertility

**FIGURE 2:**
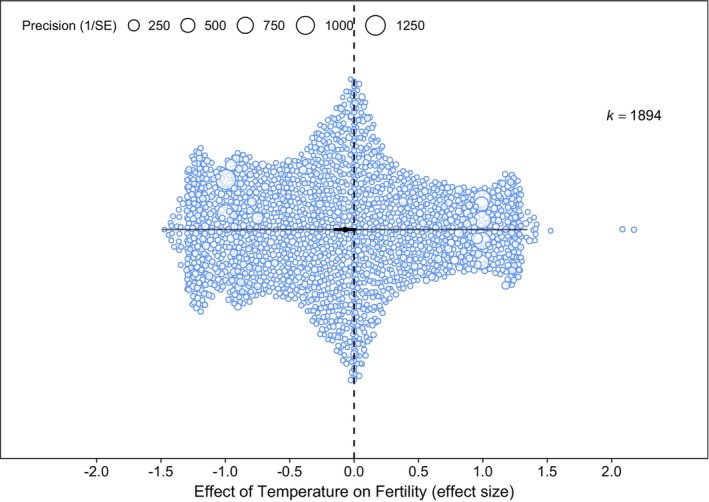
Mean effect size estimate for the effect of temperature on the fertility of aquatic animals (*k* = 1894, *n* = 276). The x‐axis shows the magnitude of the effect size (*r*_*b*_ and *r*) and the size of each point showing a measure of the standard error (1/SE). A negative effect size indicates that the fertility traits are affected negatively by increased temperature (e.g., reduced gamete quality), whereas a positive effect size indicates that fertility traits are positively affected by increased temperature (e.g., increased clutch size). The small black bars show the 95% confidence intervals (CI), and the longer thin bars show the 95% prediction intervals (PI). The dashed line indicates an effect size value of 0.

There was no evidence for an overall effect of temperature on the fertility of aquatic animals when using the standard random effects model (*r*_*b*_ = −0.07 [−0.16, 0.02], *p* = 0.11; Figure 2; Table 1). However, when using the robust variance estimate model, there was a statistically significant negative effect on fertility (Table S5). The total heterogeneity (*I* ^2^) for the main effects model was 99.9% with most of the variance attributed to effect size identity (69.7%) and between‐study differences (21.58%). Between‐species differences and between‐animal group differences accounted for 5.54% and 2.51% of the total heterogeneity, respectively, and phylogeny only accounted for 0.63%. Heterogeneity attributed to the sharing of a control treatment value across effect sizes accounted for < 0.01% (Table S3).

**TABLE 1:** Results of meta‐regression models assessing the effects of moderators on the effect of temperature on the fertility of aquatic animals. For each model, we provide information on moderator levels, including total effect sizes (*k*_total_), the number of effect sizes (*k*) for each level, the number of papers for each level (*n*) and marginal *R* ^2^, indicating the amount of heterogeneity explained by the moderator (*R* ^2^_mar_). The omnibus *Q* test of moderators (*Q*_m_) and its associated *p*‐value (*p*) were obtained from models with the intercept. Mean effect size estimates (*β*) and 95% confidence intervals (CI) were obtained from models without intercepts. Statistically significant *p*‐values and 95% confidence intervals differing from zero are shown in bold, whereas marginally nonsignificant results are shown in italics.

Moderator	Moderator levels (*k*)	*k*_total_	*n*	*R* ^2^_mar_ (%)	*Q*_m_	*p*	*β*	95% CI
Overall model		1894	276	—	—	0.11	−0.070	[−0.16, 0.02]
Fertility trait characteristics								
Trait category	Gonad traits (*k* = 221)	1894	52	0.55	6.44	**0.040**	0.010	[−0.15, 0.13]
	Gamete traits (*k* = 758)		150				−0.14	**[−0.23, −0.04]**
	Reproductive output (*k* = 915)		169				−0.036	[−0.13, 0.06]
Sex of fertility trait	Male (*k* = 473)	1894	92	0.34	3.46	0.18	−0.08	[−0.20, 0.04]
	Female (*k* = 766)		164				−0.03	[−0.12, 0.07]
	Both (*k* = 655)		114				−0.12	**[−0.23, −0.02]**
Sex exposed (for combined fertility traits)	Male (*k* = 24)	607	4	0.72	2.20	0.33	−0.16	[−0.64, 0.33]
	Female (*k* = 62)		19				−0.29	**[−0.58, −0.001]**
	Both (*k* = 521)		85				−0.08	[−0.24, 0.08]
Species characteristics								
Developmental stage	Adult (*k* = 1123)	1600	188	3.10	16.92	**< 0.001**	−0.07	**[−0.15, 0.001]**
	Early development (*k* = 73)		20				0.19	[−0.04, 0.42]
	Gamete (*k* = 404)		54				−0.32	**[−0.45, −0.18]**
Habitat type	Marine (*k* = 1326)	1894	176	0.17	0.95	0.62	−0.08	[−0.18, 0.01]
	Freshwater (*k* = 473)		89				−0.02	[−0.16, 0.11]
	Other (*k* = 95)		14				−0.13	[−0.42, 0.16]
Fertilisation mode	Internal (*k* = 658)	1894	124	0.59	3.55	*0.060*	−0.01	[−0.11, 0.08]
	External (*k* = 1236)		174				−0.13	**[−0.21, −0.05]**
Phylum	Annelida (*k* = 43)	1893	8	1.27	3.89	0.79	−0.18	[−0.69, 0.33]
	Arthropoda (*k* = 393)		86				−0.02	[−0.29, 0.25]
	Chordata (*k* = 722)		105				−0.13	[−0.43, 0.18]
	Cnidaria (*k* = 44)		13				−0.05	[−0.48, 0.39]
	Echinodermata (*k* = 258)		31				−0.20	[−0.54, 0.15]
	Mollusca (*k* = 325)		52				−0.08	[−0.41, 0.25]
	Porifera (*k* = 48)		4				0.07	[−0.44, 0.58]
	Rotifera (*k* = 60)		10				0.22	[−0.22, 0.65]
Climate zone	Mix (*k* = 315)	1894	65	0.40	2.61	0.62	−0.003	[−0.15, 0.14]
	Polar (*k* = 101)		10				−0.10	[−0.41, 0.22]
	Sub‐Tropical (*k* = 239)		46				−0.02	[−0.18, 0.14]
	Temperate (*k* = 742)		125				−0.12	**[−0.23, −0.02]**
	Tropical (*k* = 497)		74				−0.086	[−0.21, 0.04]
Thermal tolerance (CT_max_)		250	35	6.42	9.25	**0.002**	−0.03	**[−0.05, −0.01]**
Temperature change characteristics								
Duration of temperature change		1362	187	0.03	0.16	0.69	< 0.001	[−0.0003, 0.005]
Magnitude of temperature change		1894	276	0.03	0.34	0.56	−0.003	[−0.01, 0.01]
Temperature variability	Constant (*k* = 1406)	1836	196	0.23	1.53	0.21	−0.10	**[−0.20, −0.01]**
	Fluctuating (*k* = 430)		88				0.02	[−0.15, 0.11]
Source of temperature variation	Natural (*k* = 238)	1873	58	0.66	5.23	**0.022**	0.07	[−0.07, 0.22]
	Experimental (*k* = 1635)		229				−0.10	**[−0.19, −0.02]**

### 3.2 Species Physiology and Life History

**FIGURE 3:**
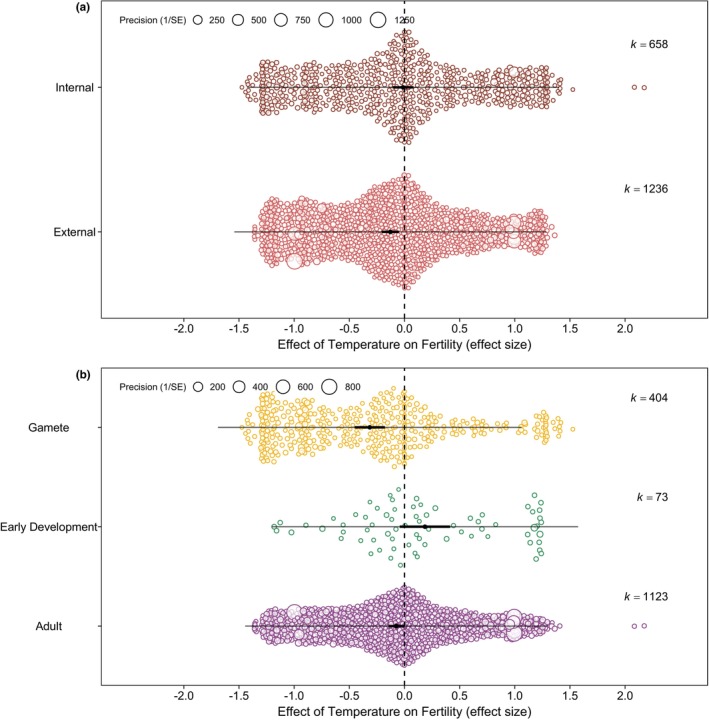
Results of meta‐regression on the effect of increased temperature on fertility for (a) different fertilisation modes (internal: *k* = 658, *n* = 124; external: *k* = 1236, *n* = 174) and (b) different development stages (adult: *k* = 1123, *n* = 188; early development: *k* = 73, *n* = 21; gametes: *k* = 404, *n* = 54). The small black bars show the 95% confidence intervals (CI), and the longer thin bars show the 95% prediction intervals (PI), and *k* refers to the number of effect sizes. The dashed line indicates an effect size value of 0. The *x*‐axis shows the magnitude of the effect size (*r*_*b*_ and *r*) and the size of each point showing a measure of the standard error (1/SE). The vertical placement on the y‐axis is arbitrary and varies based on the quantity of data points associated with a particular effect size value. There was a marginally nonsignificant difference between external and internal fertilisers (*p* = 0.060). Gametes were more vulnerable to warming than adults (*p* = 0.001) and early developmental stages (*p* < 0.001).

The effect of temperature on fertility did not differ among marine, freshwater or other aquatic species (Table 1; Figure S7). It also did not differ among phyla (Table 1; Figure S8). However, there was some evidence for a stronger negative effect of increased temperature on external than internal fertilisers (Figure 3a). Although this effect was marginally nonsignificant based on the omnibus test (*p* = 0.060), the mean effect size estimate for external fertilisers was negative (*r*_*b*_ = −0.13 [−0.21, −0.05]; Table 1) whereas for internal fertilisers the estimate was not statistically different from zero (*r*_*b*_ = −0.01 [−0.11, 0.08]; Table 1). There was also a significant interaction between fertilisation mode and habitat type (*Q*_m_ = 8.77, *p* = 0.033), indicating that the larger negative effects of increased temperature on external fertilisers are more pronounced in freshwater than marine species (Figure S9).

**FIGURE 4:**
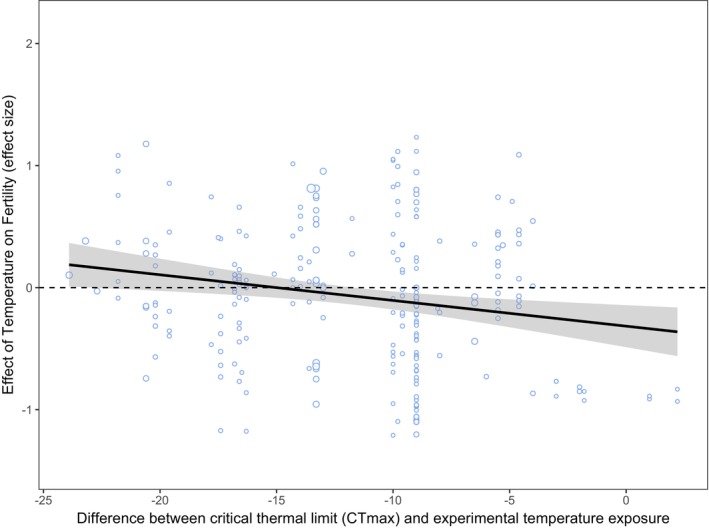
Meta‐regression showing how the effect on fertility varies depending on the difference between a species' critical thermal limit (CT_max_) and the experimental temperature they were exposed to (°C). The y‐axis shows the effect size estimate (*r*_*b*_ and *r*). On the *x*‐axis, the closer the value is to 0, the closer the experimental temperature of exposure is to the species' CTmax (*k* = 250, *n* = 35). The size of each point indicates the sample size of the effect size (*N*).

In addition, the effect of temperature on fertility was dependent on the developmental stage that was exposed to the temperature change (Table 1; Figure 3b): increased temperature had a stronger negative effect on gametes than adults (*Q*_m_ = 9.75, *p* = 0.002) and early developmental stages, such as embryos or juveniles (*Q*_m_ = 14.32, *p* < 0.001). Lastly, there was a larger reduction in fertility when organisms were exposed to temperatures that were closer to that species' critical thermal limit, CT_max_ (Table 1; Figure 4).

### 3.3 Sex‐Specific Effects

Increased temperature had a similar effect on the fertility of males, females and combined fertility traits that could not be assigned to a specific sex, such as fertilisation success (Table 1). For combined fertility traits, there was no evidence for additive or multiplicative effects of exposing both sexes to the temperature change compared to only males or females (Table 1; Figure S10).

The effect of temperature differed among fertility traits, with increased temperature having a stronger negative effect on gamete traits than gonad traits (*Q*_m_ = 3.68, *p =* 0.055) or reproductive output (*Q*_m_ = 4.58, *p =* 0.032), although this result was not robust using the robust variance estimate model (Table S5). There was no significant interaction between sex and the type of fertility trait indicating that gamete and gonad traits were similarly affected by increased temperature in males and females (*Q*_m_ = 2.44, *p* = 0.49). There was also no significant interaction between sex and fertilisation mode (*Q*_m_ = 5.58, *p* = 0.13), suggesting that male and female fertility traits were similarly affected by increased temperature in external and internal fertilisers.

Lastly, there was a significant interaction between sex and the developmental stage of the organism exposed (*Q*_m_ = 12.90, *p* = 0.024), which indicated that when the exposure to warming occurred during the gamete stage or adulthood, there was a stronger negative effect on female than male fertility (Table S7). This result was robust to the leave‐one‐group‐out analysis we conducted (*Q*_m_ = 8.32, *p* = 0.040).

### 3.4 Temperature Change Characteristics

Fertility was more negatively affected when temperature increases were the result of experimental manipulation rather than natural temperature variation, although we note that our sample sizes for this comparison were fairly unbalanced (Table 1; Figure S11). The effect of temperature on fertility was not influenced by the magnitude of the temperature increase (Table 1). Increased temperature also had a similar effect on fertility regardless of the duration of exposure to the higher temperature (Figure S12) and whether organisms experienced constant or fluctuating temperatures (Table 1). Lastly, there was no evidence of an interaction between the magnitude of the temperature change and the duration of the temperature change (*Q*_m_ = 4.11, *p* = 0.25).

### 3.5 Publication Bias Tests

We found no evidence of small‐study effects (*β* = −0.09 [−0.51, 0.34], *p* = 0.69; intercept = −0.05 [−0.18, 0.07], *p* = 0.40; *R* ^2^_mar_ = 0.02%; Figure S13). There was also no evidence of the decline effect where there is a tendency for smaller effect sizes over time (*β* = −0.004 [−0.01, 0.003], *p* = 0.24; intercept = −0.070 [−0.15, 0.01], *p* = 0.10; *R* ^2^_mar_ = 0.21%; Figure S14).

### 3.6 Sensitivity Analyses

Comparing the results of the models using correlation coefficients and standardised mean differences showed that most results were similar, with only one out of 17 models differing in statistical significance: fertility was more negatively affected in response to larger temperature increases when using standardised mean differences, whereas this effect was not statistically significant when using correlation coefficients (Table 1; Table S4).

There were no statistically significant differences between the types of effect sizes used in the statistical analysis, either biserial correlation coefficients or Pearson's correlation coefficients (*Q*_m_ = 0.99, *p* = 0.32). There were also no differences between the types of statistical tests that the effect sizes were originally converted from, such as chi‐square or Spearman's rank correlation (*Q*_m_ = 2.66, *p* = 0.45).

When duration of temperature exposure was included in our moderator models as a fixed effect, most of the results were qualitatively similar, but there were three models that differed in statistical significance compared to the models without duration of temperature exposure (Table S6). First, there was a statistically significant negative effect of temperature on the fertility on external fertilisers than internal fertilisers (*Q*_m_ = 6.66, *p* = 0.04). Second, there was no difference in the effect of temperature on different types of fertility traits (*Q*_m_ = 2.38, *p* = 0.50) when duration of temperature exposure was included in the model. Lastly, the previously significant difference between the effects of natural temperature variation and experimental manipulation was non‐significant (*Q*_m_ = 2.75, *p* = 0.25; Table 1; Figure S11). These differences between the moderator models with and without duration of temperature exposure as a fixed effect may be at least partly due to the fact that the former models only included 1362 out of the 1894 effect sizes in our full dataset.

## 4 Discussion

Our meta‐analysis of 1894 effect sizes across 276 studies and 241 species showed only a small overall negative effect of temperature on fertility in aquatic animals. We also identified a number of factors that influence vulnerability to warming. Interestingly, although all species in our dataset were ectotherms, we found that external fertilisers tend to be more negatively affected than internal fertilisers, especially in freshwater species. In addition, we found that male and female traits were similarly affected, although when the exposure to increased temperature occurred at the gamete stage or in adulthood, females suffered a greater reduction in fertility than males. This suggests that under some scenarios, female fertility is more sensitive to thermal stress than male fertility, challenging the prevailing view that high temperatures are most harmful to males (Chakir et al. 2002; Araripe et al. 2004; Vollmer et al. 2004; Rohmer et al. 2004; David et al. 2005; Nguyen, Bressac, and Chevrier 2013; Vasudeva, Deeming, and Eady 2014; Batista, Rocha, and Klaczko 2018; van Heerwaarden and Sgrò 2021; Santos et al. 2023). Our meta‐analysis reveals that this may not be the case, at least in aquatic animals, highlighting the need to rethink this widely held view. Overall, our work provides novel insights into how climate change may affect fertility and can improve our ability to make informed predictions about how certain species attributes may influence vulnerability in a warming world.

Aquatic organisms are predicted to be particularly vulnerable to the negative effects of climate change (Richardson and Poloczanska 2008), partly due to their slower acclimation rate to increased temperature compared to terrestrial organisms (Einum and Burton 2023; but see Noble et al. 2024). This slower rate of acclimation, and lower level of phenotypic plasticity, is likely an evolutionary outcome of more stable temperatures experienced by aquatic organisms because of the high heat capacity of water, as well as the natural temperature stratification of aquatic habitats, which provides thermal refuges (Einum and Burton 2023). Even though we expected that increased temperature would have detrimental effects on fertility, we initially found no overall directional effect of increased temperature on aquatic animal fertility (*β* = −0.07, *p* = 0.11) across the 241 aquatic species in our dataset. Nevertheless, when using the robust variance estimate model, there was indeed a small, statistically significant negative effect of temperature on fertility (*β* = −0.07, *p* = 0.021).

It is important to note that there was substantial variation in the magnitude and direction of thermal effects on fertility, with elevated temperatures resulting in improved fertility in some species (e.g., rotifer: Simonini and Prevedelli 2003; brine shrimp: Medina et al. 2007; grayling: Lahnsteiner and Kletzl 2012). The largest proportion of heterogeneity (*I* ^2^) in our study was attributed to variation among individual effect sizes. The high total heterogeneity limits the generalisability of the results and indicates that the effect of the temperature change is dependent on study‐specific factors and other potential sources of variation such as various characteristics of both the species and the temperature change itself. We therefore examined a range of moderators that could explain variation in the effect of temperature on fertility in aquatic animals, which we discuss in detail below—these moderators still only accounted for a small proportion of the observed heterogeneity (Table 1).

Firstly, we examined species characteristics relating to physiology and life history. We found some evidence suggesting that external fertilisers may be more vulnerable to warming than internal fertilisers, and the larger negative effects of increased temperature on external fertilisers were more pronounced in freshwater than marine species (interaction between fertilisation mode and habitat type). Finding a greater reduction in fertility in externally fertilising species would not be surprising if our analysis focused on endothermic species. Indeed, under such a scenario, we would expect this difference to be even more pronounced, because spermatozoa travelling through the reproductive tract of an endotherm, with a precisely regulated body temperature, are safeguarded from variation in ambient temperature (Walsh et al. 2019). However, given that our dataset only includes ectotherms, whose body temperature closely matches ambient temperature, it is interesting that we still found a pattern whereby internal fertilisers tend to be less vulnerable to temperature increases. This suggests that some aspect of the reproductive tract itself may offer protection to gametes from high temperature during internal fertilisation, even in species that do not maintain a stable body temperature. We propose this might be due to oviductal fluid in the reproductive tract of some ectotherms, which can moderate the harmful effects of high temperature on sperm performance and survival. A potential mechanism is that the composition of ions and proteins present in the oviductal fluid can stimulate sperm metabolism, improve motility and may have a range of other effects that buffer against thermal stress (Rosengrave et al. 2009). It would be interesting for future studies to test whether this potential pattern of increased vulnerability to increased temperature in externally fertilising species also applies to terrestrial organisms.

We found no consistent differences between freshwater and marine species in their vulnerability to warming. There were also no differences among species in different climate zones, even though it is generally thought that tropical species will be more vulnerable to increased temperature as they inhabit environments near their upper critical thermal limits (Comte and Olden 2017; van Heerwaarden and Sgrò 2021). Indeed, we found that organisms experienced a larger reduction in fertility when the temperatures they were exposed to were closer to their critical thermal limits (CT_max_). We were able to detect this effect of thermal tolerance even though information on CT_max_ was only available for a small subset of our full dataset (*k* = 250 from 35 studies on 13 species), and this finding still holds when using the robust variance estimate model.

Secondly, we examined several moderators relating to characteristics of the fertility trait. We found that increased temperature had a stronger negative effect on gamete traits than gonad traits or reproductive output, although this result did not hold when using the robust variance estimate model; this may be due to data for multiple fertility traits being reported in each paper. We also found that the effect of temperature on fertility was dependent on the developmental stage being exposed, with the gamete stage being most vulnerable. Due to their immature homeostatic and aerobic systems that control heat shock and repair responses (Dahlke et al. 2020), gametes have a lower thermal tolerance and are thus more vulnerable to high temperature than later developmental stages (Andronikov 1975; Jonsson and Jonsson 2009; Elliott and Elliott 2010; Adriaenssens et al. 2012; Jatteau et al. 2017; Foo and Byrne 2017). Breeding adults also have relatively narrow thermal safety margins compared to juveniles, due to the increased metabolic load caused by gamete production, which may reduce thermal tolerance because of a lack of full aerobic capacity (Dahlke et al. 2020). This is consistent with our finding that the juvenile stage is least sensitive to exposure to high temperature. However, we note that there was relatively little data available on the effects of temperature on fertility following exposure during early development, so we encourage more research on this understudied topic.

Thirdly, we focused on sex‐specific effects of increased temperature on fertility. A large body of literature has focused on the effects of temperature on sperm, and male fertility more generally, due to the prevailing view that males are more susceptible to thermal stress (e.g., Chakir et al. 2002; Araripe et al. 2004; Vollmer et al. 2004; Rohmer et al. 2004; David et al. 2005; Nguyen, Bressac, and Chevrier 2013; Vasudeva, Deeming, and Eady 2014; Batista, Rocha, and Klaczko 2018; van Heerwaarden and Sgrò 2021; Santos et al. 2023). Even though this prediction is based on studies that primarily use insect study systems (Walsh et al. 2019), it has generally been extended across taxa. Nevertheless, our meta‐analysis on 241 aquatic species does not support this prediction, as there was no significant difference in the effects of temperature on the fertility of males versus females. On the contrary, we found that when the exposure to warming occurs during the gamete stage or in adulthood, there is a stronger negative effect on female than male fertility (interaction between sex and developmental stage).

We also took advantage of our data on combined fertility traits (*k* = 605), such as fertilisation success or offspring number, to test for additive, compensatory or multiplicative effects of exposing both sexes to increased temperature versus only exposing males or females. If these effects are additive or multiplicative, we would expect the combined effect of exposing both sexes to result in a greater reduction in fertility than the independent effects on males and females (Pilakouta et al. 2024). We found no significant differences in fertility when one sex or both sexes were exposed to increased temperature. However, we note that this analysis was highly unbalanced due to the majority of studies exposing both sexes, so we cannot draw strong conclusions based on this result. We recommend that future research specifically investigates thermal effects on fertility when exposing only males, only females, and both sexes to high temperature across a range of taxa to address this knowledge gap.

Fourthly, we looked at various characteristics of the temperature change itself. Neither the duration of exposure to increased temperature nor the magnitude of the temperature change seemed to influence the severity of thermal effects on fertility. The absence of an effect of magnitude of temperature change might be due to the fact that studies using more extreme temperature increases only expose organisms for a short period of time (e.g., simulated heatwave), whereas studies using smaller temperature increases do so for an extended duration and sometimes for the whole lifespan (Ettinger‐Epstein et al. 2007; Yoneda et al. 2014). Nevertheless, we found no evidence of an interaction between the duration and magnitude of the temperature change. Fertility was more negatively affected when temperature increases were the result of experimental manipulation rather than natural temperature variation. We speculated that this might be due to differences in the variability of temperature (constant versus fluctuating), or the magnitude of the temperature increases used in experimental versus field‐based studies, but there was no evidence this was the case (*t* = 0.36, *p* = 0.72).

Finally, we note that meta‐analysis provides a framework for assessing any biases inherent in the existing literature. Indeed, we identified a clear taxonomic bias in this dataset with all 241 species being ectotherms. We currently lack studies examining the effects of temperature on reproduction in aquatic endotherms. This is likely due to the practical limitations and challenges of working with these taxa, but this information is crucial for advancing our understanding of how climate change may affect animal fertility, which is linked to population dynamics and may ultimately influence the likelihood of population extinction. Similarly, most of the effect sizes in our dataset were on marine species (*k* = 1326 out of *k*_total_ = 1894). We also acknowledge that since we used the systematic map by Dougherty et al. (2024) to identify relevant studies for our meta‐analysis, any limitations of that map also apply to this study, including the fact that it only contains papers published up to 24 August 2021.

In summary, our meta‐analysis shows that an increase in temperature does not consistently have detrimental effects on the fertility of aquatic animals and that its impact can vary depending on a number of factors. We found stronger negative effects of increased temperature on freshwater species with external than internal fertilisation, suggesting that the female's reproductive tract may have the capacity to moderate the effects of thermal stress, even in ectotherms that do not maintain a stable body temperature. We also found that gametes were more vulnerable to elevated temperature than later development stages. Lastly, females suffered a greater reduction in fertility than males when exposed to a higher temperature at the gamete stage or in adulthood. This contradicts the widely held view that male fertility is more sensitive to thermal stress and suggests that the effects of high temperature on female fertility may be underestimated and underappreciated. Using a powerful meta‐analytic approach with > 1800 effect sizes, our work provides valuable new insights on the effects of temperature on fertility in aquatic animals. Given that even a slight reduction in fertility can have consequences for population survival, it is critical to better understand and predict the effects of climate change on reproductive traits.

## Author Contributions

A.C. and N.P. conceived and designed the study. A.C., I.G., E.M. and C.H. screened the papers and extracted and collected data. A.C. and N.P. validated and checked the data. A.C. analysed the data with input from N.P. A.C. and N.P. wrote the manuscript.

## Conflicts of Interest

The authors declare no conflicts of interest.

## Supporting information


Data S1.

## Data Availability

All data and code used in this meta‐analysis and the associated R code are available from the Dryad Digital Repository https://doi.org/10.5061/dryad.gtht76hvb and https://doi.org/10.5281/zenodo.11003107.

## References

[ele70054-bib-0001] Adriaenssens, B. , R. van Damme , F. Seebacher , and R. S. Wilson . 2012. “Sex Cells in Changing Environments: Can Organisms Adjust the Physiological Function of Gametes to Different Temperatures?” Global Change Biology 18: 1797–1803.

[ele70054-bib-0002] Andronikov, V. B. 1975. “Heat Resistance of Gametes of Marine Invertebrates in Relation to Temperature Conditions Under Which the Species Exist.” Marine Biology 30: 1–11.

[ele70054-bib-0003] Araripe, L. O. , L. B. Klaczko , B. Moreteau , and J. R. David . 2004. “Male Sterility Thresholds in a Tropical Cosmopolitan Drosophilid, *Zaprionus indianus* .” Journal of Thermal Biology 29: 73–80.

[ele70054-bib-0004] Armstrong, J. W. , J. Tang , and S. Wang . 2009. “Thermal Death Kinetics of Mediterranean, Malaysian, Melon, and Oriental Fruit Fly (Diptera: Tephritidae) Eggs and Third Instars.” Journal of Economic Entomology 102: 522–532. 19449631 10.1603/029.102.0209

[ele70054-bib-0005] Barbraud, C. , and H. Weimerskirch . 2001. “Emperor Penguins and Climate Change.” Nature 411: 183–186. 11346792 10.1038/35075554

[ele70054-bib-0006] Batista, M. R. D. , F. B. Rocha , and L. B. Klaczko . 2018. “Altitudinal Distribution of Two Sibling Species of the *Drosophila tripunctata* Group in a Preserved Tropical Forest and Their Male Sterility Thermal Thresholds.” Journal of Thermal Biology 71: 69–73. 29301702 10.1016/j.jtherbio.2017.10.019

[ele70054-bib-0007] Baumann, H. , S. C. Talmage , and C. J. Gobler . 2012. “Reduced Early Life Growth and Survival in a Fish in Direct Response to Increased Carbon Dioxide.” Nature Climate Change 2: 38–41.

[ele70054-bib-0008] Bennett, J. M. , P. Calosi , S. Clusella‐Trullas , et al. 2018. “GlobTherm, a Global Database on Thermal Tolerances for Aquatic and Terrestrial Organisms.” Scientific Data 5: 180022. 29533392 10.1038/sdata.2018.22 PMC5848787

[ele70054-bib-0009] Bigelow, W. 1921. “The Logarithmic Nature of Thermal Death Time Curves.” Journal of Infectious Diseases 29: 528–536.

[ele70054-bib-0010] Borenstein, M. , L. V. Hedges , J. P. T. Higgins , and H. R. Rothstein . 2011. Introduction to Meta‐Analysis. Hoboken, NJ, USA: John Wiley & Sons.

[ele70054-bib-0011] Borenstein, M. , L. V. Hedges , J. P. T. Higgins , and H. R. Rothstein . 2021. Introduction to Meta‐Analysis. Hoboken, NJ, USA: John Wiley & Sons.

[ele70054-bib-0012] Cerdá, X. , and J. Retana . 2000. “Alternative Strategies by Thermophilic Ants to Cope With Extreme Heat: Individual Versus Colony Level Traits.” Oikos 89: 155–163.

[ele70054-bib-0013] Cerdá, X. , J. Retana , and S. Cros . 1998. “Critical Thermal Limits in Mediterranean Ant Species: Trade‐Off Between Mortality Risk and Foraging Performance.” Functional Ecology 12: 45–55.

[ele70054-bib-0014] Chakir, M. , A. Chafik , B. Moreteau , P. Gibert , and J. R. David . 2002. “Male Sterility Thermal Thresholds in Drosophila: *D. simulans* Appears More Cold‐Adapted Than Its Sibling *D. melanogaster* .” Genetica 114: 195–205. 12041832 10.1023/a:1015154329762

[ele70054-bib-0015] Chen, C.‐P. , and B.‐Y. Chen . 1992. “Effects of High Temperature on Larval Development and Metamorphosis of *Arachnoides placenta* (Echinodermata: Echinoidea).” Marine Biology 112: 445–449.

[ele70054-bib-0016] Ciannelli, L. , K. Bailey , and E. M. Olsen . 2015. “Evolutionary and Ecological Constraints of Fish Spawning Habitats.” ICES Journal of Marine Science 72: 285–296.

[ele70054-bib-0017] Comte, L. , and J. D. Olden . 2017. “Climatic Vulnerability of the World's Freshwater and Marine Fishes.” Nature Climate Change 7: 718–722.

[ele70054-bib-0018] Cooper, H. M. , L. V. Hedges , and J. C. Valentine , eds. 2019. The Handbook of Research Synthesis and Meta‐Analysis. 3rd ed. New York: Russell Sage Foundation.

[ele70054-bib-0019] Dahlke, F. T. , S. Wohlrab , M. Butzin , and H.‐O. Pörtner . 2020. “Thermal Bottlenecks in the Life Cycle Define Climate Vulnerability of Fish.” Science 369: 65–70. 32631888 10.1126/science.aaz3658

[ele70054-bib-0020] David, J. R. , L. O. Araripe , M. Chakir , et al. 2005. “Male Sterility at Extreme Temperatures: A Significant but Neglected Phenomenon for Understanding *Drosophila* Climatic Adaptations.” Journal of Evolutionary Biology 18: 838–846. 16033555 10.1111/j.1420-9101.2005.00914.x

[ele70054-bib-0021] Deutsch, C. A. , J. J. Tewksbury , R. B. Huey , et al. 2008. “Impacts of Climate Warming on Terrestrial Ectotherms Across Latitude.” Proceedings of the National Academy of Sciences of the United States of America 105: 6668–6672. 18458348 10.1073/pnas.0709472105 PMC2373333

[ele70054-bib-0022] Dougherty, L. R. , F. Frost , M. I. Maenpaa , et al. 2024. “A Systematic Map of Studies Testing the Relationship Between Temperature and Animal Reproduction.” Ecological Solutions and Evidence 5: e12303.

[ele70054-bib-0023] Einum, S. , and T. Burton . 2023. “Divergence in Rates of Phenotypic Plasticity Among Ectotherms.” Ecology Letters 26: 147–156. 36450612 10.1111/ele.14147 PMC10099672

[ele70054-bib-0024] Elliott, J. M. , and J. A. Elliott . 2010. “Temperature Requirements of Atlantic Salmon *Salmo salar* , Brown Trout *Salmo trutta* and Arctic Charr *Salvelinus alpinus* : Predicting the Effects of Climate Change.” Journal of Fish Biology 77: 1793–1817. 21078091 10.1111/j.1095-8649.2010.02762.x

[ele70054-bib-0025] Ettinger‐Epstein, P. , S. W. Whalan , C. N. Battershill , and R. De Nys . 2007. “Temperature Cues Gametogenesis and Larval Release in a Tropical Sponge.” Marine Biology 153: 171–178.

[ele70054-bib-0026] Foo, S. A. , and M. Byrne . 2017. “Marine Gametes in a Changing Ocean: Impacts of Climate Change Stressors on Fecundity and the Egg.” Marine Environmental Research 128: 12–24. 28237403 10.1016/j.marenvres.2017.02.004

[ele70054-bib-0027] Haddaway, N. R. , B. Macura , P. Whaley , and A. S. Pullin . 2018. “ROSES RepOrting Standards for Systematic Evidence Syntheses: Pro Forma, Flow‐Diagram and Descriptive Summary of the Plan and Conduct of Environmental Systematic Reviews and Systematic Maps.” Environmental Evidence 7: 7.

[ele70054-bib-0028] Han, J. , J.‐S. Lee , J. C. Park , A. Hagiwara , K.‐W. Lee , and J.‐S. Lee . 2020. “Effects of Temperature Changes on Life Parameters, Oxidative Stress, and Antioxidant Defense System in the Monogonont Marine Rotifer *Brachionus plicatilis* .” Marine Pollution Bulletin 155: 111062. 32469753 10.1016/j.marpolbul.2020.111062

[ele70054-bib-0029] Hedges, L. V. , E. Tipton , and M. C. Johnson . 2010. “Robust Variance Estimation in Meta‐ Regression With Dependent Effect Size Estimates.” Research Synthesis Methods 1: 39–65. 26056092 10.1002/jrsm.5

[ele70054-bib-0030] Jacobs, P. , and W. Viechtbauer . 2017. “Estimation of the Biserial Correlation and Its Sampling Variance for Use in Meta‐Analysis.” Research Synthesis Methods 8: 161–180. 27631635 10.1002/jrsm.1218

[ele70054-bib-0031] Jatteau, P. , H. Drouineau , K. Charles , L. Carry , F. Lange , and P. Lambert . 2017. “Thermal Tolerance of Allis Shad ( *Alosa alosa* ) Embryos and Larvae: Modeling and Potential Applications.” Aquatic Living Resources 30: 2.

[ele70054-bib-0032] Jennions, M. D. , and A. P. Møller . 2002. “Relationships Fade With Time: A Meta‐Analysis of Temporal Trends in Publication in Ecology and Evolution.” Proceedings of the Royal Society of London. Series B: Biological Sciences 269: 43–48. 10.1098/rspb.2001.1832 PMC1690867 11788035

[ele70054-bib-0033] Jonsson, B. , and N. Jonsson . 2009. “A Review of the Likely Effects of Climate Change on Anadromous Atlantic Salmon Salmo Salar and Brown Trout *Salmo trutta* , With Particular Reference to Water Temperature and Flow.” Journal of Fish Biology 75: 2381–2447. 20738500 10.1111/j.1095-8649.2009.02380.x

[ele70054-bib-0034] Lahnsteiner, F. , and M. Kletzl . 2012. “The Effect of Water Temperature on Gamete Maturation and Gamete Quality in the European Grayling (*Thymalus thymallus*) Based on Experimental Data and on Data From Wild Populations.” Fish Physiology and Biochemistry 38: 455–467. 21701820 10.1007/s10695-011-9526-8

[ele70054-bib-0035] Lin, Q. , J. Lu , Y. Gao , L. Shen , J. Cai , and J. Luo . 2006. “The Effect of Temperature on Gonad, Embryonic Development and Survival Rate of Juvenile Seahorses, *Hippocampus kuda* Bleeker.” Aquaculture 254: 701–713.

[ele70054-bib-0036] Mcdaniel, C. D. , R. K. Bramwell , J. L. Wilson , and B. Howarth . 1995. “Fertility of Male and Female Broiler Breeders Following Exposure to Elevated Ambient Temperatures1.” Poultry Science 74: 1029–1038. 10.3382/ps.0741029 7644414

[ele70054-bib-0037] Medina, G. R. , J. Goenaga , F. Hontoria , G. Cohen , and F. Amat . 2007. “Effects of Temperature and Salinity on Prereproductive Life Span and Reproductive Traits of Two Species of Artemia (Branchiopoda, Anostraca) From Argentina: *Artemia franciscana* and *A. persimilis* .” Hydrobiologia 579: 41–53.

[ele70054-bib-0039] Nakagawa, S. , M. Lagisz , M. D. Jennions , et al. 2022. “Methods for Testing Publication Bias in Ecological and Evolutionary Meta‐Analyses.” Methods in Ecology and Evolution 13: 4–21.

[ele70054-bib-0040] Nakagawa, S. , M. Lagisz , R. E. O'Dea , et al. 2023. “orchaRd 2.0: An R Package for Visualizing Meta‐Analyses With Orchard Plots.” Methods in Ecology and Evolution 14: 2003–2010.

[ele70054-bib-0041] Nakagawa, S. , and E. S. A. Santos . 2012. “Methodological Issues and Advances in Biological Meta‐Analysis.” Evolutionary Ecology 26: 1253–1274.

[ele70054-bib-0042] Nati, J. J. H. , M. B. S. Svendsen , S. Marras , et al. 2021. “Intraspecific Variation in Thermal Tolerance Differs Between Tropical and Temperate Fishes.” Scientific Reports 11: 21272. 34711864 10.1038/s41598-021-00695-8 PMC8553816

[ele70054-bib-0043] Nguyen, T. M. , C. Bressac , and C. Chevrier . 2013. “Heat Stress Affects Male Reproduction in a Parasitoid Wasp.” Journal of Insect Physiology 59: 248–254. 23262365 10.1016/j.jinsphys.2012.12.001

[ele70054-bib-0044] Noble, D. W. , F. Kar , A. Bush , F. Seebacher , and S. Nakagawa . 2024. “Reduced Plasticity and Variance in Physiological Rates of Ectotherm Populations Under Climate Change.” 10.32942/x2rs4w.

[ele70054-bib-0045] O'Dea, R. E. , M. Lagisz , M. D. Jennions , et al. 2021. “Preferred Reporting Items for Systematic Reviews and Meta‐Analyses in Ecology and Evolutionary Biology: A PRISMA Extension.” Biological Reviews 96: 1695–1722. 33960637 10.1111/brv.12721 PMC8518748

[ele70054-bib-0046] Pankhurst, N. W. , P. L. Munday , N. W. Pankhurst , and P. L. Munday . 2011. “Effects of Climate Change on Fish Reproduction and Early Life History Stages.” Marine and Freshwater Research 62: 1015–1026.

[ele70054-bib-0047] Paradis, E. , J. Claude , and K. Strimmer . 2004. “APE: Analyses of Phylogenetics and Evolution in R Language.” Bioinformatics 20: 289–290. 14734327 10.1093/bioinformatics/btg412

[ele70054-bib-0078] Pilakouta, N. , and A. Baillet . 2022. “Effects of Temperature on Mating Behaviour and Mating Success: A Meta‐Analysis.” Journal of Animal Ecology 91, no. 8: 1642–1650. 10.1111/1365-2656.13761. 35811382 PMC9541322

[ele70054-bib-0079] Pilakouta, N. , L. Sellers , R. Barratt , and A. Ligonniere . 2023. “The Consequences of Heatwaves for Animal Reproduction are Timing‐Dependent.” Functional Ecology 37, no. 9: 2425–2433. 10.1111/1365-2435.14386.

[ele70054-bib-0048] Pilakouta, N. , D. Allan , E. Moore , and A. A. Russell . 2024. “Chronic and Acute Thermal Stressors Have Non‐additive Effects on Fertility.” Proceedings of the Royal Society B 291: rspb20241086. 39288799 10.1098/rspb.2024.1086 PMC11407864

[ele70054-bib-0049] Pörtner, H. O. , and M. A. Peck . 2010. “Climate Change Effects on Fishes and Fisheries: Towards a Cause‐And‐Effect Understanding.” Journal of Fish Biology 77: 1745–1779. 21078088 10.1111/j.1095-8649.2010.02783.x

[ele70054-bib-0050] Pratchett, M. S. , L. K. Bay , P. C. Gehrke , et al. 2011. “Contribution of Climate Change to Degradation and Loss of Critical Fish Habitats in Australian Marine and Freshwater Environments.” Marine and Freshwater Research 62: 1062–1081.

[ele70054-bib-0051] R Core Team . 2023. R: A Language and Environment for Statistical Computing. Vienna, Austria: R Foundation for Statistical Computing. https://www.R‐project.org/.

[ele70054-bib-0052] Rezende, E. L. , L. E. Castañeda , and M. Santos . 2014. “Tolerance Landscapes in Thermal Ecology.” Functional Ecology 28: 799–809.

[ele70054-bib-0053] Richardson, A. J. , and E. S. Poloczanska . 2008. “Under‐Resourced, Under Threat.” Science 320: 1294–1295. 18535230 10.1126/science.1156129

[ele70054-bib-0054] Rohatgi, A. 2022. WebPlotDigitizer. Pacifica, California, USA: A Rohatgi.

[ele70054-bib-0055] Rohmer, C. , J. R. David , B. Moreteau , and D. Joly . 2004. “Heat Induced Male Sterility in *Drosophila melanogaster* : Adaptive Genetic Variations Among Geographic Populations and Role of the Y Chromosome.” Journal of Experimental Biology 207: 2735–2743. 15235002 10.1242/jeb.01087

[ele70054-bib-0056] Rosa, R. , M. Baptista , V. M. Lopes , et al. 2014. “Early‐Life Exposure to Climate Change Impairs Tropical Shark Survival.” Proceedings of the Royal Society B: Biological Sciences 281: 20141738. 10.1098/rspb.2014.1738 PMC4173694 25209942

[ele70054-bib-0057] Rosenberg, M. S. , H. R. Rothstein , and J. Gurevitch . 2013. “Effect Sizes: Conventional Choices and Calculations.” In Handbook of Meta‐Analysis in Ecology and Evolution, edited by J. Koricheva , J. Gurevitch , and K. Mengersen , 61–71. Princeton, NJ: Princeton Scholarship Online.

[ele70054-bib-0058] Rosengrave, P. , H. Taylor , R. Montgomerie , V. Metcalf , K. McBride , and N. J. Gemmell . 2009. “Chemical Composition of Seminal and Ovarian Fluids of Chinook Salmon ( *Oncorhynchus tshawytscha* ) and Their Effects on Sperm Motility Traits.” Comparative Biochemistry and Physiology. Part A, Molecular & Integrative Physiology 152: 123–129. 10.1016/j.cbpa.2008.09.009 18835457

[ele70054-bib-0059] Santos, M. A. , A. Grandela , M. A. Antunes , et al. 2023. “Sex and Population Differences Underlie Variation in Reproductive Success in a Warming Environment.” Evolution 77: 1842–1851. 37306280 10.1093/evolut/qpad104

[ele70054-bib-0060] Servili, A. , A. V. M. Canario , O. Mouchel , and J. A. Muñoz‐Cueto . 2020. “Climate Change Impacts on Fish Reproduction Are Mediated at Multiple Levels of the Brain‐Pituitary‐Gonad Axis.” General and Comparative Endocrinology 291: 113439. 32061640 10.1016/j.ygcen.2020.113439

[ele70054-bib-0061] Simonini, R. , and D. Prevedelli . 2003. “Effects of Temperature on Two Mediterranean Populations of *Dinophilus gyrociliatus* (Polychaeta: Dinophilidae).” Journal of Experimental Marine Biology and Ecology 291: 79–93.

[ele70054-bib-0062] Spector, T. D. , and S. G. Thompson . 1991. “The Potential and Limitations of Meta‐Analysis.” Journal of Epidemiology and Community Health 45: 89–92. 2072080 10.1136/jech.45.2.89 PMC1060723

[ele70054-bib-0063] Stubbs, J. L. , N. Marn , M. A. Vanderklift , S. Fossette , and N. J. Mitchell . 2020. “Simulated Growth and Reproduction of Green Turtles ( *Chelonia mydas* ) Under Climate Change and Marine Heatwave Scenarios.” Ecological Modelling 431: 109185.

[ele70054-bib-0064] Tang, J. , J. N. Ikediala , S. Wang , J. D. Hansen , and R. P. Cavalieri . 2000. “High‐Temperature‐Short‐Time Thermal Quarantine Methods.” Postharvest Biology and Technology 21: 129–145.

[ele70054-bib-0065] Tanner‐Smith, E. E. , and E. Tipton . 2014. “Robust Variance Estimation With Dependent Effect Sizes: Practical Considerations Including a Software Tutorial in Stata and spss: Robust Variance Estimation.” Research Synthesis Methods 5: 13–30. 26054023 10.1002/jrsm.1091

[ele70054-bib-0066] van Heerwaarden, B. , and C. M. Sgrò . 2021. “Male Fertility Thermal Limits Predict Vulnerability to Climate Warming.” Nature Communications 12: 2214. 10.1038/s41467-021-22546-w PMC8044094 33850157

[ele70054-bib-0067] Vasudeva, R. , D. C. Deeming , and P. E. Eady . 2014. “Developmental Temperature Affects the Expression of Ejaculatory Traits and the Outcome of Sperm Competition in *Callosobruchus maculatus* .” Journal of Evolutionary Biology 27: 1811–1818. 24891122 10.1111/jeb.12431

[ele70054-bib-0068] Viechtbauer, W. 2010. “Conducting Meta‐Analyses in R With the Metafor Package.” Journal of Statistical Software 36: 1–48.

[ele70054-bib-0069] Vollmer, J. H. , P. Sarup , C. W. Kærsgaard , J. Dahlgaard , and V. Loeschcke . 2004. “Heat and Cold‐Induced Male Sterility in *Drosophila buzzatii*: Genetic Variation Among Populations for the Duration of Sterility.” Heredity 92: 257–262. 14679393 10.1038/sj.hdy.6800405

[ele70054-bib-0070] Walsh, B. S. , S. R. Parratt , A. A. Hoffmann , et al. 2019. “The Impact of Climate Change on Fertility.” Trends in Ecology & Evolution 34: 249–259. 30635138 10.1016/j.tree.2018.12.002

[ele70054-bib-0071] Wang, W. W.‐Y. , and A. R. Gunderson . 2022. “The Physiological and Evolutionary Ecology of Sperm Thermal Performance.” Frontiers in Physiology 13: 754830. 35399284 10.3389/fphys.2022.754830 PMC8987524

[ele70054-bib-0072] Wickham, H. 2016. ggplot2. Cham: Springer International Publishing.

[ele70054-bib-0073] Wickham, H. , R. François , L. Henry , K. Müller , and D. Vaughan . 2023. “dplyr: A Grammar of Data Manipulation.” R Package Version 1.1.2. Computer Software.

[ele70054-bib-0074] Wild, S. , M. Krützen , R. W. Rankin , W. J. E. Hoppitt , L. Gerber , and S. J. Allen . 2019. “Long‐Term Decline in Survival and Reproduction of Dolphins Following a Marine Heatwave.” Current Biology 29: R239–R240. 30939303 10.1016/j.cub.2019.02.047

[ele70054-bib-0075] Wudarski, J. , K. Ustyantsev , L. Glazenburg , and E. Berezikov . 2019. “Influence of Temperature on Development, Reproduction and Regeneration in the Flatworm Model Organism, *Macrostomum lignano* .” Zoological Letters 5: 7. 30805201 10.1186/s40851-019-0122-6 PMC6371448

[ele70054-bib-0076] Yao, Z. , L. W. Crim , G. F. Richardson , and C. J. Emerson . 2000. “Motility, Fertility and Ultrastructural Changes of Ocean Pout ( *Macrozoarces americanus* L.) Sperm After Cryopreservation.” Aquaculture 181: 361–375.

[ele70054-bib-0077] Yoneda, M. , H. Kitano , H. Tanaka , et al. 2014. “Temperature‐ and Income Resource Availability‐Mediated Variation in Reproductive Investment in a Multiple‐Batch‐Spawning Japanese Anchovy.” Marine Ecology Progress Series 516: 251–262.

