# Supplementary material for: Predicting the Effects of Climate Change on the Fertility of Aquatic Animals Using a Meta‐Analytic Approach
Source: Ecol Lett. 2024 Dec 31;28(1):e70054. doi: 10.1111/ele.70054 (PMC11687342; doi:10.1111/ele.70054)
Supplement: Supplementary file 1 — Data S1. [file ELE-28-0-s001.pdf]

Supplementary Tables and Figures

Supplementary Table 1. Table showing all variables used as moderators in the meta-regressions and their descriptions.

**Continuous moderators**	**Description**
Duration of temperature change	The period of time the organisms were exposed to the temperature changes in days
Magnitude of temperature change	The magnitude of the temperature changes the organisms were exposed to (low temperature minus high temperature)
Thermal tolerance	Species critical thermal maximum extracted from the GlobTherm database minus the maximum temperature organisms were exposed to
Publication year	Year of publication as indicated on the journal article

**Categorical moderators**	**Category**	**Description**
Sex of trait	Female	Female fertility traits, e.g. egg size, ovary length
	Male	Male fertility traits, e.g. sperm length, testes length, sperm motility
	Both	Combined fertility traits, e.g. number of fertilised eggs, hatchling number, number of offspring
Sex exposed for combined fertility traits	Females only	Effect sizes for combined fertility traits where only females were exposed to the temperature change
	Males only	Effect sizes for combined fertility traits where only males were exposed to the temperature change
	Both	Effect sizes for combined fertility traits where both males and females were exposed to the temperature change
Trait category	Gamete Traits	Effect sizes for traits relating to organisms' gametes e.g. egg mass, sperm velocity
	Gonad Traits	Effect sizes for traits relating to organisms' gonads e.g. ovary mass, testes length
	Reproductive Output	Effect sizes for traits relating to organisms' reproductive output e.g. number of hatchlings, fertilised egg number
Marine vs Freshwater	Marine	Species which inhabit marine environments
	Freshwater	Species which inhabit freshwater environments
	Other	Species which inhabit other aquatic environments such as hypersaline pools
Fertilisation mode	Internal	Species which reproduce via internal fertilisation
	External	Species which reproduce via external fertilisation
Developmental Stage	Gametes	Organisms exposed to temperature changes as gametes
	Early Development	Organisms exposed to temperature changes in early development e.g. larval stages, juveniles
	Adults	Organisms exposed to temperature change as adults
Constant or fluctuating	Constant	Temperature changes kept at a constant temperature after change
	Fluctuating	Temperature changes which fluctuate throughout the studies

Stressor Variation	Experimental	measuring the effects of experimentally manipulated temperature change
	Natural	measuring effects of natural temperature change
Phylum	Arthropoda	
	Annelida	
	Chordata	
	Mollusca	
	Echinodermata	
	Porifera	
	Cnidaria	
	Rotifera	
	Ctenophora	
Climate Zone	Mix	Species whose usual range covers a range of climate zones
	Temperate	Species whose usual range is in temperate climates
	Sub-tropical	Species whose usual range is in sub-tropical climates
	Tropical	Species whose usual range is in tropical regions
	Polar	Species whose usual range is in polar regions

Supplementary Table 2. Table showing included and excluded trait types extracted from papers and their descriptions.

Included trait types	Category	Description
Gamete traits	Gamete size	unfertilised egg diameter, sperm length etc
	Gamete quantity	number of eggs, sperm concentration etc.
	Gamete quality	sperm velocity, egg lipid content, egg/sperm abnormalities etc.
Gonad traits	Gonad size	gonadosomatic index, testes length etc.
	Gonad quality	mainly includes frequency of mature spermatozoa/ eggs in testes on ovaries
Reproductive output	Any traits post-fertilisation success e.g. clutch size, hatchling number, fertilisation success etc.	
**Excluded trait types**	**Description**	
Excluded gonad traits	Any traits to do with the development stage/time or maturity of the gonads such as frequency of yolk vesicle stage, frequency of primary yolk globule stage, frequency of secondary yolk globule stage, frequency of spermatogonia, frequency of spermatocytes, frequency of spermatids, Testosterone levels, estradiol levels.	
Other	Seasonal temperature variation across the breeding season (e.g. comparing data from one temperature within breeding season and one temperature from a non-breeding period).	

Supplementary Table 3. I^2 values for all meta-regression models using correlation coefficients (*r_b and r)*. I^2 Total %: This represents the total proportion of variability across the studies that is due to heterogeneity. I^2 animal group %: This refers to the proportion of heterogeneity within papers. I^2 Between %: This statistic measures the proportion of heterogeneity that is between different papers. I^2 Phylogenetic %: This statistic represents the proportion of variability explained by phylogenetic relationships among species. It indicates the extent to which evolutionary relatedness contributes to the heterogeneity in effect sizes. I^2 Species %: this measures the proportion of variability attributable to differences between species. It shows how much of the heterogeneity is due to species-specific effects. I^2 Effect Size %: This refers to the proportion of variability in the observed effect sizes that is due to differences between effect sizes.

**Moderator**	**I^2 total (%)**	**I^2 Animal Group (%)**	**I^2 Between (%)**	**I^2 Phylogenetic (%)**	**I^2 Species (%)**	**I2 Shared Control (%)**	**I^2 Effect size (%)**
**Overall model**	99.93	2.51	21.58	0.63	5.54	<0.001	69.67
**Fertility trait characteristics**							
Trait category	99.91	2.40	21.13	0.44	6.07	<0.001	69.87
Sex of fertility trait	99.91	2.20	21.73	0.57	5.47	<0.001	69.94
Sex exposed (for combined fertility traits)	99.89	17.51	11.17	2.07	4.00	<0.001	65.13
**Species characteristics**							
Developmental stage	99.90	1.76	20.99	<0.001	7.83	<0.001	69.32
Habitat type	99.91	2.40	21.33	0.64	6.10	<0.001	69.44
Fertilisation mode	99.92	2.50	21.64	<0.001	5.84	<0.001	69.94
Phylum	99.87	2.40	20.74	4.30	5.34	<0.001	67.10
Climate zone	99.89	2.34	20.87	0.43	6.53	<0.001	69.72
Thermal tolerance	99.65	22.11	1.38	0.99	13.15	12.51	49.52
**Temperature change characteristics**							
Duration of temperature change	99.93	8.79	20.65	1.11	6.50	<0.001	62.87
Magnitude of temperature change	99.92	2.48	21.49	0.69	5.68	<0.001	69.59
Temperature variability	99.91	2.29	22.05	0.63	4.88	<0.001	70.05
Source of temperature variation	99.90	3.11	20.95	0.53	5.17	<0.001	70.15
**Interactions**							

Sex × Fertilisation mode	99.91	<0.001	28.43	<0.001	2.50	<0.001	68.98
Sex × Development stage	99.86	<0.001	26.13	1.17	0.00	<0.001	72.56
Sex × Trait category	99.94	<0.001	23.43	<0.001	3.65	<0.001	72.85
Habitat type × Fertilisation mode	99.91	3.11	21.91	<0.001	5.57	<0.001	69.31
Magnitude × Duration of temperature change	99.92	8.87	20.86	0.93	6.02	<0.001	63.25

Supplementary Table 4. Results of meta-regression models using standardised mean differences assessing the effects of moderators on the effect of temperature on the fertility of aquatic animals. For each model, we provide information on moderator levels, including the number of effect sizes (*k*) for each level, total effect sizes (k_{total}), and marginal R^2, indicating the amount of heterogeneity explained by the moderator (R^2_{mar}). The omnibus Q test of moderators (Q_m) and its associated P-value (*P*) were obtained from models with the intercept. Point estimates (*β*) and 95% confidence intervals (CI) were obtained from models without intercepts. Significant P-values and 95% confidence intervals differing from zero are shown in bold, marginally non-significant results are shown in italics. Moderator names in italics indicate models which differ from the correlation

**Moderator**	**Moderator levels (*k*)**	k_{total}	***n***	R^2_{mar} **(%)**	Q_m	***P***	***β***	**95% CI**
Overall model		1894	276	–	–	*0.069*	-0.19	[-0.39, 0.01]
**Fertility trait characteristics**								
Trait category	Gonad traits (*k*=221)	1894	52	1.35	17.04	**<0.001**	0.06	[-0.31, 0.43]
	Gamete traits (*k*=758)		150				-0.49	**[-0.73, -0.24]**
	Reproductive output (*k*=915)		169				-0.001	[-0.24, 0.24]
Sex of fertility trait	Male (*k*=473)	1894	92	0.07	0.78	0.68	-0.19	[-0.49, 0.12]
	Female (*k*=766)		164				-0.13	[-0.37, 0.11]
	Both (*k*=655)		114				-0.26	[-0.54, 0.02]
Sex exposed (for combined fertility traits)	Male (*k*=24)	607	4	0.39	0.83	0.66	-0.07	[-1.73, 1.60]
	Female (*k*=62)		19				-0.57	[-1.39, 0.25]
	Both (*k*=521)		85				-0.17	[-0.58, 0.24]
**Species characteristics**								
Developmental stage	Adult (*k*=1123)	1600	188	2.40	13.43	**0.001**	-0.18	[-0.43, 0.07]
	Early development (*k*=73)		20				0.74	**[0.03, 1.46]**
	Gamete (*k*=404)		54				-0.74	**[-1.20, -0.28]**
Habitat type	Marine (*k*=1326)	1894	176	0.02	0.95	0.95	-0.18	[-0.43, 0.07]
	Freshwater (*k*=473)		89				-0.18	[-0.54, 0.18]
	Other (*k*=95)		14				-0.33	[-1.26, 0.60]

Fertilisation mode	Internal (*k*=658)	1894	124	0.03	0.15	0.70	-0.14	[-0.45, 0.16]
	External (*k*=1236)		174				-0.21	[-0.47, -0.04]
Phylum	Annelida (*k*=43)	1893	8	0.72	3.77	0.81	-0.17	[-1.43, 1.09]
	Arthropoda (*k*=393)		86				-0.11	[-0.49, 0.26]
	Chordata (*k*=722)		105				-0.32	[-0.64, 0.01]
	Cnidaria (*k*=44)		13				0.31	[-0.64, 1.25]
	Echinodermata (*k*=258)		31				-0.43	[-0.97, 0.12]
	Mollusca (*k*=325)		52				-0.13	[-0.59, 0.33]
	Porifera (*k*=48)		4				0.21	[-1.30, 1.71]
	Rotifera (*k*=60)		10				0.30	[-0.77, 1.36]
Climate zone	Mix (*k*=315)	1894	65	0.38	2.27	0.69	-0.10	[-0.51, 0.31]
	Polar (*k*=101)		10				-0.12	[-1.10, 0.85]
	Sub-Tropical (*k*=239)		46				0.09	[-0.39, 0.56]
	Temperate (*k*=742)		125				-0.26	[-0.55, 0.03]
	Tropical (*k*=497)		74				-0.31	[-0.68, 0.06]
Thermal tolerance		250	35	4.85	7.99	**0.005**	-0.06	**[-0.10, -0.02]**
**Temperature change characteristics**								
Duration of temperature change		1362	187	0.04	0.22	0.64	<0.001	[-0.0009, 0.002]
Magnitude of temperature change		1894	276	0.55	5.75	**0.02**	-0.04	**[-0.07, -0.01]**
Temperature variability	Constant (*k*=1406)	1836	196	0.47	2.66	0.10	-0.28	**[-0.54, -0.02]**
	Fluctuating (*k*=430)		88				0.06	[-0.31, 0.44]
Source of temperature variation	Natural (*k*=238)	1873	58	0.96	6.45	**0.011**	0.33	[-0.11, 0.78]
	Experimental (*k*=1635)		229				-0.29	**[-0.50, -0.07]**

Supplementary Table 5. Results of Robust Variance Estimate (RVE) meta-regression models assessing the effects of moderators on the effect of temperature on the fertility of aquatic animals. For each model, we provide information on moderator levels, including total effect sizes (k_{total}), the number of effect sizes (*k*) for each level, the number of papers for each level (*n*), and marginal R^2, indicating the amount of heterogeneity explained by the moderator (R^2_{mar}). The omnibus *F* test of moderators (*F*) and its associated P-value (*P*) were obtained from models with the intercept. Mean effect size estimates (*β*) and 95% confidence intervals (CI) were obtained from models without intercepts. Statistically significant P-values and 95% confidence intervals differing from zero are shown in bold, whereas marginally nonsignificant results are shown in italics. Moderator names in italics indicate models which differ from the correlation meta-regression models.

**Moderator**	**Moderator levels (*k*)**	**k_{total}**	***n***	**R^2_{mar} (%)**	***F***	***P***	***β***	**95% CI**
Overall model		1894	276	–	–	**0.021**	-0.070	[-0.13, -0.01]
**Fertility trait characteristics**								
Trait category	Gonad traits (*k*=221)	1894	52	0.55	2.07	0.13	0.010	[-0.15, 0.13]
	Gamete traits (*k*=758)		150				-0.14	**[-0.23, -0.05]**
	Reproductive output (*k*=915)		169				-0.036	[-0.12, 0.05]
Sex of fertility trait	Male (*k*=473)	1894	92	0.34	1.80	0.17	-0.08	[-0.18, 0.02]
	Female (*k*=766)		164				-0.03	[-0.11, 0.05]
	Both (*k*=655)		114				-0.12	**[-0.21, -0.04]**
Sex exposed (for combined fertility traits)	Male (*k*=24)	607	4	0.72	1.43	0.25	-0.16	[-0.46, 0.14]
	Female (*k*=62)		19				-0.29	**[-0.52, -0.06]**
	Both (*k*=521)		85				-0.08	[-0.19, 0.04]
**Species characteristics**								
Developmental stage	Adult (*k*=1123)	1600	188	3.10	7.44	**<0.001**	-0.07	**[-0.14, -0.003]**
	Early development (*k*=73)		20				0.19	[-0.10, 0.47]
	Gamete (*k*=404)		54				-0.32	**[-0.45, -0.18]**
Habitat type	Marine (*k*=1326)	1894	176	0.17	0.48	0.62	-0.09	**[-0.15, -0.02]**
	Freshwater (*k*=473)		89				-0.02	[-0.15, 0.11]

	Other (*k*=95)		14				-0.13	[-0.34, 0.08]
Fertilisation mode	Internal (*k*=658)	1894	124	0.59	3.83	0.052	-0.01	[-0.10, 0.08]
	External (*k*=1236)		174				-0.13	**[-0.20, -0.05]**
Phylum	Annelida (*k*=43)	1893	8	1.27	1.48	0.17	-0.18	[-0.50, 0.14]
	Arthropoda (*k*=393)		86				-0.02	[-0.15, 0.10]
	Chordata (*k*=722)		105				-0.13	[-0.23, -0.03]
	Cnidaria (*k*=44)		13				-0.05	[-0.32, 0.23]
	Echinodermata (*k*=258)		31				-0.20	[-0.36, -0.03]
	Mollusca (*k*=325)		52				-0.08	[-0.20, 0.04]
	Porifera (*k*=48)		4				0.07	[-0.10, 0.23]
	Rotifera (*k*=60)		10				0.22	[-0.12, 0.54]
Climate zone	Mix (*k*=315)	1894	65	0.40	0.87	0.48	-0.003	[-0.12, 0.12]
	Polar (*k*=101)		10				-0.10	[-0.35, 0.16]
	Sub-Tropical (*k*=239)		46				-0.02	[-0.17, 0.13]
	Temperate (*k*=742)		125				-0.12	**[-0.21, -0.03]**
	Tropical (*k*=497)		74				-0.086	[-0.21, 0.04]
Thermal tolerance		250	35	6.42	6.41	**0.016**	-0.03	**[-0.05, -0.01]**
**Temperature change characteristics**								
Duration of temperature change		1362	187	0.03	0.13	0.72	<0.001	[-0.0004, 0.005]
Magnitude of temperature change		1894	276	0.03	0.11	0.75	-0.003	[-0.02, 0.01]
Temperature variability	Constant (*k*=1406)	1836	196	0.23	1.30	0.26	-0.10	**[-0.18, -0.02]**
	Fluctuating (*k*=430)		88				-0.02	[-0.13, 0.09]
Source of temperature variation	Natural (*k*=238)	1873	58	0.66	4.15	**0.04**	0.07	[-0.07, 0.22]
	Experimental (*k*=1635)		229				-0.10	**[-0.17, -0.03]**

Table 6. Results of meta-regression models with duration of exposure as a fixed effect assessing the effects of moderators on the effect of temperature on the fertility of aquatic animals. For each model, we provide information on moderator levels, including total effect sizes (k_{total}), the number of effect sizes (*k*) for each level, the number of papers for each level (*n*), and marginal R^2, indicating the amount of heterogeneity explained by the moderator (R^2_{mar}). The omnibus Q test of moderators (Q_m) and its associated P-value (*P*) were obtained from models with the intercept. Mean effect size estimates (*β*) and 95% confidence intervals (CI) were obtained from models without intercepts. Statistically significant P-values and 95% confidence intervals differing from zero are shown in bold, whereas marginally nonsignificant results are shown in italics.

Moderator	Moderator levels (*k*)	k_{total}	*n*	R^2_{mar} (%)	Q_m	*P*	*β*	95% CI
**Fertility trait characteristics**								
Trait category + Duration of temperature change	Gonad traits (*k*=179)	1362	41	0.28	2.38	0.50	0.04	[-0.21, 0.14]
	Gamete traits (*k*=584)		109				-0.14	**[-0.27, -0.01]**
	Reproductive output (*k*=599)		104				-0.08	[-0.21, 0.05]
	Duration of temperature change		187				0.0001	[-0.0003, 0.0005]
Sex of fertility trait + Duration of temperature change	Male (*k*=398)	1362	74	0.47	3.54	0.32	-0.13	[-0.27, 0.02]
	Female (*k*=459)		96				-0.04	[-0.17, 0.098]
	Both (*k*=505)		87				-0.14	**[-0.28, -0.003]**
	Duration of temperature change		187				0.0001	[-0.0003, 0.0005]
Sex exposed (for combined fertility traits) + Duration of temperature change	Male (*k*=24)	470	4	1.25	2.74	0.43	-0.15	[-0.66, 0.36]
	Female (*k*=55)		16				-0.32	[-0.64, 0.008]
	Both (*k*=391)		62				-0.05	[-0.25, 0.15]
	Duration of temperature change		81				0.0002	[-0.0008, 0.0004]
**Species characteristics**								
Developmental stage + Duration of temperature change	Adult (*k*=708)	1199	128	4.13	16.97	**<0.001**	-0.05	[-0.16, 0.05]
	Early development (*k*=53)		15				0.24	[-0.04, 0.52]
	Gamete (*k*=366)		48				-0.34	**[-0.48, -0.19]**
	Duration of temperature change		174				0.0001	[-0.0003, 0.0005]

Habitat type + Duration of temperature change	Marine (*k*=971)	1362	124	0.04	0.20	0.98	-0.10	[-0.23, 0.03]
	Freshwater (*k*=365)		62				-0.10	[-0.27, 0.08]
	Other (*k*=26)		3				-0.04	[-0.64, 0.56]
	Duration of temperature change		187				0.0001	[-0.0003, 0.0005]
Fertilisation mode + Duration of temperature change	Internal (*k*=343)	1362	59	1.53	6.66	**0.036**	0.036	[-0.10, 0.18]
	External (*k*=1019)		140				-0.17	**[-0.27, -0.08]**
	Duration of temperature change		187				0.0001	[-0.0003, 0.0005]
Phylum + Duration of temperature change	Annelida (*k*=16)	1362	6	1.43	1.85	0.99	0.004	[-0.71, 0.72]
	Arthropoda (*k*=226)		45				0.016	[-0.37, 0.41]
	Chordata (*k*=512)		77				-0.12	[-0.57, 0.32]
	Cnidaria (*k*=33)		7				-0.26	[-0.86, 0.34]
	Echinodermata (*k*=243)		27				-0.24	[-0.72, 0.24]
	Mollusca (*k*=274)		41				-0.09	[-0.56, 0.39]
	Porifera (*k*=48)		4				0.05	[-0.60, 0.69]
	Rotifera (*k*=10)		1				0.18	[-0.78, 1.13]
	Duration of temperature change		187				0.0000	[-0.0004, 0.0005]
Climate zone + Duration of temperature change	Mix (*k*=220)	1362	44	0.94	4.43	0.49	0.02	[-0.15, 0.19]
	Polar (*k*=46)		5				-0.07	[-0.50, 0.36]
	Sub-Tropical (*k*=181)		34				-0.06	[-0.25, 0.13]
	Temperate (*k*=522)		84				-0.18	**[-0.31, -0.04]**
	Tropical (*k*=393)		51				-0.14	[-0.29, 0.02]
	Duration of temperature change		187				0.0001	[-0.0003, 0.0005]
Thermal tolerance + Duration of temperature change	Thermal tolerance	194	25	7.03	7.49	**0.02**	-0.03	**[-0.05, -0.005]**
	Duration of temperature change						0.0009	[-0.003, 0.0016]
**Temperature change characteristics**								

Magnitude of temperature change + Duration of temperature change	Magnitude of temperature change	1362	187	0.18	1.34	0.51	-0.01	[-0.21, 0.02]
	Duration of temperature change						0.0001	[-0.0003, 0.0005]
Temperature variability + Duration of temperature change	Constant (*k*=1060)		141				-0.10	[-0.21, -0.02]
	Fluctuating (*k*=257)	1317	49	0.027	0.14	0.93	-0.096	[-0.29, 0.10]
	Duration of temperature change		182				0.0001	[-0.0004, 0.0005]
Source of temperature variation + Duration of temperature change	Natural (*k*=135)		24				-0.02	[-0.29, 0.25]
	Experimental (*k*=1206)	1341	166	0.54	2.75	0.25	-0.12	**[-0.24, -0.01]**
	Duration of temperature change		186				0.0002	[-0.0003, 0.0008]

Supplementary Table 7. Results of interaction meta-regression models assessing the effects of moderators on the effect of temperature on the fertility of aquatic animals. For each model, we provide information on moderator levels, including total effect sizes (k_{total}), the number of effect sizes (*k*) for each level, the number of papers for each level (*n*), and marginal R^2, indicating the amount of heterogeneity explained by the moderator (R^2_{mar}). The omnibus *Q* test of moderators (Q_m) and its associated P-value (*P*) were obtained from models with the intercept. Mean effect size estimates (β) and 95% confidence intervals (CI) were obtained from models without intercepts. Statistically significant P-values and 95% confidence intervals differing from zero are shown in bold, whereas marginally nonsignificant results are shown in italics.

**Moderator**	**Moderator levels (*k*)**	k_{total}	***n***	R^2_{mar} **(%)**	Q_m	***P***	β	**95% CI**
**Sex specific interactions**								
Sex of fertility trait × Trait category	Female Gonad traits (*k*=76)	902	28	0.39	2.44	0.49	0.010	[-0.19, 0.18]
	Female Gamete traits (*k*=354)		97				-0.14	**[-0.25, -0.04]**
	Male Gonad traits (*k*=89)		28				-0.045	[-0.23, 0.14]
	Male Gamete traits (*k*=383)		71				-0.13	**[-0.24, -0.02]**
Sex of fertility trait × Fertilisation mode	Male External (*k*=302)	1239	71	1.30	5.58	0.13	-0.17	**[-0.28, -0.05]**
	Female External(*k*=458)		94				-0.09	[-0.19, 0.01]
	Male Internal (*k*=171)		22				0.07	[-0.14, 0.27]
	Female Internal (*k*=308)		73				0.01	[-0.11, 0.13]
Sex of fertility trait × Developmental stage	Adult Male (*k*=265)	607	56	3.12	12.90	**0.02**	-0.001	[-0.15, 0.15]
	Adult Female (*k*=552)		124				-0.08	[-0.20, 0.04]
	Early Development Male (*k*=25)		7				0.20	[-0.18, 0.58]
	Early Development Female (*k*=41)		12				0.22	[-0.08, 0.51]
	Gamete Male (*k*=168)		27				-0.27	**[-0.48, -0.05]**
	Gamete Female (*k*=52)		5				-0.37	[-0.76, 0.03]
**Species characteristics interactions**								

Habitat type × Fertilisation mode	Marine External (*k*=911)	1799	127	1.39	8.77	**0.032**	-0.10	**[-0.19, -0.01]**
	Freshwater External (*k*=253)		41				-0.22	**[-0.39, -0.05]**
	Marine Internal (*k*=415)		69				-0.09	[-0.21, 0.04]
	Freshwater Internal (*k*=415)		48				0.11	[-0.43, 0.27]
**Temperature change characteristics interactions**								
Duration of temperature change × magnitude of temperature change		1362	187	0.49	4.11	0.25	<0.001	[-0.000, 0.0001]

Supplementary Table 8. PRISMA EcoEvo checklist

Section and Topic	Item #	Checklist item	Location where item is reported
**TITLE**			
Title	1	Identify the report as a systematic review.	Abstract Line 44
**ABSTRACT**			
Abstract	2	See the PRISMA 2020 for Abstracts checklist.	Line 40-51
**INTRODUCTION**			
Rationale	3	Describe the rationale for the review in the context of existing knowledge.	Line 53-69
Objectives	4	Provide an explicit statement of the objective(s) or question(s) the review addresses.	Line 79-112
**METHODS**			
Eligibility criteria	5	Specify the inclusion and exclusion criteria for the review and how studies were grouped for the syntheses.	Supplementary Table 2 Line 124 to Line 133
Information sources	6	Specify all databases, registers, websites, organisations, reference lists and other sources searched or consulted to identify studies. Specify the date when each source was last searched or consulted.	Systematic map by Dougherty et al. 2024. Line 116-117
Search strategy	7	Present the full search strategies for all databases, registers and websites, including any filters and limits used.	Dougherty et al. 2024
Selection process	8	Specify the methods used to decide whether a study met the inclusion criteria of the review, including how many reviewers screened each record and each report retrieved, whether they worked independently, and if applicable, details of automation tools used in the process.	Supplementary Table 2 and Line 135-140 and 148
Data collection process	9	Specify the methods used to collect data from reports, including how many reviewers collected data from each report, whether they worked independently, any processes for obtaining or confirming data from study investigators, and if applicable, details of automation tools used in the process.	Line 135-157

Data items	10a	List and define all outcomes for which data were sought. Specify whether all results that were compatible with each outcome domain in each study were sought (e.g. for all measures, time points, analyses), and if not, the methods used to decide which results to collect.	Supplementary Table 1 and 2
	10b	List and define all other variables for which data were sought (e.g. participant and intervention characteristics, funding sources). Describe any assumptions made about any missing or unclear information.	Supplementary Table 1. Line 130 – 134 and 140-145
Study risk of bias assessment	11	Specify the methods used to assess risk of bias in the included studies, including details of the tool(s) used, how many reviewers assessed each study and whether they worked independently, and if applicable, details of automation tools used in the process.	Line 135-157
Effect measures	12	Specify for each outcome the effect measure(s) (e.g. risk ratio, mean difference) used in the synthesis or presentation of results.	Line 148-157
Synthesis methods	13a	Describe the processes used to decide which studies were eligible for each synthesis (e.g. tabulating the study intervention characteristics and comparing against the planned groups for each synthesis (item #5)).	Supplementary Table 2 Line 138 –145. Data Spreadsheet
	13b	Describe any methods required to prepare the data for presentation or synthesis, such as handling of missing summary statistics, or data conversions.	Line 131 -134 and 148 -165
	13c	Describe any methods used to tabulate or visually display results of individual studies and syntheses.	Line 173-189
	13d	Describe any methods used to synthesize results and provide a rationale for the choice(s). If meta-analysis was performed, describe the model(s), method(s) to identify the presence and extent of statistical heterogeneity, and software package(s) used.	Line 173-298
	13e	Describe any methods used to explore possible causes of heterogeneity among study results (e.g. subgroup analysis, meta-regression).	Line 195-296
	13f	Describe any sensitivity analyses conducted to assess robustness of the synthesized results.	Line 258-296
Reporting bias assessment	14	Describe any methods used to assess risk of bias due to missing results in a synthesis (arising from reporting biases).	Line 258-264
Certainty assessment	15	Describe any methods used to assess certainty (or confidence) in the body of evidence for an outcome.	Line 269-296
**RESULTS**			
Study selection	16a	Describe the results of the search and selection process, from the number of records identified in the search to the number of studies included in the review, ideally using a flow diagram.	Supplementary Figure 1
	16b	Cite studies that might appear to meet the inclusion criteria, but which were excluded, and explain why they were excluded.	Supplementar

			y Table 9
Study characteristics	17	Cite each included study and present its characteristics.	Supplementary File and Data Spreadsheet
Risk of bias in studies	18	Present assessments of risk of bias for each included study.	N/A
Results of individual studies	19	For all outcomes, present, for each study: (a) summary statistics for each group (where appropriate) and (b) an effect estimate and its precision (e.g. confidence/credible interval), ideally using structured tables or plots.	Data Spreadsheet and Table 1
Results of syntheses	20a	For each synthesis, briefly summarise the characteristics and risk of bias among contributing studies.	N/A
	20b	Present results of all statistical syntheses conducted. If meta-analysis was done, present for each the summary estimate and its precision (e.g. confidence/credible interval) and measures of statistical heterogeneity. If comparing groups, describe the direction of the effect.	Results Section and Table 1. Line 298 - 403
	20c	Present results of all investigations of possible causes of heterogeneity among study results.	Results section Line 306 - 403
	20d	Present results of all sensitivity analyses conducted to assess the robustness of the synthesized results.	Supplementary Table 3,4,5 and 6. Line 380 - 406
Reporting biases	21	Present assessments of risk of bias due to missing results (arising from reporting biases) for each synthesis assessed.	Results – Publication Bias Section Line 373-377
Certainty of evidence	22	Present assessments of certainty (or confidence) in the body of evidence for each outcome assessed.	Figures- confidence intervals and prediction intervals. Table 1
**DISCUSSION**			
Discussion	23a	Provide a general interpretation of the results in the context of other evidence.	Line 406-537

	23b	Discuss any limitations of the evidence included in the review.	Line 538-549
	23c	Discuss any limitations of the review processes used.	Line 546- 549
	23d	Discuss implications of the results for practice, policy, and future research.	Line 420-422 and 559-561
**OTHER INFORMATION**			
Registration and protocol	24a	Provide registration information for the review, including register name and registration number, or state that the review was not registered.	Line 116
	24b	Indicate where the review protocol can be accessed, or state that a protocol was not prepared.	N/A
	24c	Describe and explain any amendments to information provided at registration or in the protocol.	N/A
Support	25	Describe sources of financial or non-financial support for the review, and the role of the funders or sponsors in the review.	N/A
Competing interests	26	Declare any competing interests of review authors.	Line 568
Availability of data, code and other materials	27	Report which of the following are publicly available and where they can be found: template data collection forms; data extracted from included studies; data used for all analyses; analytic code; any other materials used in the review.	Data availability Statement Line 21 -24

Supplementary Table 9. Table of excluded studies, with author names, titles, publication year and exclusion reason as well as an assigned unique study ID.

Paper code	Paper	Title	Publication year	Exclusion Reason
TEMP4	Abdoli et al, 2005	Influence of female age, body size and environmental conditions on annual egg production of the bullhead	2005	No relevant/useable data/fertility traits
TEMP9	Abozaid et al, 2012	Elevated Temperature Applied during Gonadal Transformation Leads to Male Bias in Zebrafish (Danio rerio)	2012	No relevant/useable data/fertility traits
TEMP20	Aguilar-Alberola, J. A.; Mesquita-Joanes, F.	Breaking the temperature-size rule: Thermal effects on growth, development and fecundity of a crustacean from temporary waters	2014	No relevant/useable data/fertility traits
TEMP37	Aktas, M.et al.	Off-season maturation and spawning of Penaeus semisulcatus by eyestalk ablation and/or temperature-photoperiod regimes	2003	No relevant/useable data/fertility traits
HUM10	Albrizio et al., 2019	Male reproductive physiology of Paracentrotus lividus: monthly and seasonal evaluations to study the effects of the environment on gonadal development and sperm cells kinetic	2019	No useable temperature data
TEMP60	Amarasinghe, P. B.et al.	The effect of temperature, and food quantity and quality on the growth and development rates in laboratory-cultured copepods and cladocerans from a Sri Lankan reservoir	1997	No relevant/useable data/fertility traits
TEMP83	APPLETON, C.; ERIKSSON, I.	THE INFLUENCE OF FLUCTUATING ABOVE-OPTIMAL TEMPERATURE REGIMES ON THE FECUNDITY OF BIOMPHALARIA-PFEIFFERI (MOLLUSCA, PLANORBIDAE	1984	No useable temperature data
TEMP106	Aston, R. J.	EFFECT OF TEMPERATURE ON LIFE CYCLE GROWTH AND FECUNDITY OF BRANCHIURA SOWERBYI (OLIGOCHAETA - TUBIFICIDAE)	1968	Missing data
TEMP147	Ban, 1994	EFFECT OF TEMPERATURE AND FOOD CONCENTRATION ON POSTEMBRYONIC DEVELOPMENT, EGG-PRODUCTION AND ADULT BODY-SIZE OF CALANOID COPEPOD EURYTEMORA-AFFINIS	1994	No useable temperature data

TEMP177	Beltran-Pardo et al, 2015	Tolerance to Gamma Radiation in the Tardigrade Hypsibius dujardini from Embryo to Adult Correlate Inversely with Cellular Proliferation	2015	No relevant/useable data/fertility traits
TEMP199	Biernacka & Davies, 1994	THE EFFECTS OF TEMPERATURE AND FEEDING REGIME ON HERMAPHRODITISM AND GAMETOGENESIS OF NEPHELOPSIS-OBSCURA, A FRESH-WATER PREDATORY LEECH	1994	No relevant/useable data/fertility traits
TEMP200	Billard, 1968	TEMPERATURE INFLUENCE ON DURATION AND EFFECTS OF POECILIA RETICULATA GUPPY SPERMATOGENESIS	1968	No relevant/useable data/fertility traits
TEMP233	Boryshpolets et al, 2009	Pre-spawning water temperature affects sperm respiration and reactivation parameters in male carps	2009	No relevant/useable data/fertility traits
TEMP245	Brackenbury & Appleton, 1991	EFFECT OF CONTROLLED TEMPERATURES ON GAMETOGENESIS IN THE GASTROPODS PHYSA-ACUTA (PHYSIDAE) AND BULINUS-TROPICUS (PLANORBIDAE	1991	No relevant/useable data/fertility traits
TEMP253	Breton et al	EFFECTS OF PHOTOPERIOD AND TEMPERATURE ON PLASMA GONADOTROPIN AND SPERMATOGENESIS IN RAINBOW-TROUT SALMO-GAIRDNERII RICHARDSON	1977	Missing data
TEMP254	Breton et al, 1980	TEMPERATURE AND REPRODUCTION IN TENCH - EFFECT OF A RISE IN THE ANNUAL TEMPERATURE REGIME ON GONADOTROPIN LEVEL, GAMETOGENESIS AND SPAWNING .1. THE MALE	1980	No useable temperature data
TEMP264	Brugnano et al	Fecundity and development of the bentho-pelagic copepod Pseudocyclops umbraticus: effects of temperature	2014	Missing data
TEMP277	Burghard, W.; Maier, G.	The effect of temperature on mating duration in the freshwater cyclopoid copepod Cyclops vicinus (Uljanin, 1875)	2000	No relevant/useable data/fertility traits
TEMP317	Carre, D.; Carre, C. m. a.	Origin of germ cells, sex determination, and sex inversion in medusae of the genus Clytia (Hydrozoa, leptomedusae): The influence of temperature	2000	No relevant/useable data/fertility traits
TEMP327	Caselle, J. E.et al.	Geographic variation in density, demography, and life history traits of a harvested, sex-changing, temperate reef fish	2011	No relevant/useable data/fertility traits

TEMP337	Celada, J. D.et al.	Effects of different thermal treatments on the maternal incubation efficiency of the astacid crayfish Austropotamobius pallipes (Lereboullet, 1858) under controlled conditions	2001	No relevant/useable data/fertility traits
TEMP399	Clarke, N.et al.	Exposure to Treated Sewage Effluent Disrupts Reproduction and Development in the Seasonally Breeding Ramshorn Snail (Subclass: Pulmonata, Planorbarius corneus)	2009	No relevant/useable data/fertility traits
TEMP401	Claska, M.; Gilbert, J. m. m.	The effect of temperature on the response of Daphnia to toxic cyanobacteria	1998	No relevant/useable data/fertility traits
TEMP436	Cowgill, U. M.et al.	FECUNDITY AND LONGEVITY OF CERIODAPHNIA-DUBIA AFFINIS IN RELATION TO DIET AT 2 DIFFERENT TEMPERATURES	1985	Missing data
TEMP441	Cuco, A. P.et al.	Toxicity of two fungicides in Daphnia: is it always temperature-dependent?	2016	No relevant/useable data/fertility traits
OSM69	Cunha et al.	Predicting amphipods' brood size variation in brackish environments: an empirical model for Corophium multisetosum Stock, 1952 (Corophiidae) in Ria de Aveiro (NW Portugal)	2000	Missing data
TEMP451	Dadras, H.et al.	The in vitro effect of temperature on motility and antioxidant response of common carp Cyprinus carpio spermatozoa	2016	Seasonal
TEMP461	de Alvarenga, E. R.; de Franca, L. R.	Effects of Different Temperatures on Testis Structure and Function, with Emphasis on Somatic Cells, in Sexually Mature Nile Tilapias (Oreochromis niloticus)	2008	No relevant/useable data/fertility traits
TEMP481	Delorme, N. J.; Sewell, M. A.	Effects of warm acclimation on physiology and gonad development in the sea urchin Evechinus chloroticus	2016	No relevant/useable data/fertility traits
OSM73	Devreker et al.	Effects of salinity, temperature and individual variability on the reproduction of Eurytemora affinis (Copepoda; Calanoida) from the Seine estuary: A laboratory study	2009	No relevant/useable data/fertility traits
TEMP516	Dudycha, J. m. m.	A multi-environment comparison of senescence between sister species of Daphnia	2003	Missing data
TEMP522	Dvoretsky, V. G.; Dvoretsky, A. G. m. j.	Life cycle of Oithona similis (Copepoda: Cyclopoida) in Kola Bay (Barents Sea	2009	Seasonal

TEMP523	Dzyuba, B.et al.	Sperm motility of the Nile tilapia (Oreochromis niloticus): Effects of temperature on the swimming characteristics	2019	Missing data
TEMP528	EGAMI, N.	AN AUTORADIOGRAPHIC EXAMINATION OF RATE OF SPERMATOGENESIS AT DIFFERENT TEMPERATURES IN FISH ORYZIAS LATIPES	1967	No relevant/useable data/fertility traits
TEMP535	Ellis, C. D.et al.	Geographic and environmental drivers of fecundity in the European lobster (Homarus gammarus)	2015	No relevant/useable data/fertility traits
TEMP572	Ferraz, E. D.; Cerqueira, V. R.	INFLUENCE OF TEMPERATURE ON GONADAL MATURATION OF THE MALE COMMON SNOOK Centropomus undecimalis	2010	No relevant/useable data/fertility traits
TEMP583	Fivizzani, A. J.; Porter, D. A.	SEASONAL-VARIATION IN THE INFLUENCE OF TEMPERATURE ON THE GAMETOGENIC CYCLE OF FUNDULUS-DIAPHANUS	1982	seasonal
OSM97	Fong et al.	The effect of salinity and temperature on spawning and fertilization in the zebra mussel Dreissena polymorpha (Pallas) from North America	1995	Missing data
TEMP588	Foo, S. A.et al.	Adaptive capacity of the sea urchin Heliocidaris erythrogramma to ocean change stressors: responses from gamete performance to the juvenile	2016	Missing data
TEMP593	Franco, S. C.et al.	The effects of rearing temperature on reproductive conditioning of stalked barnacles (Pollicipes pollicipes)	2015	No relevant/useable data/fertility traits
TEMP613	Galindo et al	REPRODUCTIVE INVESTMENT OF SEVERAL ROTIFER SPECIES	1993	Missing data
TEMP621	Ganias, K.et al.	Maternal versus environmental constraints on the oocyte size of a marine pelagophil fish	2015	No relevant/useable data/fertility traits
TEMP645	Gilroy, C. E.; Litvak, M. K.	Swimming kinematics and temperature effects on spermatozoa from wild and captive shortnose sturgeon (Acipenser brevirostrum)	2019	No relevant/useable data/fertility traits
TEMP666	Gonzalez-Bernat, M. J.et al.	Effects of reduced seawater pH on fertilisation, embryogenesis and larval development in the Antarctic seastar Odontaster validus	2013	No useable temperature data

TEMP668	Goos, H.; Consten, D. m. n.	Stress adaptation, cortisol and pubertal development in the male common carp, Cyprinus carpio	2002	No relevant/useable data/fertility traits
TEMP691	GREENWOOD, P.	THE INFLUENCE OF AN OIL DISPERSANT CHEMSERVE OSE-DH ON THE VIABILITY OF SEA-URCHIN GAMETES - COMBINED EFFECTS OF TEMPERATURE, CONCENTRATION AND EXPOSURE TIME ON FERTILIZATION	1983	No relevant/useable data/fertility traits
TEMP727	Han et al	Why is Diaphanosoma (Crustacea: Ctenopoda) so common in the tropics? Influence of temperature and food on the population parameters of Diaphanosoma dubium, and a hypothesis on the nature of tropical cladocerans	2011	No relevant/useable data/fertility traits
TEMP730	Hansen, O. J.et al.	Importance of broodstock holding temperature on fecundity and egg quality in three groups of photo-manipulated Atlantic cod broodstock	2013	No useable temperature data
TEMP742	Havardsson, B.et al.	The effect of astaxanthin in feed and environmental temperature on carotenoid concentration in the gonads of the green sea urchin Strongylocentrotus droebachiensis Muller	1999	No relevant/useable data/fertility traits
TEMP792	Hoang, T.et al.	Observations on growth, sexual maturity and spawning performance of pond-reared Penaeus merguiensis	2002	Missing data
TEMP804	Hong, B. C.; Shurin, J. B.	Latitudinal variation in the response of tidepool copepods to mean and daily range in temperature	2015	No relevant/useable data/fertility traits
TEMP824	Huang, W. P.; Chou, L. S.	TEMPERATURE EFFECT ON DEVELOPMENT AND REPRODUCTION OF THE ANDRODIOECIOUS CLAM SHRIMP, EULIMNADIA BRAUERIANA (BRANCHIOPODA: SPINICAUDATA)	2015	No relevant/useable data/fertility traits
TEMP825	Huang, W. P.; Chou, L. S.	Temperature effects on life history traits of two sympatric branchiopods from an ephemeral wetland	2017	No relevant/useable data/fertility traits
TEMP828	HUBBS, C.; STRAWN, K.	THE EFFECTS OF LIGHT AND TEMPERATURE ON THE FECUNDITY OF THE GREENTHROAT DARTER, ETHEOSTOMA-LEPIDUM	1957	Missing data
TEMP852	Iguchi et al, 2015	Comparison of the effects of thermal stress and CO2-driven acidified seawater on fertilization in coral Acropora digitifera	2015	Seasonal data

TEMP883	Iwata, Y.et al.	Effect of low temperature on mating behavior of squid Loligo bleekeri	2008	No relevant/useable data/fertility traits
TEMP890	Jacobson, T.et al.	Combined effects of temperature and a pesticide on the Baltic amphipod Monoporeia affinis	2008	No relevant/useable data/fertility traits
TEMP905	Jeffs, A. G.et al.	Experimental effects of water temperature on the gametogenic development of broodstock in the oyster, Ostrea chilensis	2002	Missing data
TEMP917	Jin, S.et al. (a)	Optimizing reproductive performance and embryonic development of red swamp crayfish Procambarus clarkii by manipulating water temperature	2019	No relevant/useable data/fertility traits
TEMP924	Johnson, S.; Yund, P. m. j.	Remarkable longevity of dilute sperm in a free-spawning colonial ascidian	2004	Missing data
TEMP926	Johnston, C. E.et al.	MANIPULATION OF REPRODUCTIVE FUNCTION IN ATLANTIC SALMON (SALMO-SALAR) KELTS WITH CONTROLLED PHOTOPERIOD AND TEMPERATURE	1992	No relevant/useable data/fertility traits
TEMP927	Johnston, R. K.; Snell, T. W.	Moderately lower temperatures greatly extend the lifespan of Brachionus manjavacas (Rotifera): Thermodynamics or gene regulation?	2016	No relevant/useable data/fertility traits
TEMP933	Jonsson, B.et al.	Linking embryonic temperature with adult reproductive investment in Atlantic salmon Salmo salar	2014	No relevant/useable data/fertility traits
TEMP938	Joyce, A.et al.	EXPERIMENTAL EFFECTS OF TEMPERATURE AND PHOTOPERIOD ON SYNCHRONY OF GAMETOGENESIS AND SEX RATIO IN THE EUROPEAN OYSTER OSTREA EDULIS (LINNAEUS)	2003	No relevant/useable data/fertility traits
TEMP941	Juhel, G.et al.	A histological study of the gametogenic cycle of the freshwater mussel Dreissena polymorpha (Pallas, 1771) in Lough Derg, Ireland	2003	No relevant/useable data/fertility traits
TEMP945	Kalinda, C.et al.	Effect of temperature on the Bulinus globosus - Schistosoma haematobium system	2017	No relevant/useable data/fertility traits

OSM155	Kaviyarasan et al.	Population growth, nauplii production and post-embryonic development of Pseudodiaptomus annandalei (Sewell, 1919) in response to temperature, light intensity, pH, salinity and diets	2020	No relevant/useable data/fertility traits
TEMP985	Kelly, M. W.et al.	Trade-Offs, Geography, and Limits to Thermal Adaptation in a Tide Pool Copepod	2013	No useable temperature data
TEMP1022	Koger, C.et al.	Variations of light and temperature regimes and resulting effects on reproductive parameters in medaka (Oryzias latipes)	1999	Missing data
TEMP1071	Lagos, L.et al.	Assessment of the reproductive potential of the mussel (Mytilus chilensis) from two natural populations subjected to different conditioning temperatures	2012	No relevant/useable data/fertility traits
TEMP1072	Lahnsteiner, F. m. o.	Thermotolerance of brown trout, Salmo trutta, gametes and embryos to increased water temperatures	2012	No relevant/useable data/fertility traits
TEMP1076	Lahnsteiner, F.; Mansour, N.	The effect of temperature on sperm motility and enzymatic activity in brown trout Salmo trutta, burbot Lota lota and grayling Thymallus thymallus	2012	No relevant/useable data/fertility traits
TEMP1078	Lamont, M. M.; Fujisaki, I.	Effects of Ocean Temperature on Nesting Phenology and Fecundity of the Loggerhead Sea Turtle (Caretta caretta)	2014	No useable temperature data
HUM172	Lanna et al.	Environmental effects on the reproduction and fecundity of the introduced calcareous sponge Paraleucilla magna in Rio de Janeiro, Brazil	2015	Seasonal data
TEMP1095	Lazareva, V. I.et al.	Spatial distribution of plankton from the upper and Middle Volga reservoirs in years with different thermal conditions	2014	Missing data
OSM172	Le & Pham	Sperm Motilities in Waigieu Seaperch, Psammoperca waigiensis: Effects of Various Dilutions, pH, Temperature, Osmolality, and Cations	2017	Missing data
TEMP1119	Leicht, K.et al.	Inbreeding does not alter the response to an experimental heat wave in a freshwater snail	2019	Missing data
TEMP1124	Lesniak, T. C.et al.	Evaluating the Role of Temperature in the Reproduction of the Yellow Stingray (Urobatis jamaicensis)	2015	No relevant/useable data/fertility traits
TEMP1132	Li, C.et al. (a)	Influences of Temperature on Development and Survival, Reproduction and Growth of a Calanoid Copepod (Pseudodiaptomus dubia)	2009	No relevant/useable data/fertility traits

TEMP1152	Liberman, R.et al.	Octocoral Sexual Reproduction: Temporal Disparity Between Mesophotic and Shallow-Reef Populations	2018	No relevant/useable data/fertility traits
TEMP1167	Liu, S. L.et al.	Effect of high temperature stress on the fertility of male and female gametes of the sea cucumber Apostichopus japonicus	2016	No relevant/useable data/fertility traits
TEMP1175	Lloyd, S. S.et al.	Egg production by the copepod, Eurytemora affinis, in Chesapeake Bay turbidity maximum regions	2013	No relevant/useable data/fertility traits
TEMP1190	Lora-Vilchis, M.et al.	Histological characterization of the spawning process in the catarina scallop, Argopecten ventricosus (Sowerby II, 1842) induced by thermal shock and serotonin injection	2003	No relevant/useable data/fertility traits
TEMP1201	Luhring, T. M.et al.	Predators modify the temperature dependence of life-history trade-offs	2018	No relevant/useable data/fertility traits
TEMP1212	Lymbery, R. A.et al.	Post-ejaculation thermal stress causes changes to the RNA profile of sperm in an external fertilizer	2010	No relevant/useable data/fertility traits
TEMP1222	Maceda-Veiga, A.et al.	Chronic effects of temperature and nitrate pollution on Daphnia magna: Is this cladoceran suitable for widespread use as a tertiary treatment?	2015	No relevant/useable data/fertility traits
TEMP1225	Magalhaes, A. L.; Jacobi, C. M.	Colorful invasion in permissive Neotropical ecosystems: establishment of ornamental non-native poeciliids of the genera Poecilia/Xiphophorus (Cyprinodontiformes: Poeciliidae) and management alternatives	2017	No useable temperature data
TEMP1244	MANN, R. (a)	EFFECT OF TEMPERATURE ON GROWTH, PHYSIOLOGY, AND GAMETOGENESIS IN THE MANILA CLAM TAPES-PHILIPPINARUM (ADAMS AND REEVE, 1850)	1979	No relevant/useable data/fertility traits
TEMP1245	MANN, R. (b)	SOME BIOCHEMICAL AND PHYSIOLOGICAL ASPECTS OF GROWTH AND GAMETOGENESIS IN CRASSOSTREA-GIGAS AND OSTREA-EDULIS GROWN AT SUSTAINED ELEVATED-TEMPERATURES	1979	No relevant/useable data/fertility traits
TEMP1300	McCreesh, N.et al.	Effect of water temperature and population density on the population dynamics of Schistosoma mansoni intermediate host snails	2014	No relevant/useable data/fertility traits

TEMP1310	Meffe, 1991	LIFE-HISTORY CHANGES IN EASTERN MOSQUITOFISH (GAMBUSIA-HOLBROOKI) INDUCED BY THERMAL ELEVATION	1991	Missing data
TEMP1334	Migaud et al, 2002	Induction of out-of-season spawning in Eurasian perch Perca fluviatilis: effects of rates of cooling and cooling durations on female gametogenesis and spawning	2002	No useable temperature data
TEMP1339	Miller et al	Temperature is the evil twin: effects of increased temperature and ocean acidification on reproduction in a reef fish	2015	Missing data
TEMP1350	Mita et al	Effect of temperature on interactions between eggs and spermatozoa in four closely related species of sea urchins of the genus Echinometra	2007	Missing data
TEMP1375	MORAWSKA, B.	THE EFFECT OF WATER TEMPERATURE ELEVATION ON INCIPIENT AND CUMULATIVE FECUNDITY OF BATCH-SPAWNING TENCH, TINCA-TINCA (L	1984	Missing data
TEMP1389	Muck, J.et al.	Reproductive biology of the crayfish Orconectes luteus (Creaser) in a Missouri stream	2002	No useable temperature data
TEMP1398	Mura, G.; Zarattini, P. m. j.	Influence of parental rearing conditions on cyst production and hatching of Chirocephalus ruffoi, an endemic fairy shrimp from Italy (Anostraca	1999	No relevant/useable data/fertility traits
TEMP1406	Muthiga, N. A.	Testing for the effects of seasonal and lunar periodicity on the reproduction of the edible sea urchin Tripneustes gratilla (L) in Kenyan coral reef lagoons	2009	No useable temperature data
TEMP1407	Muthiga, N. A.et al.	The timing and reproductive output of the commercial sea cucumber Holothuria scabra on the Kenyan coast	2005	No useable temperature data
TEMP1419	Nakajima, J.; Onikura, N. m. j.	Life history of Pike Gudgeon, Pseudogobio esocinus (Cypriniformes, Cyprinidae): differences between the upper and lower reaches in a single river	2016	Missing data
TEMP1421	Nakamura, M.et al.	Sexual characteristics of high-temperature sterilized male Mozambique tilapia, Oreochromis mossambicus	2015	Missing data
OSM233	Nissling & Wallin	Recruitment variability in Baltic flounder (Platichthys solemdali) - effects of salinity with implications for stock development facing climate change	2020	No relevant/useable data/fertility traits
TEMP1505	Ortiz, N.et al.	The reproductive cycle of the red octopus Enteroctopus megalocyathus in fishing areas of Northern Patagonian coast	2011	No relevant/useable data/fertility traits

TEMP1516	Ozersky, T.et al.	Hot and sick? Impacts of warming and a parasite on the dominant zooplankter of Lake Baikal	2020	Missing data
TEMP1522	Palaima, A.; Spitze, K.	Is a jack-of-all-temperatures a master of none? An experimental test with Daphnia pulicaria (Crustacea : Cladocera)	2004	No relevant/useable data/fertility traits
TEMP1524	Palm, B. D.et al.	Seasonal variation in fecundity, egg case viability, gestation, and neonate size for little skates, Leucoraja erinacea, in the Gulf of Maine	2011	No relevant/useable data/fertility traits
TEMP1538	Papoulias, D. M.et al.	Characterization of environmental cues for initiation of reproductive cycling and spawning in shovelnose sturgeon Scaphirhynchus platorynchus in the Lower Missouri River, USA	2011	No relevant/useable data/fertility traits
TEMP1563	Paull, S. H.; Johnson, P. T. J. m. a.	High temperature enhances host pathology in a snail-trematode system: possible consequences of climate change for the emergence of disease	2011	Missing data
TEMP1574	Peñaranda al.	Temperature modulates testis steroidogenesis in European eel	2016	No relevant/useable data/fertility traits
TEMP1589	Peterson, W.et al.	Cross-shelf variation in calanoid copepod production during summer 1996 off the Oregon coast, USA	2002	No useable temperature data
TEMP1610	Pinto, R. L.et al.	Early release of eggs and embryos in a brooding ancient asexual ostracod: brood selection or a gambling strategy to increase fecundity?	2007	No relevant/useable data/fertility traits
TEMP1631	Potter, T.et al.	Exfoliation of epithelial cells by the scallop Placopecten magellanicus: Seasonal variation and the effects of elevated water temperatures	1997	No relevant/useable data/fertility traits
OSM264	Qiu & Qian	Combined effects of salinity and temperature on juvenile survival, growth and maturation in the polychaete Hydroides elegans	1998	No relevant/useable data/fertility traits
TEMP1674	Quintana, L.et al.	Temperature induces gonadal maturation and affects electrophysiological sexual maturity indicators in Brachyhypopomus pinnicaudatus from a temperate climate	2004	No relevant/useable data/fertility traits
TEMP1692	Ramos-Jiliberto, R.; Aranguiz-Acuna, A. m. n.	Between-species differences in demographic responses to temperature of coexisting cladocerans	2007	No relevant/useable data/fertility traits

OSM267	Rebolledo et al.	Combined effects of temperature and salinity on the demographic response of Proales similis (Beauchamp, 1907) and Brachionus plicatilis (Muller, 1786) (Rotifera) to mercury	2018	No relevant/useable data/fertility traits
TEMP1714	Refsnider, J. M.et al.	Population sex ratios under differing local climates in a reptile with environmental sex determination	2014	No relevant/useable data/fertility traits
TEMP1715	Rehage, J. S.et al.	A closer look at invasiveness and relatedness: life histories, temperature, and establishment success of four congeners	2020	No relevant/useable data/fertility traits
TEMP1722	Reyjol, Y.et al.	Effects of temperature on biological and biochemical indicators of the life-history strategy of bullhead Cottus gobio	2009	No relevant/useable data/fertility traits
TEMP1732	Rietzler, A. C.et al.	Predation and reproductive performance in two pelagic typhloplanid turbellarians	2018	Missing data
TEMP1753	Rogers, D. C.	Hatching response to temperature along a latitudinal gradient by the fairy shrimp Branchinecta lindahli (Crustacea; Branchiopoda; Anostraca) in culture conditions	2015	Missing data
TEMP1777	Rubenson, E. S.; Olden, J. D.	Spatiotemporal Spawning Patterns of Smallmouth Bass at Its Upstream Invasion Edge	2016	No useable temperature data
TEMP1824	Santerre, C.et al.	Oyster sex determination is influenced by temperature - First clues in spat during first gonadic differentiation and gametogenesis	2013	No relevant/useable data/fertility traits
TEMP1825	Santhanam, P.et al.	Effect of temperature and algal food on egg production and hatching of copepod, Paracalanus parvus	2013	Missing data
TEMP1846	Schaan et al, 2009	Reproductive biology of the Neotropical electric fish Brachyhypopomus draco (Teleostei: Hypopomidae) from southern Brazil	2009	Seasonal data
TEMP1850	Schlesinger et al, 2010	Sexual Plasticity and Self-Fertilization in the Sea Anemone Aiptasia diaphana	2010	No relevant/useable data/fertility traits
TEMP1861	Schwartz et al, 2016	Effects of fluctuating temperature and food availability on reproduction and lifespan	2016	No relevant/useable data/fertility traits
TEMP1879	Sewell and Young, 1999	Temperature limits to fertilization and early development in the tropical sea urchin Echinometra lucunter	1999	Missing data

TEMP1891	Shama	Bet hedging in a warming ocean: predictability of maternal environment shapes offspring size variation in marine sticklebacks	2015	Missing data
TEMP1917	Shpigel, M.et al.	The effect of photoperiod and temperature on the reproduction of European sea urchin Paracentrotus lividus	2004	Missing data
TEMP1943	Skurdal, J.et al.	Fluctuating fecundity parameters and reproductive investment in crayfish: driven by climate or chaos?	2011	No relevant/useable data/fertility traits
TEMP1968	SPIEGLER, M.; OPPENHEIMER, S. m. a.	EXTENDING THE VIABILITY OF SEA-URCHIN GAMETES	1995	No useable temperature data
TEMP1984	Steer, M. A.et al.	The role of temperature and maternal ration in embryo survival: using the dumpling squid Euprymna tasmanica as a model	2004	Missing data
TEMP2006	Sullivan, R. M.; Hileman, J. P.	Effects of managed flows on Chinook Salmon (Oncorhynchus tshawytscha) in relation to run-timing, fertility, and fluctuations in water temperature and flow volume	2019	No relevant/useable data/fertility traits
TEMP2011	Sun, D.; Niu, C. J.	Adaptive significance of temperature-induced egg size plasticity in a planktonic rotifer, Brachionus calyciflorus	2012	No relevant/useable data/fertility traits
TEMP2037	Taranger, G.et al.	Effects of photoperiod, temperature and GnRHa treatment on the reproductive physiology of Atlantic salmon (Salmo salar L.) broodstock	2003	Missing data
TEMP2045	Teaniniuraitemoana, V.et al.	Effect of temperature, food availability, and estradiol injection on gametogenesis and gender in the pearl oyster Pinctada margaritifera	2016	No relevant/useable data/fertility traits
TEMP2050	Tessema, M.et al.	Effect of rearing temperatures on the sex ratios of Oreochromis niloticus populations	2006	No relevant/useable data/fertility traits
TEMP2076	Tropea, C.et al.	Effect of Temperature on Biochemical Composition, Growth and Reproduction of the Ornamental Red Cherry Shrimp Neocaridina heteropoda heteropoda (Decapoda, Caridea)	2015	No relevant/useable data/fertility traits
TEMP2101	UYE, S.; SHIBUNO, N. m. m.	REPRODUCTIVE-BIOLOGY OF THE PLANKTONIC COPEPOD PARACALANUS SP IN THE INLAND SEA OF JAPAN	1992	Seasonal data

TEMP2124	Velazquez, A. m. d.	Reproductive strategies of the spiny lobster Panulirus interruptus related to the marine environmental variability off central Baja California, Mexico: management implications	2003	Seasonal data
TEMP2125	VELEZ, A.; EPIFANIO, C.	EFFECTS OF TEMPERATURE AND RATION ON GAMETOGENESIS AND GROWTH IN THE TROPICAL MUSSEL PERNA-PERNA (L	1981	No relevant/useable data/fertility traits
PH192	Vihtakari et al.	Effects of Ocean Acidification and Warming on Sperm Activity and Early Life Stages of the Mediterranean Mussel (Mytilus galloprovincialis)	2013	No relevant/useable data/fertility traits
TEMP2181	Wiklund, A. K. E.; Sundelin, B.	Impaired reproduction in the amphipods Monoporeia affinis and Pontoporeia femorata as a result of moderate hypoxia and increased temperature	2001	Missing data
TEMP2188	Wilson, R. m. d.	Temperature influences the coercive mating and swimming performance of male eastern mosquitofish	2005	No relevant/useable data/fertility traits
TEMP2189	Wilson, R. S.et al.	Competition moderates the benefits of thermal acclimation to reproductive performance in male eastern mosquitofish	2007	No relevant/useable data/fertility traits
TEMP2190	Wilson, R. S.et al.	Consequences of thermal acclimation for the mating behaviour and swimming performance of female mosquito fish	2007	No relevant/useable data/fertility traits
TEMP2195	Wiwatanaratanabutr et al	Impacts of temperature and crowding on sex ratio, fecundity and Wolbachia infection intensity in the copepod, Mesocyclops thermocyclopoides	2016	No relevant/useable data/fertility traits
OSM335	Wylie et al.	Effects of salinity and temperature on artificial cultivation and early ontogeny of giant kokopu, Galaxias argenteus (Gmelin 1789)	2016	Missing data
TEMP2263	Zak, J.; Reichard, M.	Fluctuating temperatures extend median lifespan, improve reproduction and reduce growth in turquoise killifish	2020	No relevant/useable data/fertility traits
TEMP2282	Zhang, G. T.et al.	Summer reproduction of the planktonic copepod Calanus sinicus in the Yellow Sea: influences of high surface temperature and cold bottom water	2007	No relevant/useable data/fertility traits

Supplementary Figure Legends

Supplementary Figure 1. PRISMA diagram showing the selection process for the studies included in this meta-analysis.

Supplementary Figure 2. A bar plot showing all the species included in this meta-analysis with the number of effect sizes per species indicated on the right of the x-axis, and the number of unique papers contributing to each species' data indicated on the left of the x-axis. These bars are color-coded according to the phylum of the species.

Supplementary Figure 3. Phylogenetic tree of the species represented in the thermal tolerance moderator model (n= 13, k=35).

Supplementary Figure 4. Alluvial plot showing the relationship between the development stage of animals exposed and their fertilisation mode. The width of the flows between the development stages and fertilisation modes is proportional to the count of effect sizes for each combination. Light red flows represent effect sizes from external fertilisers and the turquoise shows effect sizes from internal fertilisers.

Supplementary Figure 5. Alluvial plot showing the connection between the development stage of animals exposed and the sex of the trait studied. The thickness of the flows between the life stages and sex of traits is based on the number of effect sizes for each pairing. Blue flows represent male fertility traits, green represents female traits and red flows represent traits which are categorised as both.

Supplementary Figure 6. Alluvial plot showing the relationship between different trait categories and the sex of the traits measured. The flows between trait categories and the sex of traits vary in width based on the count of effect sizes for each combination. Blue flows represent male fertility traits, green represents female traits and red flows represent traits which are categorised as both.

Supplementary Figure 7. Meta-regression of the effect of type of temperature change on fertility for marine (*k*=1326, *n*=177), freshwater (*k*=473, *n*=88) and other aquatic animals (*k*=95, *n*=14). The small black bars show the 95% confidence intervals (CI), the longer thin bars show the 95% prediction intervals (PI), and the number in parentheses represents the number of studies. The dashed line indicates 0. The x-axis shows the magnitude of the effect size (r_b and *r)* and the size of each point showing a measure of the standard error. The vertical placement on the y-axis is arbitrary and varies based on the quantity of data points associated with a particular effect size value.

Supplementary Figure 8. Meta-regression showing the effect of the phylum of animal exposed on the estimates of the effect of temperature changes on the fertility of aquatic animals. The small black bars show the 95% confidence intervals (CI), and the longer thin bars show the 95% prediction intervals (PI), and the number in parentheses represents the number of studies. The dashed line indicates 0. The x-axis shows the magnitude of the effect size (r_b and *r)* and the size of each point showing a measure of the standard error. The vertical placement on the y-axis is arbitrary and varies based on the quantity of data points associated with a particular effect size value. Annelida *(k=43, n = 8),* Arthropoda *(k=393, n=86),* Chordata *(k=722, n=105),* Cnidaria *(k=44, n=13),* Echinodermata *(k=258, n=31),* Mollusca *(k=325, n=52),* Porifera *(k=48, n=4),* Rotifera *(k=60, n=10).* Ctenophora removed due to singular effect size (k=1, n=1).

Supplementary Figure 9. Meta-regression showing the interaction between the habitat type and fertilisation mode. The small black bars show the 95% confidence intervals (CI), and the longer thin bars show the 95% prediction intervals (PI), the number in parentheses represents the number of studies. The dashed line indicates 0. The x-axis shows the magnitude of the effect size (r_b and *r)* and the size of each point showing a measure of the standard error. Circle symbol represents the estimated effect size for freshwater species and squares represent estimated effect sizes marine species. The y-axis represents the fertilisation mode.

Supplementary Figure 10. Meta-regression showing the effect size estimates for the sex exposed to the temperature change for only the effect sizes where the fertility trait was a combined trait (both). The small black bars show the 95% confidence intervals (CI), and the longer thin bars show the 95% prediction intervals (PI), and the number in parentheses represents the number of studies. The dashed line indicates 0. The x-axis shows the magnitude of the effect size (r_b and *r)* and the size of each point showing a measure of the standard error. The vertical placement on the y-axis is arbitrary and varies based on the quantity of data points associated with a particular effect size value.

Supplementary Figure 11. Meta-regression showing the effect of whether the temperature changes measured were experimental (k=1635, n=228) or natural (k=238, n=58) on the estimates of the effect of temperature changes on the fertility of aquatic animals. The small black bars show the 95% confidence intervals (CI), and the longer thin bars show the 95% prediction intervals (PI), and the number in parentheses represents the number of studies. The dashed line indicates 0. The x-axis shows the magnitude of the effect size (r_b and *r)* and the size of each point showing a measure of the standard error. The vertical placement on the y-axis is arbitrary and varies based on the quantity of data points associated with a particular effect size value.

Supplementary Figure 12. Meta-regression showing the effect size estimates (*r_b* and *r)* for different durations of temperature change (days, *k*=1362, *n*=187). The x-axis shows the duration of the temperature change, and the size of each point shows the population size (N). The vertical placement on the y-axis shows the effect size estimate.

Supplementary Figure 13. Meta-regression showing the effect size estimates (*r_b* and *r)* for the effect of temperature on the fertility of aquatic animals and the square root of the inverse of the sample size. The x-axis shows the square root of the inverse of the sample size, and the size of each point shows the sample size of each effect size N. The slope (β) = -0.0026. The vertical placement on the y-axis shows the effect size estimate.

Supplementary Figure 14. Meta-regression showing the effect size estimates (*r_b* and *r)* for the effect of temperature on the fertility of aquatic animals over time. The x-axis shows the publication year, and the size of each point shows the sample size of each effect size N. The slope (β) = -0.0026. The vertical placement on the y-axis shows the effect size estimate.

References for studies included in this meta-analysis

Abdul Wahab, M. A., R. De Nys, N. Webster, and S. Whalan. 2014. Phenology of sexual reproduction in the common coral reef sponge, Carteriospongia foliascens. Coral Reefs.

Adriaenssens, B., R. van Damme, F. Seebacher, and R. S. Wilson. 2012. Sex cells in changing environments: can organisms adjust the physiological function of gametes to different temperatures? Global Change Biology 18:1797–1803.

Ahnesjo, I. 1995. Temperature affects male and female potential reproductive rates differently in the sex-role reversed pipefish, Syngnathus typhle. Behavioral Ecology 6:229–233.

Airi, V., F. Gizzi, G. Falini, O. Levy, Z. Dubinsky, and S. Goffredo. 2014. Reproductive Efficiency of a Mediterranean Endemic Zooxanthellate Coral Decreases with Increasing Temperature along a Wide Latitudinal Gradient. PLoS ONE 9:e91792.

Albright, R., and B. Mason. 2013. Projected Near-Future Levels of Temperature and pCO2 Reduce Coral Fertilization Success. PLoS ONE 8:e56468.

Anahi, B. A., M. C. Lopez-Abbate, F. Biancalana, and M. S. Hoffmeyer. 2014. Influence of Experimental Thermal Shifts and Overcrowding on Fecundity in Wild Females of Acartia Tonsa of the Bahía Blanca Estuary. Brazilian Journal of Oceanography 62:201–207.

Anderson, K., N. Pankhurst, H. King, and A. Elizur. 2017a. Effects of GnRHa treatment during vitellogenesis on the reproductive physiology of thermally challenged female Atlantic salmon (Salmo salar). PeerJ 5:e3898.

Anderson, K., N. Pankhurst, H. King, and A. Elizur. 2017b. Estrogen therapy offsets thermal impairment of vitellogenesis, but not zonagenesis, in maiden spawning female Atlantic salmon (Salmo salar). PeerJ 5:e3897.

Andronikov, V. B. 1975. Heat resistance of gametes of marine invertebrates in relation to temperature conditions under which the species exist. Marine Biology 30:1–11.

Arantes, F. P., H. B. Santos, E. Rizzo, Y. Sato, and N. Bazzoli. 2011. Influence of water temperature on induced reproduction by hypophysation, sex steroids concentrations and final oocyte maturation of the "curimatã-pacu" Prochilodus argenteus (Pisces: Prochilodontidae). General and Comparative Endocrinology 172:400–408.

Arendt, J., and D. Fairbairn. 2012. Reproductive allometry does not explain the temperature-size rule in water striders (Aquarius remigis). Evolutionary Ecology 26:745–757.

Arizmendi-Mejía, R., J.-B. Ledoux, S. Civit, A. Antunes, Z. Thanopoulou, J. Garrabou, and C. Linares. 2015. Demographic responses to warming: reproductive maturity and sex influence vulnerability in an octocoral. Coral Reefs 34:1207–1216.

Armstrong, E. J., V. Dubousquet, S. C. Mills, and J. H. Stillman. 2020. Elevated temperature, but not acidification, reduces fertilization success in the small giant clam, Tridacna maxima. Marine Biology 167:8.

Athibai, S., and L. Sanoamuang. 2008. Effect of Temperature on Fecundity, Life Span and Morphology of Long- and Short-Spined Clones of Brachionus caudatus f. apsteini (Rotifera). International Review of Hydrobiology 93:690–699.

Atse, C. B., C. Audet, and J. De La Noüe. 2002. Effects of temperature and salinity on the reproductive success of Arctic charr, Salvelinus alpinus (L.): egg composition, milt characteristics and fry survival. Aquaculture Research 33:299–309.

Awaïss, A., and P. Kestemont. 1992. An investigation into the mass production of the freshwater rotifer Brachionus calyciflorus Pallas. 2. Influence of temperature on the population dynamics. Aquaculture 105:337–344.

Baldanzi, S., C. D. McQuaid, and F. Porri. 2015. Temperature Effects on Reproductive Allocation in the Sandhopper Talorchestia capensis. The Biological Bulletin 228:181–191.

Ban, S. 2000. In situ egg production and hatching success of the marine copepod Pseudocalanus newmani in Funka Bay and adjacent waters off southwestern Hokkaido, Japan: associated to diatom bloom. Journal of Plankton Research 22:907–922.

Bautista, B., R. P. Harris, V. Rodriguez, and F. Guerrero. 1994. Temporal variability in copepod fecundity during two different spring bloom periods in coastal waters off Plymouth (SW England). Journal of Plankton Research 16:1367–1377.

Bednarska, A., J. ŁOŚ, and P. DAWIDOWICZ. 2011. Temperature-dependent effect of filamentous cyanobacteria on Daphnia magna life history traits. Journal of Limnology 70:353–358.

Bednarska, A., and M. Slusarczyk. 2013. Effect of non-toxic, filamentous cyanobacteria on egg abortion in Daphnia under various thermal conditions. Hydrobiologia 715:151–157.

Beirão, J., C. Purchase, B. Wringe, and I. Fleming. 2014. Sperm plasticity to seawater temperatures in Atlantic cod Gadus morhua is affected more by population origin than individual environmental exposure. Marine Ecology Progress Series 495:263–274.

Berger. (n.d.). Reproduction of the intertidal barnacle Balanus glandula along an estuarine gradient - Berger - 2009 - Marine Ecology - Wiley Online Library. https://onlinelibrary.wiley.com/doi/epdf/10.1111/j.1439-0485.2009.00280.x.

Berger, M. S. 2009. Reproduction of the intertidal barnacle Balanus glandula along an estuarine gradient. Marine Ecology 30:346–353.

Betini, G. S., X. Wang, T. Avgar, M. M. Guzzo, and J. M. Fryxell. 2020. Food availability modulates temperature-dependent effects on growth, reproduction, and survival in Daphnia magna. Ecology and Evolution 10:756–762.

Beyer, S., S. Alonzo, and S. Sogard. 2021. Zero, one or more broods: reproductive plasticity in response to temperature, food, and body size in the live-bearing rosy rockfish Sebastes rosaceus. Marine Ecology Progress Series 669:151–173.

Beyrend-Dur, D., R. Kumar, T. R. Rao, S. Souissi, S.-H. Cheng, and J.-S. Hwang. 2011. Demographic parameters of adults of Pseudodiaptomus annandalei (Copepoda: Calanoida): Temperature–salinity and generation effects. Journal of Experimental Marine Biology and Ecology 404:1–14.

Binet, M. T., and C. J. Doyle. 2013. Effect of near-future seawater temperature rises on sea urchin sperm longevity. Marine and Freshwater Research 64:1.

Boavida-Portugal, J., A. Moreno, L. Gordo, and J. Pereira. 2010. Environmentally adjusted reproductive strategies in females of the commercially exploited common squid Loligo vulgaris. Fisheries Research 106:193–198.

Bombardelli, R. A., E. A. Sanches, D. M. Baggio, R. M. Sykora, B. E. D. Souza, L. Tessaro, and P. A. Piana. 2013. Effects of the spermatozoa: oocyte ratio, water volume and water temperature on artificial fertilization and sperm activation of cascudo-preto. Revista Brasileira de Zootecnia 42:1–6.

Bonacina, C., A. Pasteris, G. Bonomi, and D. Marzuoli. 1994. Quantitative observations on the population ecology of Branchiura sowerbyi (Oligochaeta, Tubificidae). Hydrobiologia 278:267–274.

Boni, R., A. Gallo, M. Montanino, A. Macina, and E. Tosti. 2016. Dynamic changes in the sperm quality of Mytilus galloprovincialis under continuous thermal stress. Molecular Reproduction and Development 83:162–173.

Borg, B. 1982. Seasonal effects of photoperiod and temperature on spermatogenesis and male secondary sexual characters in the three-spined stickleback, Gasterosteus aculeatus L. Canadian Journal of Zoology 60:3377–3386.

Bouquet, J. M., C. Troedsson, A. Novac, M. Reeve, A. K. Lechtenborger, W. Massart, K. S. Skaar, A. Aasjord, S. Dupont, and E. M. Thompson.Increased fitness of a key appendicularian zooplankton species under warmer, acidified seawater conditions. Plos One 13.

Brambila-Souza, G., C. C. Mylonas, P. H. Mello, R. Y. Kuradomi, S. R. Batlouni, C. E. Tolussi, and R. G. Moreira. 2019. Thermal manipulation and GnRHa therapy applied to the reproduction of lambari-do-rabo-amarelo, Astyanax altiparanae females (Characiformes: Characidae) during the non-breeding season. General and Comparative Endocrinology 279:120–128.

Breckels, R. D., and B. D. Neff. 2013. The effects of elevated temperature on the sexual traits, immunology and survivorship of a tropical ectotherm. Journal of Experimental Biology:jeb.084962.

Breckels, R. D., and B. D. Neff. 2014. Rapid evolution of sperm length in response to increased temperature in an ectothermic fish. Evolutionary Ecology 28:521–533.

Brown, M. D., B. H. Kay, and J. K. Hendrikz. 1991. Evaluation of Australian Mesocyclops (Cyclopoida: Cyclopidae) for Mosquito Control. Journal of Medical Entomology 28:618–623.

Brown, N. P., R. J. Shields, and N. R. Bromage. 2006. The influence of water temperature on spawning patterns and egg quality in the Atlantic halibut (Hippoglossus hippoglossus L.). Aquaculture 261:993–1002.

Brugnano, C., L. Guglielmo, A. Ianora, and G. Zagami. 2009. Temperature effects on fecundity, development and survival of the benthopelagic calanoid copepod, Pseudocyclops xiphophorus. Marine Biology 156:331–340.

Bunioto, T. C., and M. S. Arcifa. 2007. Effects of food limitation and temperature on cladocerans from a tropical Brazilian lake. Aquatic Ecology 41:569–578.

Burdloff, D., S. Gasparini, F. Villate, I. Uriarte, U. Cotano, B. Sautour, and H. Etcheber. 2002. Egg production of the copepod Acartia bifilosa in two contrasting European estuaries in relation to seston composition. Journal of Experimental Marine Biology and Ecology 274:1–17.

Burris, Z. P., and H. G. Dam. 2015. Female mating status affects mating and male mate-choice in the copepod genus Acartia. Journal of Plankton Research 37:183–196.

Butler, M. I., and C. W. Burns. 1991. The influence of temperature and resource level on the fecundity of a predatory planktonic mite, Piona exigua Viets. Oecologia 88:220–227.

Bylenga, C., V. Cummings, and K. Ryan. 2015. Fertilisation and larval development in an Antarctic bivalve, Laternula elliptica, under reduced pH and elevated temperatures. Marine Ecology Progress Series 536:187–201.

Byrne, M., M. Gonzalez-Bernat, S. Doo, S. Foo, N. Soars, and M. Lamare. 2013. Effects of ocean warming and acidification on embryos and non-calcifying larvae of the invasive sea star Patiriella regularis. Marine Ecology Progress Series 473:235–246.

Byrne, M., N. A. Soars, M. A. Ho, E. Wong, D. McElroy, P. Selvakumaraswamy, S. A. Dworjanyn, and A. R. month = sep Davis. 2010a. Fertilization in a suite of coastal marine invertebrates from {SE} {Australia} is robust to near-future ocean warming and acidification. MARINE BIOLOGY 157:2061–2069.

Byrne, M., N. Soars, P. Selvakumaraswamy, S. A. Dworjanyn, and A. R. Davis. 2010b. Sea urchin fertilization in a warm, acidified and high pCO2 ocean across a range of sperm densities. Marine Environmental Research 69:234–239.

Caballes, C., M. Pratchett, M. Raymundo, and J. Rivera-Posada. 2017. Environmental Tipping Points for Sperm Motility, Fertilization, and Embryonic Development in the Crown-of-Thorns Starfish. Diversity 9:10.

Calbet, A., and M. Alcaraz. 1996. Effects of constant and fluctuating food supply on egg production rates of Acartia grani (Copepoda:Calanoida). Marine Ecology Progress Series 140:33–39.

Caldwell, G. S., S. Fitzer, C. S. Gillespie, G. Pickavance, E. Turnbull, and M. G. Bentley. 2011. Ocean acidification takes sperm back in time. Invertebrate Reproduction & Development 55:217–221.

Camargo-Cely, A., and R. Collin. 2019. Combined effects of temperature, salinity, and diet simulating upwelling and nonupwelling seasons alter life-history characteristics of a tropical invertebrate. Ecology and Evolution 9:14368–14378.

Campbell, R. W., and E. J. Head. 2000. Egg production rates of Calanus finmarchicus in the western North Atlantic: effect of gonad maturity, female size, chlorophyll concentration, and temperature. Canadian Journal of Fisheries and Aquatic Sciences 57:518–529.

Cardoso, P. G., E. M. Loganimoce, T. Neuparth, M. J. Rocha, E. Rocha, and F. Arenas. 2018. Interactive effects of increased temperature, pCO2 and the synthetic progestin levonorgestrel on the fitness and breeding of the amphipod Gammarus locusta. Environmental Pollution 236:937–947.

Carlotti, F., C. Rey, A. Javanshir, and S. Nival. 1997. Laboratory studies on egg and faecal pellet production of Centropages typicus : effect of age, effect of temperature, individual variability. Journal of Plankton Research 19:1143–1165.

Castellani, C., and Y. Altunbas. 2006. Factors controlling the temporal dynamics of egg production in the copepod Temora longicornis. Marine Ecology Progress Series 308:143–153.

Castro, J. S., S. Braz-Mota, D. F. Campos, S. S. Souza, and A. L. Val. 2020. High Temperature, pH, and Hypoxia Cause Oxidative Stress and Impair the Spermatic Performance of the Amazon Fish Colossoma macropomum. Frontiers in Physiology 11:772.

Castro-Longoria, E. 1999. The production of subitaneous and diapause eggs: a reproductive strategy for Acartia bifilosa (Copepods: Calanoida) in Southampton Water, UK. Journal of Plankton Research 21:65–84.

Cavalheri, H. B., C. C. Symons, M. Schulhof, N. T. Jones, and J. B. Shurin. 2019. Rapid evolution of thermal plasticity in mountain lake Daphnia populations. Oikos 128:692–700.

Cejko, B. I., S. Judycka, and R. Kujawa. 2016. The effect of different ambient temperatures on river lamprey (Lampetrafluviatilis) egg and sperm production under controlled conditions. Journal of Thermal Biology 62:70–75.

Cha, J.-H., D. Martin, and M. Bhaud. 1997. Effects of temperature on oocyte growth in the Mediterranean terebellid Eupolymnia nuebulosa (Annelida: Polychaeta). Marine Biology 128:433–439.

Chang, C.-F., H.-J. Hu, and H.-C. Tang. 1992. Effects of photoperiod and temperature on testicular development in male ayu,Plecoglossus altivelis. Environmental Biology of Fishes 34:309–314.

Chase, M. E., and M. L. H. Thomas. 1995. The effect of the rate and onset of temperature increase on spawning of the periwinkle, Littorina littorea (L.). Journal of Experimental Marine Biology and Ecology 186:277–287.

Chávez-Villalba, J., J. Pommier, J. Andriamiseza, S. Pouvreau, J. Barret, J.-C. Cochard, and M. Le Pennec. 2002. Broodstock conditioning of the oyster Crassostrea gigas: origin and temperature effect. Aquaculture 214:115–130.

Chirgwin, E., D. J. Marshall, and K. Monro. 2020. Physical and physiological impacts of ocean warming alter phenotypic selection on sperm morphology. Functional Ecology 34:646–657.

Chow-Fraser, P., and E. J. Maly. 1991. Factors Governing Clutch Size in Two Species of Diaptomus (Copepoda: Calanoida). Canadian Journal of Fisheries and Aquatic Sciences 48:364–370.

Clark, R. W., A. Henderson-Arzapalo, and C. V. Sullivan. 2005. Disparate effects of constant and annually-cycling daylength and water temperature on reproductive maturation of striped bass (Morone saxatilis). Aquaculture 249:497–513.

Clotteau, G., and F. Dubé. 1993. Optimization of fertilization parameters for rearing surf clams (Spisula solidissima). Aquaculture 114:339–353.

Collin, R. 2012. Temperature-mediated trade-offs and changes in life-history integration in two slipper limpets (Gastropoda: Calyptraeidae) with planktotrophic development: TEMPERATURE-MEDIATED TRADE-OFFS. Biological Journal of the Linnean Society 106:763–775.

Collin, R., and A. Spangler. 2012. Impacts of Adelphophagic Development on Variation in Offspring Size, Duration of Development, and Temperature-Mediated Plasticity. The Biological Bulletin 223:268–277.

Cuthbert, R. N., J. T. A. Dick, A. Callaghan, and J. W. E. Dickey. 2018. Biological control agent selection under environmental change using functional responses, abundances and fecundities; the Relative Control Potential (RCP) metric. Biological Control 121:50–57.

Dadras, H., V. Dzyuba, A. Golpour, M. Xin, and B. Dzyuba. 2019. In vitro antioxidant enzyme activity and sperm motility at different temperatures in sterlet Acipenser ruthenus and rainbow trout Oncorhynchus mykiss. Fish Physiology and Biochemistry 45:1791–1800.

Dadras, H., S. Sampels, A. Golpour, V. Dzyuba, J. Cosson, and B. Dzyuba. 2017. Analysis of common carp Cyprinus carpio sperm motility and lipid composition using different in vitro temperatures. Animal Reproduction Science 180:37–43.

De Lapeyre, B. A., A. Müller-Belecke, and G. Hörstgen-Schwark. 2009. Control of spawning activity in female Nile tilapia (Oreochromis niloticus) (L.) by temperature manipulation. Aquaculture Research 40:1031–1036.

De Putron, S. J., and J. S. Ryland. 2009. Effect of seawater temperature on reproductive seasonality and fecundity of Pseudoplexaura porosa (Cnidaria: Octocorallia): latitudinal variation in Caribbean gorgonian reproduction. Invertebrate Biology 128:213–222.

Delgado, M., and A. Pérez-Camacho. 2007. Comparative study of gonadal development of Ruditapes philippinarum (Adams and Reeve) and Ruditapes decussatus (L.) (Mollusca: Bivalvia): Influence of temperature. Scientia Marina 71:471–484.

Díaz-Martínez, J. P., E. D. J. Carpizo-Ituarte, and F. Benítez-Villalobos. 2019. Reproductive patterns of the black starry sea urchin Arbacia stellata in Punta Banda, Baja California, Mexico. Journal of the Marine Biological Association of the United Kingdom 99:1379–1391.

Dieterman, D. J., T. S. Walker, P. A. Cochran, and M. Konsti. 2016. Reproductive Traits of Brown Trout in Two Contrasting Streams of Southeast Minnesota. North American Journal of Fisheries Management 36:465–476.

Doan, N. X., M. T. T. Vu, H. Q. Pham, M. S. Wisz, T. G. Nielsen, and K. V. Dinh. 2019. Extreme temperature impairs growth and productivity in a common tropical marine copepod. Scientific Reports 9:4550.

Donelson, J., P. Munday, M. McCormick, N. Pankhurst, and P. Pankhurst. 2010. Effects of elevated water temperature and food availability on the reproductive performance of a coral reef fish. Marine Ecology Progress Series 401:233–243.

Dorts, J., G. Grenouillet, J. Douxfils, S. N. M. Mandiki, S. Milla, F. Silvestre, and P. Kestemont. 2012. Evidence that elevated water temperature affects the reproductive physiology of the European bullhead Cottus gobio. Fish Physiology and Biochemistry 38:389–399.

Doums, C., M.-A. Perdieu, and P. Jarne. 1998. Resource Allocation and Stressful Conditions in the Aphallic Snail Bulinus truncatus. Ecology 79:720.

Dumorné, K., I. Valdebenito, P. Contreras, P. U. Rodríguez, J. Risopatron, E. Figueroa, M. L. Estevez, R. Díaz, and J. Farías. 2018. Effect of pH, osmolality and temperature on sperm motility of pink cusk-eel (Genypterus blacodes, (Forster, 1801)). Aquaculture Reports 11:42–46.

Dzyuba, V., J. Cosson, M. Papadaki, C. C. Mylonas, C. Steinbach, M. Rodina, V. Tučkova, O. Linhart, W. L. Shelton, D. Gela, S. Boryshpolets, and B. Dzyuba. 2021. Influence of Environmental Temperature and Hormonal Stimulation on the In Vitro Sperm Maturation in Sterlet Acipenser ruthenus in Advance of the Spawning Season. Animals 11:1417.

Eads, A. R., J. P. Evans, and W. J. Kennington. 2016. Plasticity of fertilization rates under varying temperature in the broadcast spawning mussel, Mytilus galloprovincialis. Ecology and Evolution 6:6578–6585.

Eads, A. R., W. J. Kennington, and J. P. Evans.Interactive effects of ocean warming and acidification on sperm motility and fertilization in the mussel Mytilus galloprovincialis. Marine Ecology Progress Series 562:101–111.

Ebert, E., and R. Hamilton. 1983. Ova fertility relative to temperature and to the time of gamete mixing in the red abalone, Haliotis rufescens. California Fish and Game 69:115–120.

Effer, B., E. Figueroa, A. Augsburger, and I. Valdebenito. 2013. Sperm biology of Merluccius australis: Sperm structure, semen characteristics and effects of pH, temperature and osmolality on sperm motility. Aquaculture 408–409:147–151.

Epherra, L., D. G. Gil, T. Rubilar, S. Perez-Gallo, M. B. Reartes, and J. A. Tolosano. 2015. Temporal and spatial differences in the reproductive biology of the sea urchin Arbacia dufresnii. Marine and Freshwater Research 66:329.

Ericson, J. A., M. A. Ho, A. Miskelly, C. K. King, P. Virtue, B. Tilbrook, and M. Byrne. 2012. Combined effects of two ocean change stressors, warming and acidification, on fertilization and early development of the Antarctic echinoid Sterechinus neumayeri. Polar Biology 35:1027–1034.

Escribano, R., C. Irribarren, and L. Rodriguez. 1996. Temperature and female size effects on egg production of Calanus chilensis: laboratory observations. Rev. Chil. Hist. Nat 69:365–377.

Ettinger-Epstein, P., S. W. Whalan, C. N. Battershill, and R. De Nys. 2007. Temperature cues gametogenesis and larval release in a tropical sponge. Marine Biology 153:171–178.

Fearman, J., and N. A. Moltschaniwskyj. 2010. Warmer temperatures reduce rates of gametogenesis in temperate mussels, Mytilus galloprovincialis. Aquaculture 305:20–25.

Feiner, Z. S., D. P. Coulter, S. C. Guffey, and T. O. Höök. 2016. Does overwinter temperature affect maternal body composition and egg traits in yellow perch Perca flavescens ? Journal of Fish Biology 88:1524–1543.

Fenkes, M., J. L. Fitzpatrick, K. Ozolina, H. A. Shiels, and R. L. Nudds. 2017. Sperm in hot water: Direct and indirect thermal challenges interact to impact on brown trout sperm quality. Journal of Experimental Biology:jeb.156018.

Fenkes, M., J. L. Fitzpatrick, H. A. Shiels, and R. L. Nudds. 2019. Acclimation temperature changes spermatozoa flagella length relative to head size in brown trout. Biology Open:bio.039461.

Fileto, C., M. S. Arcifa, R. Henry, and R. A. R. Ferreira. 2010. Effects of temperature, sestonic algae features, and seston mineral content on cladocerans of a tropical lake. Annales de Limnologie - International Journal of Limnology 46:135–147.

Fischer, S., and S. Thatje. 2008. Temperature-induced oviposition in the brachyuran crab Cancer setosus along a latitudinal cline: Aquaria experiments and analysis of field-data. Journal of Experimental Marine Biology and Ecology 357:157–164.

Fraile, B., F. J. Sáez, C. A. Vicentini, A. González, M. P. De Miguel, R. Paniagua, F. J. Saez, A. Gonzalez, and M. P. De Miguel. 1994. Effects of Temperature and Photoperiod on the Gambusia affinis holbrooki Testis during the Spermatogenesis Period. Copeia 1994:216.

Fraser, C. M. L., and B. K. K. Chan. 2019. Too hot for sex: mating behaviour and fitness in the intertidal barnacle Fistulobalanus albicostatus under extreme heat stress. Marine Ecology Progress Series 610:99–108.

Funamoto, T., and I. Aoki. 2002. Reproductive ecology of Japanese anchovy off the Pacific coast of eastern Honshu, Japan. Journal of Fish Biology 60:154–169.

Fuxjäger, L., S. Wanzenböck, E. Ringler, K. M. Wegner, H. Ahnelt, and L. N. S. Shama. 2019. Within-generation and transgenerational plasticity of mate choice in oceanic stickleback under climate change. Philosophical Transactions of the Royal Society B: Biological Sciences 374:20180183.

Galbraith, H. S., and C. C. Vaughn. 2009. Temperature and food interact to influence gamete development in freshwater mussels. Hydrobiologia 636:35–47.

Gallego, V., I. Mazzeo, M. C. Vílchez, D. S. Peñaranda, P. C. F. Carneiro, L. Pérez, and J. F. Asturiano. 2012. Study of the effects of thermal regime and alternative hormonal treatments on the reproductive performance of European eel males (Anguilla anguilla) during induced sexual maturation. Aquaculture 354–355:7–16.

Gallego, V., M. C. Vílchez, D. S. Peñaranda, L. Pérez, M. P. Herráez, J. F. Asturiano, and F. Martínez-Pastor. 2015. Subpopulation pattern of eel spermatozoa is affected by post-activation time, hormonal treatment and the thermal regimen. Reproduction, Fertility and Development 27:529.

Garrido, C. L., and B. J. Barber. 2001. Effects of temperature and food ration on gonad growth and oogenesis of the green sea urchin, Strongylocentrotus droebachiensis. Marine Biology 138:447–456.

Gharibi, M., M. Nematollahi, N. Agh, and B. Atashbar. 2016. Life cycle of the fairy shrimp, Phallocryptus spinosa Milne Edwards, 1840 (Crustacea: Anostraca) at different temperatures. Journal of Agricultural Science and Technology 18:171–177.

Gillet, C. 1991. Egg production in an Arctic charr (Salvelinus alpinus L.) brood stock: effects of temperature on the timing of spawning and the quality of eggs. Aquatic Living Resources 4:109–116.

Gislason, A. 2005. Seasonal and spatial variability in egg production and biomass of Calanus finmarchicus around Iceland. Marine Ecology Progress Series 286:177–192.

Gorokhova, E. 2003. Relationships between nucleic acid levels and egg production rates in Acartia bifilosa: implications for growth assessment of copepods in situ. Marine Ecology Progress Series 262:163–172.

Gouda, H., and Y. Agatsuma. 2020. Effect of high temperature on gametogenesis of the sea urchin Strongylocentrotus intermedius in the Sea of Japan, northern Hokkaido, Japan. Journal of Experimental Marine Biology and Ecology 525:151324.

GRUBERT, M. A., and A. J. RITAR. 2004. Temperature effects on the dynamics of gonad and oocyte development in captive wild-caught blacklip (Haliotis rubra) and greenlip (H. laevigata) abalone. Invertebrate Reproduction & Development 45:185–196.

Grubert, M. A., and A. J. Ritar. 2005. The effect of temperature and conditioning interval on the spawning success of wild-caught blacklip (Haliotis rubra, Leach 1814) and greenlip (H. laevigata, Donovan 1808) abalone. Aquaculture Research 36:654–665.

Hakalahti, T., H. Häkkinen, and E. T. Valtonen. 2004. Ectoparasitic Argulus coregoni (Crustacea: Branchiura) hedge their bets – studies on egg hatching dynamics. Oikos 107:295–302.

Hall, C. J. 2001. Effects of Salinity and Temperature on Survival and Reproduction of Boeckella hamata (Copepoda: Calanoida) from a Periodically Brackish Lake. Journal of Plankton Research 23:97–104.

Hall, C. J., and C. W. Burns. 2002. Effects of Temperature and Salinity on the Survival and Egg Production of Gladioferens pectinatus Brady (Copepodas: Calanoida). Estuarine, Coastal and Shelf Science 55:557–564.

Han, J., M.-C. Lee, J. C. Park, S. Kim, and J.-S. Lee. 2018. Effects of temperature shifts on life parameters and expression of fatty acid synthesis and heat shock protein genes in temperate and Antarctic copepods Tigriopus japonicus and Tigriopus kingsejongensis. Polar Biology 41:2459–2466.

Hermelink, B., S. Wuertz, B. Rennert, W. Kloas, and C. Schulz. 2013. Temperature control of pikeperch (Sander lucioperca) maturation in recirculating aquaculture systems—induction of puberty and course of gametogenesis. Aquaculture 400–401:36–45.

Hernáez, P., S. Palma, and I. S. Wehrtmann. 2008. Egg production of the burrowing shrimp Callichirus seilacheri (Bott 1955) (Decapoda, Callianassidae) in northern Chile. Helgoland Marine Research 62:351–356.

Herrera, M., I. Hachero-Cruzado, S. García, J. M. Mancera, and J. I. Navas. 2011. Reproduction of the wedge sole (Dicologoglossa cuneata Moreau) in captivity: spawning parameters and influence of the natural temperature. Reviews in Fish Biology and Fisheries 21:517–526.

Hirche, H.-J., U. Meyer, and B. Niehoff. 1997. Egg production of Calanus finmarchicus : effect of temperature, food and season. Marine Biology 127:609–620.

Ho, M. A., C. Price, C. K. King, P. Virtue, and M. Byrne. 2013. Effects of ocean warming and acidification on fertilization in the Antarctic echinoid Sterechinus neumayeri across a range of sperm concentrations. Marine Environmental Research 90:136–141.

Hoang, T., S. Y. Lee, C. P. Keenan, and G. E. Marsden. 2002. Maturation and spawning performance of pond-reared Penaeus merguiensis in different combinations of temperature, light intensity and photoperiod: Ovarian maturation of pond-reared P. merguiensis. Aquaculture Research 33:1243–1252.

Hoang, T., S. Y. Lee, C. P. Keenan, and G. E. Marsden. 2003. Improved reproductive readiness of pond-reared broodstock Penaeus merguiensis by environmental manipulation. Aquaculture 221:523–534.

Honkoop, P., J. Van Der Meer, J. Beukema, and D. Kwast. 1998. Does temperature-influenced egg production predict the recruitment in the bivalve Macoma balthica? Marine Ecology Progress Series 164:229–235.

Huang, X., P. Zhuang, L. Zhang, Z. Qiao, Q. Jiang, J. Liu, Z. Yao, and G. Feng. 2011. Effects of extracellular environment factors on the motility in Japanese eel spermatozoa. Cell Biology International 35:505–508.

Hyne, R. V., S. A. Gale, and C. K. King. 2005. Laboratory culture and life-cycle experiments with the benthic amphipod melita plumulosa (zeidler). Environmental Toxicology and Chemistry 24:2065–2073.

Ianora, A., M. G. Mazzocchi, and R. Grottoli. 1992. Seasonal fluctuations in fecundity and hatching success in the planktonic copepod Centropages typicus. Journal of Plankton Research 14:1483–1494.

Iglesias-Carrasco, M., L. Harrison, M. D. Jennions, and M. L. Head. 2020. Combined effects of rearing and testing temperatures on sperm traits. Journal of Evolutionary Biology 33:1715–1724.

Iguchi, K. 2012. Larger eggs at lower water temperature as a measure to assure effective hatchling size in the landlocked form of Ayu, Plecoglossus altivelis. Ichthyological Research 59:20–25.

Inoda, T., F. Tajima, H. Taniguchi, M. Saeki, K. Numakura, M. Hasegawa, and S. Kamimura. 2007. Temperature-Dependent Regulation of Reproduction in the Diving Beetle Dytiscus sharpi (Coleoptera: Dytiscidae). Zoological Science 24:1115–1121.

Ismail, H. N., J. G. Qin, and L. Seuront. 2011. Regulation of life history in the brackish cladoceran, Daphniopsis australis (Sergeev and Williams, 1985) by temperature and salinity. Journal of Plankton Research 33:763–777.

Jarrold, M. D., L. J. Chakravarti, E. M. Gibbin, F. Christen, G. Massamba-N'Siala, P. U. Blier, and P. Calosi. 2019. Life-history trade-offs and limitations associated with phenotypic adaptation under future ocean warming and elevated salinity. Philosophical Transactions of the Royal Society B: Biological Sciences 374:20180428.

Jigyasu, H. V., and V. K. Singh. 2010. Effect of environmental factors on the fecundity, hatchability and survival of snail Lymnaea (Radix) acuminata (Lamarck): vector of fascioliasis. Journal of Water and Health 8:109–115.

Jiménez-Melero, R., B. Santer, and F. Guerrero. 2005. Embryonic and naupliar development of Eudiaptomus gracilis and Eudiaptomus graciloides at different temperatures: comments on individual variability. Journal of Plankton Research 27:1175–1187.

Johnstone, J., S. Nash, E. Hernandez, and M. S. Rahman. 2019. Effects of elevated temperature on gonadal functions, cellular apoptosis, and oxidative stress in Atlantic sea urchin Arbacia punculata. Marine Environmental Research 149:40–49.

Kauler, P., and H. E. Enesco. 2011. The effect of temperature on life history parameters and cost of reproduction in the rotifer Brachionus calyciflorus. Journal of Freshwater Ecology 26:399–408.

Kelly, M. S. 2001. Environmental parameters controlling gametogenesis in the echinoid Psammechinus miliaris. Journal of Experimental Marine Biology and Ecology 266:67–80.

Kelly, M. W., M. B. DeBiasse, V. A. Villela, H. L. Roberts, and C. F. Cecola. 2016. Adaptation to climate change: trade-offs among responses to multiple stressors in an intertidal crustacean. Evolutionary Applications 9:1147–1155.

Kikko, T., T. Usuki, D. Ishizaki, Y. Kai, and Y. Fujioka. 2015. Relationship of egg and hatchling size to incubation temperature in the multiple-spawning fish Gnathopogon caerulescens (Honmoroko). Environmental Biology of Fishes 98:1151–1161.

Kim, S.-Y., N. B. Metcalfe, A. Da Silva, and A. Velando. 2017. Thermal conditions during early life influence seasonal maternal strategies in the three-spined stickleback. BMC Ecology 17:34.

King, H. R., and N. W. Pankhurst. 2004. Effect of short-term temperature reduction on ovulation and LHRHa responsiveness in female Atlantic salmon (Salmo salar) maintained at elevated water temperatures. Aquaculture 238:421–436.

King, H. R., N. W. Pankhurst, and M. Watts. 2007. Reproductive sensitivity to elevated water temperatures in female Atlantic salmon is heightened at certain stages of vitellogenesis. Journal of Fish Biology 70:190–205.

King, H. R., N. W. Pankhurst, M. Watts, and P. M. Pankhurst. 2003. Effect of elevated summer temperatures on gonadal steroid production, vitellogenesis and egg quality in female Atlantic salmon. Journal of Fish Biology 63:153–167.

Kokita, T. 2003. Potential latitudinal variation in egg size and number of a geographically widespread reef fish, revealed by common-environment experiments. Marine Biology 143:593–601.

Kokita, T. 2004. Latitudinal compensation in female reproductive rate of a geographically widespread reef fish. Environmental Biology of Fishes 71:213–224.

Kordbacheh, A., A. N. Shapiro, and E. J. Walsh. 2019. Reproductive isolation, morphological and ecological differentiation among cryptic species of Euchlanis dilatata, with the description of four new species. Hydrobiologia 844:221–242.

Koya, Y., R. Fujii, D. Tahara, H. Yambe, and T. Natsumeda. 2020. Effects of preventing a temperature decrease during winter on reproduction of male small-egged Kajika, Cottus pollux SE. Fish Physiology and Biochemistry 46:1243–1253.

Kupriyanova, E. K., and J. N. Havenhand. 2005. Effects of temperature on sperm swimming behaviour, respiration and fertilization success in the serpulid polychaete, Galeolaria caespitosa (Annelida: Serpulidae). Invertebrate Reproduction & Development 48:7–17.

Kwok, C. K., K. Y. Lam, S. M. Leung, A. P. Y. Chui, and P. O. Ang. 2016. Copper and thermal perturbations on the early life processes of the hard coral Platygyra acuta. Coral Reefs 35:827–838.

Laabir, M., S. A. Poulet, R. P. Harris, D. W. Pond, A. Cueff, R. N. Head, and A. Ianora. 1998. Comparative study of the reproduction of Calanus helgolandicus in well-mixed and seasonally stratified coastal waters of the western English Channel. Journal of Plankton Research 20:407–421.

Lacaze, E., A. Devaux, G. Jubeaux, R. Mons, M. Gardette, S. Bony, J. Garric, and O. Geffard. 2011. DNA damage in Gammarus fossarum sperm as a biomarker of genotoxic pressure: intrinsic variability and reference level. Science of The Total Environment 409:3230–3236.

Lahnsteiner, F., and S. Caberlotto. 2012. Motility of gilthead seabream Sparus aurata spermatozoa and its relation to temperature, energy metabolism and oxidative stress. Aquaculture 370–371:76–83.

Lahnsteiner, F., and M. Kletzl. 2012. The effect of water temperature on gamete maturation and gamete quality in the European grayling (Thymalus thymallus) based on experimental data and on data from wild populations. Fish Physiology and Biochemistry 38:455–467.

Lahnsteiner, F., and S. Leitner. 2013. Effect of Temperature on Gametogenesis and Gamete Quality in Brown Trout, Salmo trutta. Journal of Experimental Zoology Part A: Ecological Genetics and Physiology 319:138–148.

Lanna, E., B. Cajado, C. Santos-da-Silva, J. Da Hora, U. Porto, and V. Vasconcellos. 2018. Is the Orton's rule still valid? Tropical sponge fecundity, rather than periodicity, is modulated by temperature and other proximal cues. Hydrobiologia 815:187–205.

Lanna, E., and M. Klautau. 2018. Life history and reproductive dynamics of the cryptogenic calcareous sponge Sycettusa hastifera (Porifera, Calcarea) living in tropical rocky shores. Journal of the Marine Biological Association of the United Kingdom 98:505–514.

Le Clus, F. 1992. Seasonal trends in sea surface temperature, dry mass per oocyte and batch fecundity of pilchard Sardinops ocellatus in the northern Benguela system. South African Journal of Marine Science 12:123–134.

Le, M. H., H. K. Lim, B. H. Min, M. S. Park, M.-H. Son, J. U. Lee, and Y. J. Chang. 2011. Effects of varying dilutions, pH, temperature and cations on spermatozoa motility in fish Larimichthys polyactis. Journal of Environmental Biology 32:271.

Leach, T. S., B. BuyanUrt, and G. E. Hofmann. 2021. Exploring impacts of marine heatwaves: paternal heat exposure diminishes fertilization success in the purple sea urchin (Strongylocentrotus purpuratus). Marine Biology 168:103.

Ledet, J., M. Byrne, and A. G. B. Poore. 2018. Temperature effects on a marine herbivore depend strongly on diet across multiple generations. Oecologia 187:483–494.

Lee, H.-W. 2003. Effect of temperature on development, growth and reproduction in the marine copepod Pseudocalanus newmani at satiating food condition. Journal of Plankton Research 25:261–271.

Lee, M.-C., D.-S. Yoon, Y. Lee, H. Choi, K.-H. Shin, H. G. Park, and J.-S. Lee. 2020. Effects of low temperature on longevity and lipid metabolism in the marine rotifer Brachionus koreanus. Comparative Biochemistry and Physiology Part A: Molecular & Integrative Physiology 250:110803.

Lee, S.-H., M.-C. Lee, J. Puthumana, J. C. Park, S. Kang, J. Han, K.-H. Shin, H. G. Park, A.-S. Om, and J.-S. Lee. 2017. Effects of temperature on growth and fatty acid synthesis in the cyclopoid copepod Paracyclopina nana. Fisheries Science 83:725–734.

Limpanont, Y., H.-S. Yang, S.-H. Won, S.-J. Han, J.-B. Lee, B.-G. Lee, and K.-S. Choi. 2010. First report on the annual reproductive cycle of Burchardi's cockle, Acrosterigma (= Vasticardium) burchardi Dunker 1877 (Bivalvia: Cardiidae) on a subtidal sand flat off southern Jeju Island, Korea. Invertebrate Reproduction & Development 54:27–34.

Lin, Q., J. Lu, Y. Gao, L. Shen, J. Cai, and J. Luo. 2006. The effect of temperature on gonad, embryonic development and survival rate of juvenile seahorses, Hippocampus kuda Bleeker. Aquaculture 254:701–713.

Liu, S., S. Zhang, X. Ru, L. Sun, J. Li, Y. Zhou, and H. Yang. 2016. Effect of high temperature stress on the fertility of male and female gametes of the sea cucumber Apostichopus japonicus. Aquaculture Research 47:3127–3135.

Liu, X., D. Beyrend, G. Dur, and S. Ban. 2015. Combined effects of temperature and food concentration on growth and reproduction of Eodiaptomus japonicus (Copepoda: Calanoida) from Lake Biwa (Japan). Freshwater Biology 60:2003–2018.

Lopes, A. F., A. M. Faria, and S. Dupont.Elevated temperature, but not decreased pH, impairs reproduction in a temperate fish. Scientific Reports 10.

López-Galindo, L., C. Galindo-Sánchez, A. Olivares, O. H. Avila-Poveda, F. Díaz, O. E. Juárez, F. Lafarga, J. Pantoja-Pérez, C. Caamal-Monsreal, and C. Rosas. 2019. Reproductive performance of Octopus maya males conditioned by thermal stress. Ecological Indicators 96:437–447.

Lymbery, R. A., J. P. Evans, and W. J. Kennington. 2020. Post-ejaculation thermal stress causes changes to the RNA profile of sperm in an external fertilizer. Proceedings of the Royal Society B: Biological Sciences 287:20202147.

Ma, Q., Y.-L. Xi, J.-Y. Zhang, X.-L. Wen, and X.-L. Xiang. 2010. Differences in life table demography among eight geographic populations of Brachionus calyciflorus (Rotifera) from China. Limnologica 40:16–22.

Maier, G. 1993. The Life Histories of Two Temporarily Coexisting, Pond Dwelling Cladocerans. Internationale Revue der gesamten Hydrobiologie und Hydrographie 78:83–93.

Majhi, S. K., and A. R. Rasal. 2014. [No title found]. Turkish Journal of Fisheries and Aquatic Sciences 14.

Mak, K. K.-Y., and K. Y. K. Chan. 2018. Interactive effects of temperature and salinity on early life stages of the sea urchin Heliocidaris crassispina. Marine Biology 165:57.

Maneiro, V., A. Silva, A. J. Pazos, J. L. Sánchez, and M. L. Pérez-Parallé. 2017. Effects of temperature and photoperiod on the conditioning of the flat oyster (Ostrea edulis L.) in autumn. Aquaculture Research 48:4554–4562.

Maranhão, P., N. Bengala, M. Pardal, and J. C. Marques. 2001. The influence of environmental factors on the population dynamics, reproductive biology and productivity of Echinogammarus marinus Leach (Amphipoda, Gammaridae) in the Mondego estuary (Portugal). Acta Oecologica 22:139–152.

Martínez-Jerónimo, F. 2012. Description of the individual growth of Daphnia magna (Crustacea: Cladocera) through the von Bertalanffy growth equation. Effect of photoperiod and temperature. Limnology 13:65–71.

Martinez-Jeronimo, F., and C. Ventura-Lopez. 2011. Population dynamics of the tropical cladoceran Ceriodaphnia rigaudi Richard, 1894 (Crustacea: Anomopoda). Effect of food type and temperature. Journal of Environmental Biology 32:513.

Martínez, G., C. Aguilera, and L. Mettifogo. 2000. Interactive effects of diet and temperature on reproductive conditioning of Argopecten purpuratus broodstock. Aquaculture 183:149–159.

Martínez, G., and H. Pérez. 2003. Effect of different temperature regimes on reproductive conditioning in the scallop Argopecten purpuratus. Aquaculture 228:153–167.

Masclaux, Hé., A. Bec, M. J. Kainz, C. Desvilettes, L. Jouve, and G. Bourdier. 2009. Combined effects of food quality and temperature on somatic growth and reproduction of two freshwater cladocerans. Limnology and Oceanography 54:1323–1332.

Matias, D., S. Joaquim, A. M. Matias, and A. Leitão. 2016. Reproductive effort of the European clam Ruditapes decussatus (Linnaeus, 1758): influence of different diets and temperatures. Invertebrate Reproduction & Development 60:49–58.

Mazaris, A. D., A. S. Kallimanis, S. P. Sgardelis, and J. D. Pantis. 2008. Do long-term changes in sea surface temperature at the breeding areas affect the breeding dates and reproduction performance of Mediterranean loggerhead turtles? Implications for climate change. Journal of Experimental Marine Biology and Ecology 367:219–226.

McBride, S. C., W. D. Pinnix, J. M. Lawrence, A. L. Lawrence, and T. M. Mulligan. 1997. The Effect of Temperature on Production of Gonads by the Sea Urchin Strongylocentrotus franciscanus Fed Natural and Prepared Diets. Journal of the World Aquaculture Society 28:357–365.

McKenzie, V. J., W. E. Hall, and R. P. Guralnick. 2013. New Zealand mudsnails (Potamopyrgus antipodarum) in Boulder Creek, Colorado: environmental factors associated with fecundity of a parthenogenic invader. Canadian Journal of Zoology 91:30–36.

Medina, G. R., J. Goenaga, F. Hontoria, G. Cohen, and F. Amat. 2007. Effects of temperature and salinity on prereproductive life span and reproductive traits of two species of Artemia (Branchiopoda, Anostraca) from Argentina: Artemia franciscana and A. persimilis. Hydrobiologia 579:41–53.

Mehlis, M., and T. C. M. Bakker. 2014. The influence of ambient water temperature on sperm performance and fertilization success in three-spined sticklebacks (Gasterosteus aculeatus). Evolutionary Ecology 28:655–667.

Meza-Lopez, M. M., and E. Siemann. 2020. Warming alone increased exotic snail reproduction and together with eutrophication influenced snail growth in native wetlands but did not impact plants. Science of The Total Environment 704:135271.

Mita, M., A. Hino, and I. Yasumasu. 1984. EFFECT OF TEMPERATURE ON INTERACTION BETWEEN EGGS AND SPERMATOZOA OF SEA URCHIN. The Biological Bulletin 166:68–77.

Morgan, A. D. 2009. Spawning of the Temperate Sea Cucumber, Australostichopus mollis (Levin). Journal of the World Aquaculture Society 40:363–373.

Morgan, M., R. Rideout, and E. Colbourne. 2010. Impact of environmental temperature on Atlantic cod Gadus morhua energy allocation to growth, condition and reproduction. Marine Ecology Progress Series 404:185–195.

Moss, G. A. 1998. Effect of temperature on the breeding cycle and spawning success of the New Zealand abalone, Haliotis australis. New Zealand Journal of Marine and Freshwater Research 32:139–146.

Múgica, M., I. M. Sokolova, U. Izagirre, and I. Marigómez. 2015. Season-dependent effects of elevated temperature on stress biomarkers, energy metabolism and gamete development in mussels. Marine Environmental Research 103:1–10.

Mull, C. G., C. G. Lowe, and K. A. Young. 2008. Photoperiod and water temperature regulation of seasonal reproduction in male round stingrays (Urobatis halleri). Comparative Biochemistry and Physiology Part A: Molecular & Integrative Physiology 151:717–725.

Nagy, S. T., B. Kakasi, L. Pál, M. Havasi, M. Bercsényi, and F. Husvéth. 2016. Effects of high ambient temperature on fish sperm plasma membrane integrity and mitochondrial activity — A flow cytometric study. Acta Biologica Hungarica 67:125–132.

Nash, S., and M. S. Rahman. 2019. Short-term heat stress impairs testicular functions in the American oyster, Crassostrea virginica : Molecular mechanisms and induction of oxidative stress and apoptosis in spermatogenic cells. Molecular Reproduction and Development 86:1444–1458.

Neila, A.-T., D.-Y. Mohamed Néjib, B. Genuario, A. Lotfi, and A. Habib. 2012. Impacts of very warm temperature on egg production rates of three Acartiidae (Crustacea, Copepoda) in a Northern African lagoon. Journal of Thermal Biology 37:445–453.

Newman, D. M., P. L. Jones, and B. A. Ingram. 2010. Advanced ovarian development of Murray cod Maccullochella peelii peelii via phase-shifted photoperiod and two temperature regimes. Aquaculture 310:206–212.

Nguyen, T. T., M. Le, N. X. Doan, S. T. Nguyen, T. S. H. Truong, M. T. T. Vu, and K. V. Dinh. 2020. Salinity and temperature effects on productivity of a tropical calanoid copepod Pseudodiaptomus incisus. Aquaculture Research 51:3768–3779.

Nissling, A., U. Johansson, and M. Jacobsson. 2006. Effects of salinity and temperature conditions on the reproductive success of turbot (Scophthalmus maximus) in the Baltic Sea. Fisheries Research 80:230–238.

Ogello, E., H.-J. Kim, K. Suga, and A. Hagiwara. 2016. Lifetable demography and population growth of the rotifer Brachionus angularis in Kenya: influence of temperature and food density. African Journal of Aquatic Science 41:329–336.

Oliveira, G. F., H. Siregar, H. Queiroga, and L. G. Peteiro. 2021. Main Drivers of Fecundity Variability of Mussels along a Latitudinal Gradient: Lessons to Apply for Future Climate Change Scenarios. Journal of Marine Science and Engineering 9:759.

Oyarzún, P. A., J. E. Toro, J. Garcés-Vargas, C. Alvarado, R. Guiñez, R. Jaramillo, C. Briones, and B. Campos. 2018. Reproductive patterns of mussel Perumytilus purpuratus (Bivalvia: Mytilidae), along the Chilean coast: effects caused by climate change? Journal of the Marine Biological Association of the United Kingdom 98:375–385.

Pan, L., Y.-L. Xi, H.-Y. Cao, B. Peng, and J.-X. Wang. 2014. Combined effects of temperature and prey (Brachionus angularis) density on life-table demography and population growth of Asplanchna brightwelli (Rotifera). Annales de Limnologie - International Journal of Limnology 50:261–268.

Pankhurst, N. W., H. R. King, K. Anderson, A. Elizur, P. M. Pankhurst, and N. Ruff. 2011. Thermal impairment of reproduction is differentially expressed in maiden and repeat spawning Atlantic salmon. Aquaculture 316:77–87.

Pankhurst, N. W., G. J. Purser, G. Van Der Kraak, P. M. Thomas, and G. N. R. Forteath. 1996. Effect of holding temperature on ovulation, egg fertility, plasma levels of reproductive hormones and in vitro ovarian steroidogenesis in the rainbow trout Oncorhynchus mykiss. Aquaculture 146:277–290.

Papandroulakis, N., M. Suquet, M. T. Spedicato, A. Machias, C. Fauvel, and P. Divanach. 2004. Feeding Rates, Growth Performance and Gametogenesis of Wreckfish (Polyprion americanus) kept in Captivity. Aquaculture International 12:395–407.

Park, C.-B., Y. J. Kim, and K. Soyano. 2017. Effects of increasing temperature due to aquatic climate change on the self-fertility and the sexual development of the hermaphrodite fish, Kryptolebias marmoratus. Environmental Science and Pollution Research 24:1484–1494.

Parker, L. M., P. M. Ross, and W. A. O’Connor. 2010. Comparing the effect of elevated pCO2 and temperature on the fertilization and early development of two species of oysters. Marine Biology 157:2435–2452.

Pascual, C., E. Valera, C. Re-Regis, G. Gaxiola, A. Sanchez, L. Ramos, L. A. Soto, and C. Rosas. 1998. Effect of Water Temperature on Reproductive Tract Condition of Penaeus setiferus Adult Males. Journal of the World Aquaculture Society 29:477–484.

Pavón-Meza, E. L., S. S. S. Sarma, and S. Nandini. 2005. Combined Effects of Algal (Chlorella vulgaris) Food Level and Temperature on the Demography of Brachionus havanaensis (Rotifera): a Life Table Study. Hydrobiologia 546:353–360.

Paxton, C. W., M. V. B. Baria, V. M. Weis, and S. Harii. 2016. Effect of elevated temperature on fecundity and reproductive timing in the coral Acropora digitifera. Zygote 24:511–516.

Perez-Miguel, M., P. Drake, and J. A. Cuesta. 2020. Temperature effect on the African pea crab Afropinnotheres monodi: Embryonic and larval developments, fecundity and adult survival. Journal of Experimental Marine Biology and Ecology 527:151380.

Pham, H. T., K. V. Dinh, C. C. Nguyen, and L. B. Quoc. 2020. Changes in the Magnitude of the Individual and Combined Effects of Contaminants, Warming, and Predators on Tropical Cladocerans across 11 Generations. Environmental Science & Technology 54:15287–15295.

Philippe, C., P. Hautekiet, A. F. Grégoir, E. S. J. Thoré, L. Brendonck, G. De Boeck, and T. Pinceel. 2019. Interactive effects of 3,4-DCA and temperature on the annual killifish Nothobranchius furzeri. Aquatic Toxicology 212:146–153.

Pía-Fernández, J., F. Belén-Chaar, L. Epherra, J.-M. González-Aravena, and T. Rubilar. 2021. Embryonic and larval development is conditioned by water temperature and maternal origin of eggs in the sea urchin Arbacia dufresnii (Echinodermata: Echinoidea). Revista de Biología Tropical 69:S452–S463.

Pitts, K. A., J. E. Campbell, J. Figueiredo, and N. D. month = apr Fogarty. 2020. Ocean acidification partially mitigates the negative effects of warming on the recruitment of the coral, {Orbicella} faveolata. CORAL REEFS 39:281–292.

Planas, M., P. Quintas, and A. Chamorro. 2013. Maturation of Hippocampus guttulatus and Hippocampus hippocampus females by manipulation of temperature and photoperiod regimes. Aquaculture 388–391:147–152.

Postingel Quirino, P., M. Da Silva Rodrigues, E. M. Da Silva Cabral, D. H. De Siqueira-Silva, R. H. Mori, A. J. Butzge, R. H. Nóbrega, A. Ninhaus-Silveira, and R. Veríssimo-Silveira. 2021. The influence of increased water temperature on the duration of spermatogenesis in a neotropical fish, Astyanax altiparanae (Characiformes, Characidae). Fish Physiology and Biochemistry 47:747–755.

Pountney, S. M., I. Lein, H. Migaud, and A. Davie. 2020. High temperature is detrimental to captive lumpfish (Cyclopterus lumpus, L) reproductive performance. Aquaculture 522:735121.

Prato, E., F. Biandolino, and C. Scardicchio. 2008. IMPLICATIONS FOR TOXICITY TESTS WITH AMPHIPOD GAMMARUS AEQUICAUDA: EFFECTS OF

TEMPERATURE AND SALINITY ON LIFE CYCLE. Environmental Technology 29:1349–1356.

Pretterebner, K., and L. M. Pardo. 2020. All or nothing: Switch to high current reproductive investment under risk of starvation in male kelp crab. Ecology and Evolution 10:3383–3391.

Pretterebner, K., L. M. Pardo, and K. Paschke. 2019. Temperature-dependent seminal recovery in the southern king crab Lithodes santolla. Royal Society Open Science 6:181700.

Purchase, C. F., I. A. E. Butts, A. Alonso-Fernández, and E. A. Trippel. 2010. Thermal reaction norms in sperm performance of Atlantic cod (Gadus morhua). Canadian Journal of Fisheries and Aquatic Sciences 67:498–510.

Rahman, M. S., M. Tsuchiya, and T. Uehara. 2009. Effects of Temperature on Gamete Longevity and Fertilization Success in Two Sea Urchin Species, Echinometra mathaei and Tripneustes gratilla. Zoological Science 26:1–8.

Rao, M. V. H., S. Ghosh, K. Sreeramulu, V. U. Mahesh, M. S. Kumar, and M. Muktha. 2017. Reproductive biology of Nemipterus japonicus (Bloch, 1791) in the trawl grounds along the north-east coast of India. Indian Journal of Fisheries 64.

Rhyne, A. L., C. L. Ohs, and E. Stenn. 2009. Effects of temperature on reproduction and survival of the calanoid copepod Pseudodiaptomus pelagicus. Aquaculture 292:53–59.

Rijnsdorp, A. D., R. E. Grift, and S. B. Kraak. 2005. Fisheries-induced adaptive change in reproductive investment in North Sea plaice (Pleuronectes platessa)? Canadian Journal of Fisheries and Aquatic Sciences 62:833–843.

Romagosa, E., B. E. Souza, E. A. Sanches, D. M. Baggio, and R. A. Bombardelli. 2010. Sperm motility of Prochilodus lineatus in relation to dilution rate and temperature of the activating medium: Sperm motility of Prochilodus. Journal of Applied Ichthyology 26:678–681.

Rossi, V., A. Martorella, D. Scudieri, and P. Menozzi. 2017. Seasonal niche partitioning and coexistence of amphimictic and parthenogenetic lineages of Heterocypris barbara (Crustacea: Ostracoda). Canadian Journal of Zoology 95:7–14.

Ryan, W., and T. Miller. 2019. Reproductive strategy changes across latitude in a clonal sea anemone. Marine Ecology Progress Series 611:129–141.

Sachlikidis, N. G., C. M. Jones, and J. E. Seymour. 2005. Reproductive cues in Panulirus ornatus. New Zealand Journal of Marine and Freshwater Research 39:305–310.

Santidrián Tomillo, P., D. Oro, F. V. Paladino, R. Piedra, A. E. Sieg, and J. R. Spotila. 2014. High beach temperatures increased female-biased primary sex ratios but reduced output of female hatchlings in the leatherback turtle. Biological Conservation 176:71–79.

Santos, M. R., N. Vieira, and N. M. Monteiro. 2018. High temperatures disrupt Artemia franciscana mating patterns and impact sexual selection intensity. Estuarine, Coastal and Shelf Science 207:209–214.

Santos, P. M., P. Albano, A. Raposo, S. M. F. Ferreira, J. L. Costa, and A. Pombo. 2020. The effect of temperature on somatic and gonadal development of the sea urchin Paracentrotus lividus (Lamarck, 1816). Aquaculture 528:735487.

Sharma, S. S., A. C. Alfaro, N. L. Ragg, and L. N. Zamora. 2020. Effects of temperature on early development of the New Zealand geoduck Panopea zelandica (Quoy & Gaimard, 1835). Aquaculture Research 51:751–760.

Simonini, R., and D. Prevedelli. 2003. Effects of temperature on two Mediterranean populations of Dinophilus gyrociliatus (Polychaeta: Dinophilidae). Journal of Experimental Marine Biology and Ecology 291:79–93.

Sogabe, A., and Y. Yanagisawa. 2007. Sex-role reversal of a monogamous pipefish without higher potential reproductive rate in females. Proceedings of the Royal Society B: Biological Sciences 274:2959–2963.

Spencer, L. H., Y. R. Venkataraman, R. Crim, S. Ryan, M. J. Horwith, and S. B. Roberts. 2020. Carryover effects of temperature and pCO 2 across multiple Olympia oyster populations. Ecological Applications 30:e02060.

Staines, M. N., D. T. Booth, and C. J. Limpus. 2019. Microclimatic effects on the incubation success, hatchling morphology and locomotor performance of marine turtles. Acta Oecologica 97:49–56.

Stanwell-Smith, D., and L. S. Peck. 1998. Temperature and Embryonic Development in Relation to Spawning and Field Occurrence of Larvae of Three Antarctic Echinoderms. The Biological Bulletin 194:44–52.

Sunitha, M. S., and V. Jayaprakas. 1997. Influence of pH, temperature, salinity and media on activation of motility and short term preservation of spermatozoa of an estuarine fish, Mystus gulio (Hamilton)(Siluridae-Pisces).

Takasuka, A., Y. Oozeki, H. Kubota, Y. Tsuruta, and T. Funamoto. 2005. Temperature impacts on reproductive parameters for Japanese anchovy: Comparison between inshore and offshore waters. Fisheries Research 76:475–482.

Tanasichuk, R. W., and D. M. Ware. 1987. Influence of Interannual Variations in Winter Sea Temperature on Fecundity and Egg Size in Pacific Herring (Clupea harengus pallasi). Canadian Journal of Fisheries and Aquatic Sciences 44:1485–1495.

Targońska, K., K. Kupren, D. Żarski, R. Król, and D. Kucharczyk. 2011. Influence of thermal conditions on successful ide (Leuciscus idus L.) artificial reproduction during spawning season. Italian Journal of Animal Science 10:e50.

Tarkan, A. N., Ö. Gaygusuz, A. S. Tarkan, Ç. Gürsoy, and H. Acipinar. 2007. Interannual Variability of Fecundity and Egg Size of an Invasive Cyprinid, Carassius gibelio : Effects of Density-Dependent and Density-Independent Factors. Journal of Freshwater Ecology 22:11–17.

Temperature differentially affects male and female reproductive rates in the sand goby: consequences for operational sex ratio. 1994. . Proceedings of the Royal Society of London. Series B: Biological Sciences 256:151–156.

Tropea, C., Y. Piazza, and L. S. L. Greco. 2010. Effect of long-term exposure to high temperature on survival, growth and reproductive parameters of the “redclaw” crayfish Cherax quadricarinatus. Aquaculture 302:49–56.

Tunçelli, G., and D. Memiş. 2021. Effects of constant and changing water temperature on sperm quality of the endangered Çoruh trout, Salmo coruhensis. Journal of Applied Aquaculture 33:209–220.

Tveiten, H., and H. K. Johnsen. 1999. Temperature experienced during vitellogenesis influences ovarian maturation and the timing of ovulation in common wolffish. Journal of Fish Biology 55:809–819.

Tveiten, H., S. E. Solevåg, and H. K. Johnsen. 2001. Holding temperature during the breeding season influences final maturation and egg quality in common wolffish. Journal of Fish Biology 58:374–385.

Uye, S., and K. Sano. 1995. Seasonal reproductive biology of the small cyclopoid copepod Oithona davisae in a temperate eutrophic inlet. Marine Ecology Progress Series 118:121–128.

Uye, S., and N. Shibuno. 1992. Reproductive biology of the planktonic copepod Paracalanus sp. in the Inland Sea of Japan. Journal of Plankton Research 14:343–358.

Valdebenito, I., J. Cosson, P. Contreras, J. C. Sánchez, R. P. S. Oliveira, J. Risopatrón, J. G. Farías, and E. Figueroa. 2017. Spermatological research of experimentally farmed Patagonian blenny (Eleginops maclovinus) (Perciformes: Eleginopsidae) in Chile. Aquaculture Research 48:4197–4204.

Valdizan, A., P. Beninger, P. Decottignies, M. Chantrel, and B. Cognie. 2011. Evidence that rising coastal seawater temperatures increase reproductive output of the invasive gastropod Crepidula fornicata. Marine Ecology Progress Series 438:153–165.

Vilchis, L. I., M. J. Tegner, J. D. Moore, C. S. Friedman, K. L. Riser, T. T. Robbins, and P. K. Dayton. 2005. OCEAN WARMING EFFECTS ON GROWTH, REPRODUCTION, AND SURVIVORSHIP OF SOUTHERN CALIFORNIA ABALONE. Ecological Applications 15:469–480.

Villegas-Hernández, H., J. Lloret, and M. Muñoz. 2015. Reproduction, condition and abundance of the Mediterranean bluefish (Pomatomus saltatrix) in the context of sea warming. Fisheries Oceanography 24:42–56.

Vladic, T. 1997. Sperm motility and fertilization time span in Atlantic salmon and brown trout—the effect of water temperature. Journal of Fish Biology 50:1088–1093.

Vladić, T. 2000. The effect of water temperature on sperm motility of adult male and precocious male parr of Atlantic salmon and brown trout. SIL Proceedings, 1922-2010 27:1070–1074.

Volcan, M. V., L. A. Sampaio, D. C. Bongalhardo, and R. B. Robaldo. 2013. Reproduction of the annual fish Austrolebias nigrofasciatus (Rivulidae) maintained at different temperatures. Journal of Applied Ichthyology 29:648–652.

Wang, J., X. Jiang, and X. Feng. 2011. Impact of external factors on sperm motility of Sepiella maindroni. Chinese Journal of Oceanology and Limnology 29:184–191.

Wang, S., G. Zhang, K. Zhou, and S. Sun. 2020. Long-term population variability and reproductive strategy of a northward expanded ctenophore Pleurobrachia globosa Moser, 1903 in a temperate bay, China. Journal of Experimental Marine Biology and Ecology 533:151457.

Weddle, G. K., and B. M. Burr. 1991. Fecundity and the Dynamics of Multiple Spawning in Darters: An In-Stream Study of Etheostoma rafinesquei. Copeia 1991:419.

Weisse, T., N. Laufenstein, and G. Weithoff. 2013. Multiple environmental stressors confine the ecological niche of the rotifer Cephalodella acidophila. Freshwater Biology 58:1008–1015.

Williamson, C. E., and N. M. Butler. 1987. Temperature, food and mate limitation of copepod reproductive rates: separating the effects of multiple hypotheses. Journal of Plankton Research 9:821–836.

Williot, P., E. Kopeika, and B. month = sep Goncharov. 2000. Influence of testis state, temperature and delay in semen collection on spermatozoa motility in the cultured {Siberian} sturgeon ({Acipenser} baeri {Brandt. AQUACULTURE 189:53–61.

Wright, P. J., J. E. Orpwood, and P. Boulcott. 2017. Warming delays ovarian development in a capital breeder. Marine Biology 164:80.

Xue, S., J. Fang, J. Zhang, Z. Jiang, Y. Mao, and F. Zhao. 2013. Effects of temperature and salinity on the development of the amphipod crustacean Eogammarus sinensis. Chinese Journal of Oceanology and Limnology 31:1010–1017.

Yoneda, M., H. Kitano, H. Tanaka, K. Kawamura, S. Selvaraj, S. Ohshimo, M. Matsuyama, and A. Shimizu. 2014. Temperature- and income resource availability-mediated variation in reproductive investment in a multiple-batch-spawning Japanese anchovy. Marine Ecology Progress Series 516:251–262.

Yoneda, M., and P. J. Wright. 2005a. Effect of temperature and food availability on reproductive investment of first-time spawning male Atlantic cod, Gadus morhua. ICES Journal of Marine Science 62:1387–1393.

Yoneda, M., and P. J. Wright. 2005b. Effects of varying temperature and food availability on growth and reproduction in first-time spawning female Atlantic cod. Journal of Fish Biology 67:1225–1241.

Zhang, C., M. Jansen, L. De Meester, and R. Stoks. 2019. Rapid evolution in response to warming does not affect the toxicity of a pollutant: Insights from experimental evolution in heated mesocosms. Evolutionary Applications 12:977–988.

Zhao, C., W. Feng, J. Wei, L. Zhang, P. Sun, and Y. Chang. 2016. Effects of temperature and feeding regime on food consumption, growth, gonad production and quality of the sea urchin Strongylocentrotus intermedius. Journal of the Marine Biological Association of the United Kingdom 96:185–195.

Zhao, C., L. Zhang, S. Qi, D. Shi, D. Yin, and Y. Chang. 2018. Multilevel effects of long-term elevated temperature on fitness related traits of the sea urchin Strongylocentrotus intermedius. Bulletin of Marine Science 94:1483–1497

Figure 1 Silhouette images - Alexis Simon (*Mytilus galloprovincialis*), Birgit Lang (*Pleuronectes platessa*), Cagri Cevrim (*Loligo vulgaris*), Carlos Cano-Barbacil (*Salmo trutta*), Frank Förster (based on a picture by Jerry Kirkhart; modified by T. Michael Keesey) (*Strongylocentrotus purpuratus*), Ingo Braasch (*Anguilla anguilla*), James R. Spotila and Ray Chatterji (*Caretta caretta*), Jebulon (vectorized by T. Michael Keesey) (*Cancer bellianus*), Julie Blommaert based on photo by Sofdrakou (*Brachionus plicatilis*), Mathilde Cordellier (*Daphnia magna*), Nathan Hermann (*Clupea harengus*), Niall McGinty (*Centropages typicus*, *Temora*), Prespa Research Group (*Gammarus roeselii*), Richard J. Harris (*Uranoscopus*), S. Ginot (*Physella acuta*), Thomas Hegna (*Artemia salina*), and others (*Acropora florida*, *Chaetostoma microps*, *Hippocampus*, *Pomatoschistus microps*).

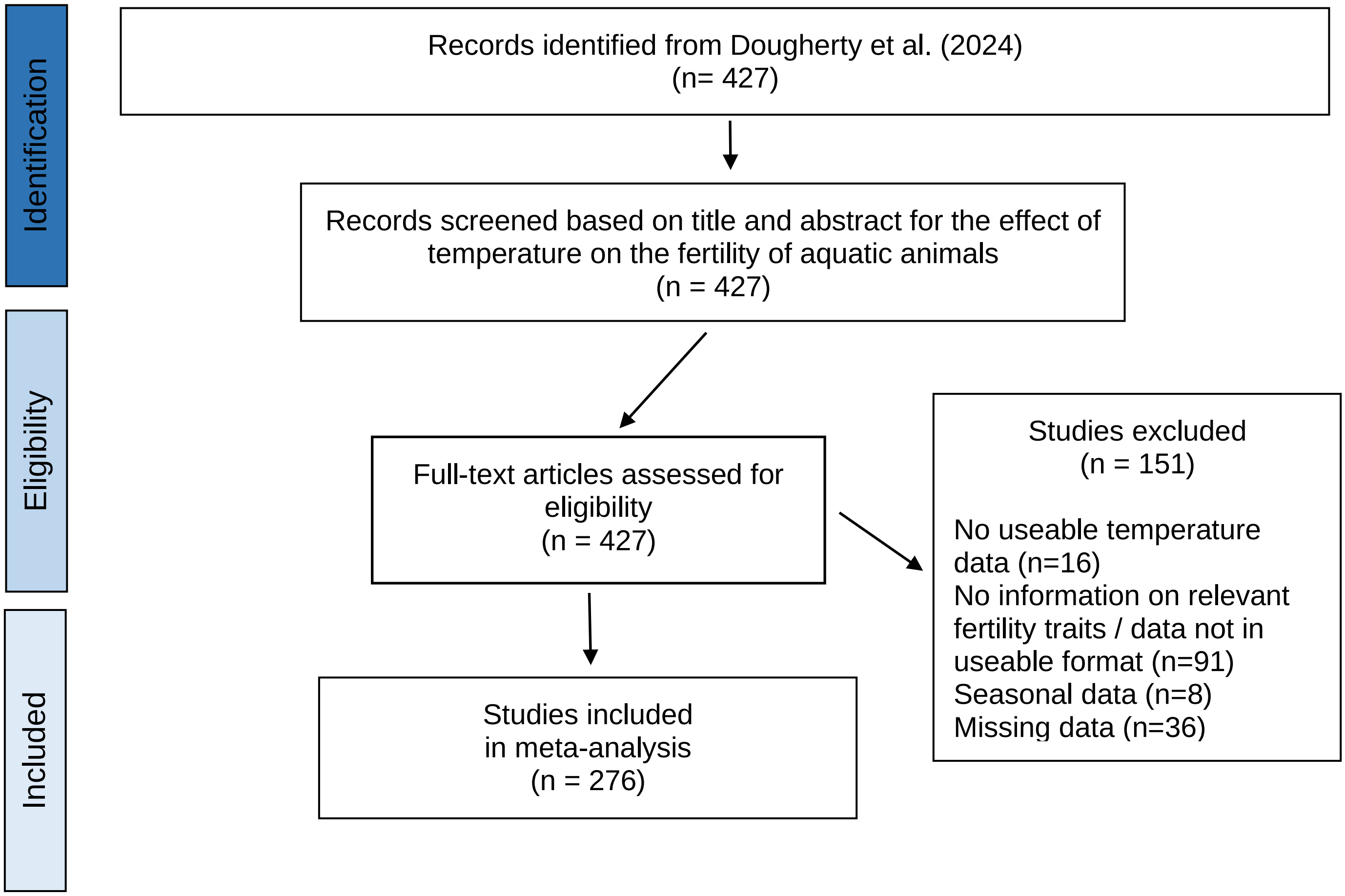
Identification
Eligibility
Included
Records identified from Dougherty et al. (2024)
(n= 427)
Records screened based on title and abstract for the effect of temperature on the fertility of aquatic animals
(n = 427)
Full-text articles assessed for eligibility
(n = 427)
Studies excluded
(n = 151)
No useable temperature data (n=16)
No information on relevant fertility traits / data not in useable format (n=91)
Seasonal data (n=8)
Missing data (n=36)
Studies included in meta-analysis
(n = 276)

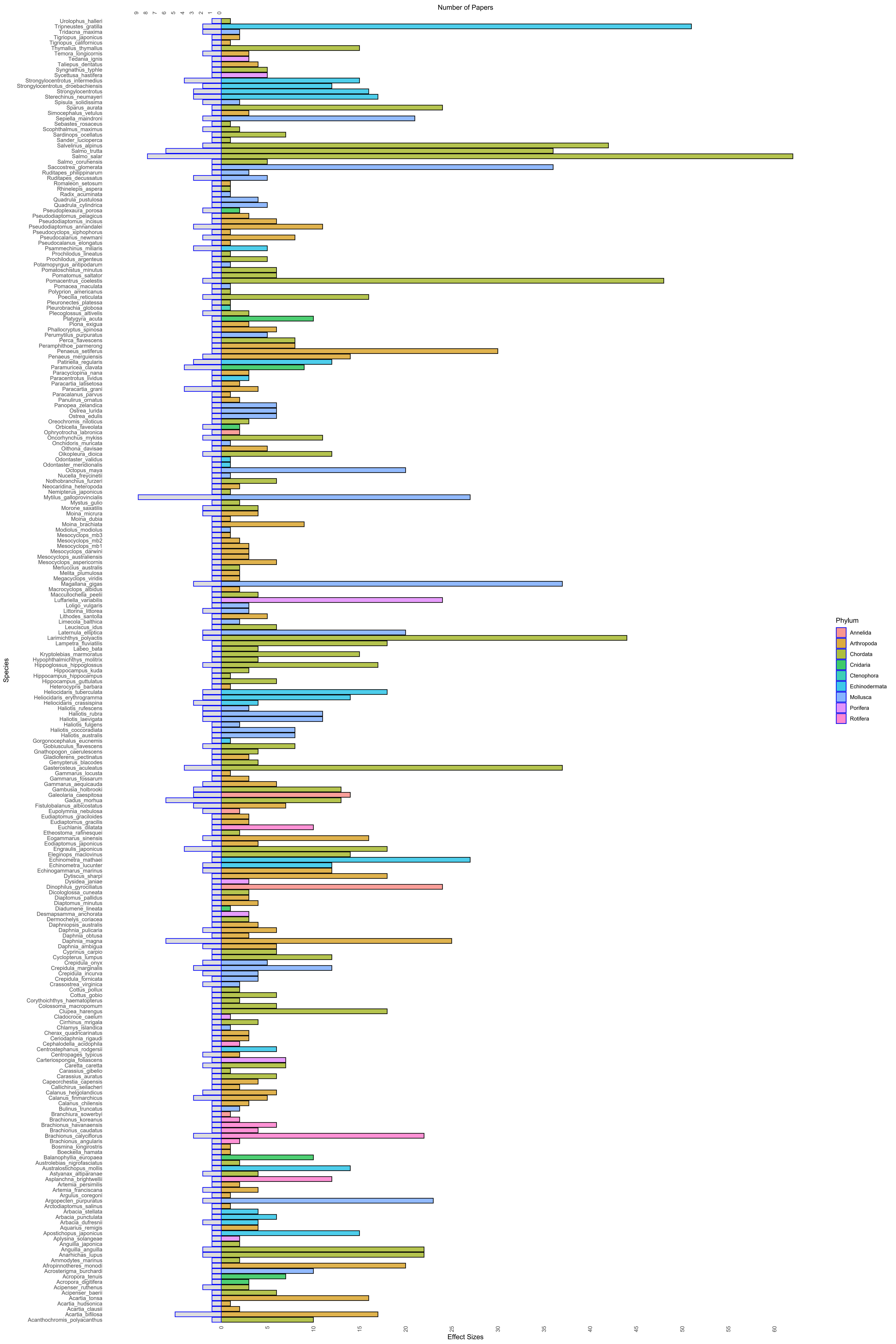
Number of Papers
9 8 7 6 5 4 3 2 1 0
Species
Urolophus_halleri
Tripneustes_gratilla
Tridacna_maxima
Tigriopus_japonicus
Tigriopus_californicus
Thymallus_thymallus
Temora_longicornis
Tedania_ignis
Taliepus_dentatus
Syngnathus_typhle
Sycettusa_hastifera
Strongylocentrotus_intermedius
Strongylocentrotus_droebachiensis
Strongylocentrotus
Sterechinus_neumayeri
Spisula_solidissima
Sparus_aurata
Simocephalus_vetulus
Sepiella_maindroni
Sebastes_rosaceus
Scophthalmus_maximus
Sardinops_ocellatus
Sander_lucioperca
Salvelinus_alpinus
Salmo_trutta
Salmo_salar
Salmo_coruhensis
Saccostrea_glomerata
Ruditapes_philippinarum
Ruditapes_decussatus
Romaleon_setosum
Rhinelepis_aspera
Radix_acuminata
Quadrula_pustulosa
Quadrula_cylindrica
Pseudoplexaura_porosa
Pseudodiaptomus_pelagicus
Pseudodiaptomus_incisus
Pseudodiaptomus_annandalei
Pseudocyclops_xiphophorus
Pseudocalanus_newmani
Pseudocalanus_elongatus
Psammechinus_miliaris
Prochilodus_lineatus
Prochilodus_argenteus
Potamopyrgus_antipodarum
Pomatoschistus_minutus
Pomatomus_saltator
Pomacentrus_coelestis
Pomacea_maculata
Polyprion_americanus
Poecilia_reticulata
Pleuronectes_platessa
Pleurobrachia_globosa
Plecoglossus_altivelis
Platygyra_acuta
Piona_exigua
Phallocryptus_spinosa
Perumytilus_purpuratus
Perca_flavescens
Peramphithoe_parmerong
Penaeus_setiferus
Penaeus_merguiensis
Patiriella_regularis
Paramuricea_clavata
Paracyclopina_nana
Paracentrotus_lividus
Paracartia_latisetosa
Paracartia_grani
Paracalanus_parvus
Panulirus_ornatus
Panopea_zelandica
Ostrea_lurida
Ostrea_edulis
Oreochromis_niloticus
Orbicella_faveolata
Ophryotrocha_labronica
Oncorhynchus_mykiss
Onchidoris_muricata
Oithona_davisae
Oikopleura_dioica
Odontaster_validus
Odontaster_meridionalis
Octopus_maya
Nucella_freycinetii
Nothobranchius_furzeri
Neocaridina_heteropoda
Nemipterus_japonicus
Mytilus_galloprovincialis
Mystus_gulio
Morone_saxatilis
Moina_micrura
Moina_dubia
Moina_brachiata
Modiolus_modiolus
Mesocyclops_mb3
Mesocyclops_mb2
Mesocyclops_mb1
Mesocyclops_darwini
Mesocyclops_australiensis
Mesocyclops_aspericornis
Merluccius_australis
Melita_plumulosa
Megacyclops_viridis
Magallana_gigas
Macrocyclops_albidus
Maccullochella_peelii
Luffariella_variabilis
Loligo_vulgaris
Littorina_littorea
Lithodes_santolla
Limecola_balthica
Leuciscus_idus
Laternula_elliptica
Larimichthys_polyactis
Lampetra_fluviatilis
Labeo_bata
Kryptolebias_marmoratus
Hypophthalmichthys_molitrix
Hippoglossus_hippoglossus
Hippocampus_kuda
Hippocampus_hippocampus
Hippocampus_guttulatus
Heterocypris_barbara
Heliocidaris_tuberculata
Heliocidaris_erythrogramma
Heliocidaris_crassispina
Haliotis_rufescens
Haliotis_rubra
Haliotis_laevigata
Haliotis_fulgens
Haliotis_coccoradiata
Haliotis_australis
Gorgonocephalus_eucnemis
Gobiusculus_flavescens
Gnathopogon_caerulescens
Gladioferens_pectinatus
Genypterus_blacodes
Gasterosteus_aculeatus
Gammarus_locusta
Gammarus_fossarum
Gammarus_aequicauda
Gambusia_holbrooki
Galeolaria_caespitosa
Gadus_morhua
Fistulobalanus_albicostatus
Eupolymnia_nebulosa
Eudiaptomus_graciloides
Eudiaptomus_gracilis
Euchlanis_dilatata
Etheostoma_rafinesquei
Eogammarus_sinensis
Eodiaptomus_japonicus
Engraulis_japonicus
Eleginops_maclovinus
Echinometra_mathaei
Echinometra_lucunter
Echinogammarus_marinus
Dytiscus_sharpi
Dysidea_janiae
Dinophilus_gyrociliatus
Dicologlossa_cuneata
Diaptomus_pallidus
Diaptomus_minutus
Diadumene_lineata
Desmapsamma_anchorata
Dermochelys_coriacea
Daphniopsis_australis
Daphnia_pulicaria
Daphnia_obtusa
Daphnia_magna
Daphnia_ambigua
Cyprinus_carpio
Cyclopterus_lumpus
Crepidula_onyx
Crepidula_marginalis
Crepidula_incurva
Crepidula_fornicata
Crassostrea_virginica
Cottus_pollux
Cottus_gobio
Corythoichthys_haematopterus
Colossoma_macropomum
Clupea_harengus
Cladocroce_caelum
Cirrhinus_mrigala
Chlamys_islandica
Cherax_quadricarinatus
Ceriodaphnia_rigaudi
Cephalodella_acidophila
Centrostephanus_rodgersii
Centropages_typicus
Carteriospongia_foliascens
Caretta_caretta
Carassius_gibelio
Carassius_auratus
Capoerchestia_capensis
Callichirus_seilacheri
Calanus_helgolandicus
Calanus_finmarchicus
Calanus_chilensis
Bulinus_truncatus
Branchiura_sowerbyi
Brachionus_koreanus
Brachionus_havanaensis
Brachionus_caudatus
Brachionus_calyciflorus
Brachionus_angularis
Bosmina_longirostris
Boeckella_hamata
Balanophyllia_europaea
Austrolebias_nigrofasciatus
Australostichopus_mollis
Astyanax_altiparanae
Asplanchna_brightwellii
Artemia_persimilis
Artemia_franciscana
Argulus_coregoni
Argopecten_purpuratus
Arctodiaptomus_salinus
Arbacia_stellata
Arbacia_punctulata
Arbacia_dufresnii
Aquarius_remigis
Apostichopus_japonicus
Aplysina_solangeae
Anguilla_japonica
Anguilla_anguilla
Anarhichas_lupus
Ammodytes_marinus
Afropinnotheres_monodi
Acrostrigma_burchardi
Acropora_tenuis
Acropora_digitifera
Acipenser_ruthenus
Acipenser_baerii
Acartia_tonsa
Acartia_hudsonica
Acartia_clausii
Acartia_bifilosa
Acanthochromis_polyacanthus
0 5 10 15 20 25 30 35 40 45 50 55 60
Effect Sizes
Phylum
Annelida
Arthropoda
Chordata
Cnidaria
Ctenophora
Echinodermata
Mollusca
Porifera
Rotifera

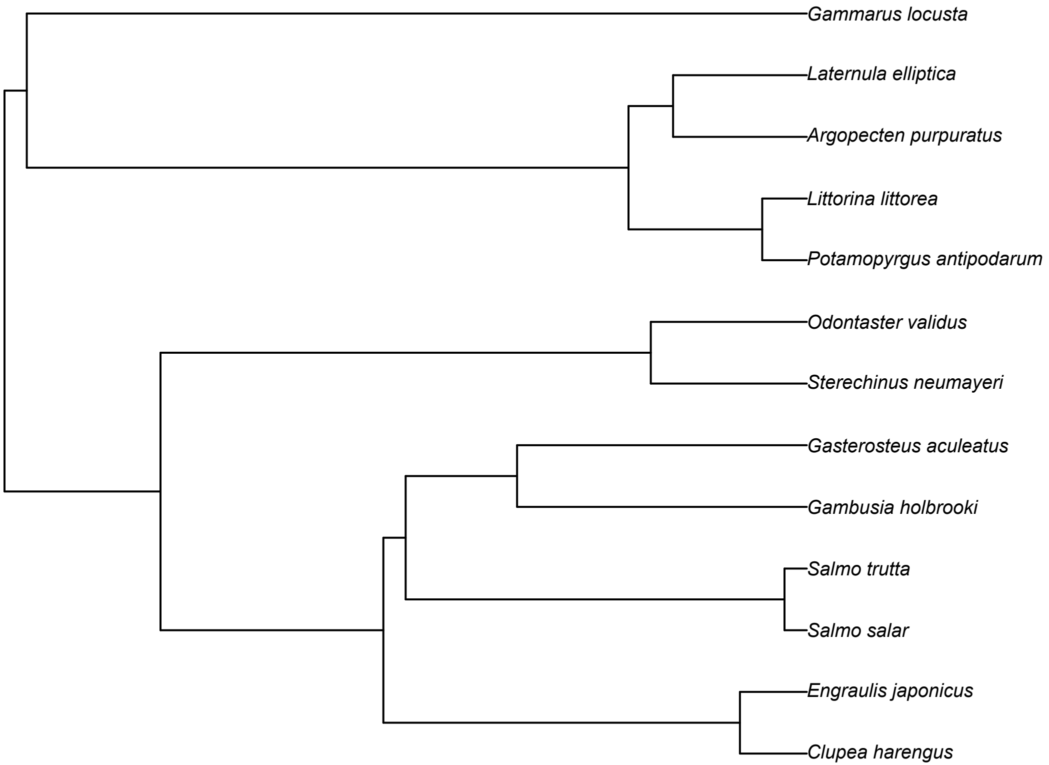
Gammarus locusta
Laternula elliptica
Argopecten purpuratus
Littorina littorea
Potamopyrgus antipodarum
Odontaster validus
Sterechinus neumayeri
Gasterosteus aculeatus
Gambusia holbrooki
Salmo trutta
Salmo salar
Engraulis japonicus
Clupea harengus

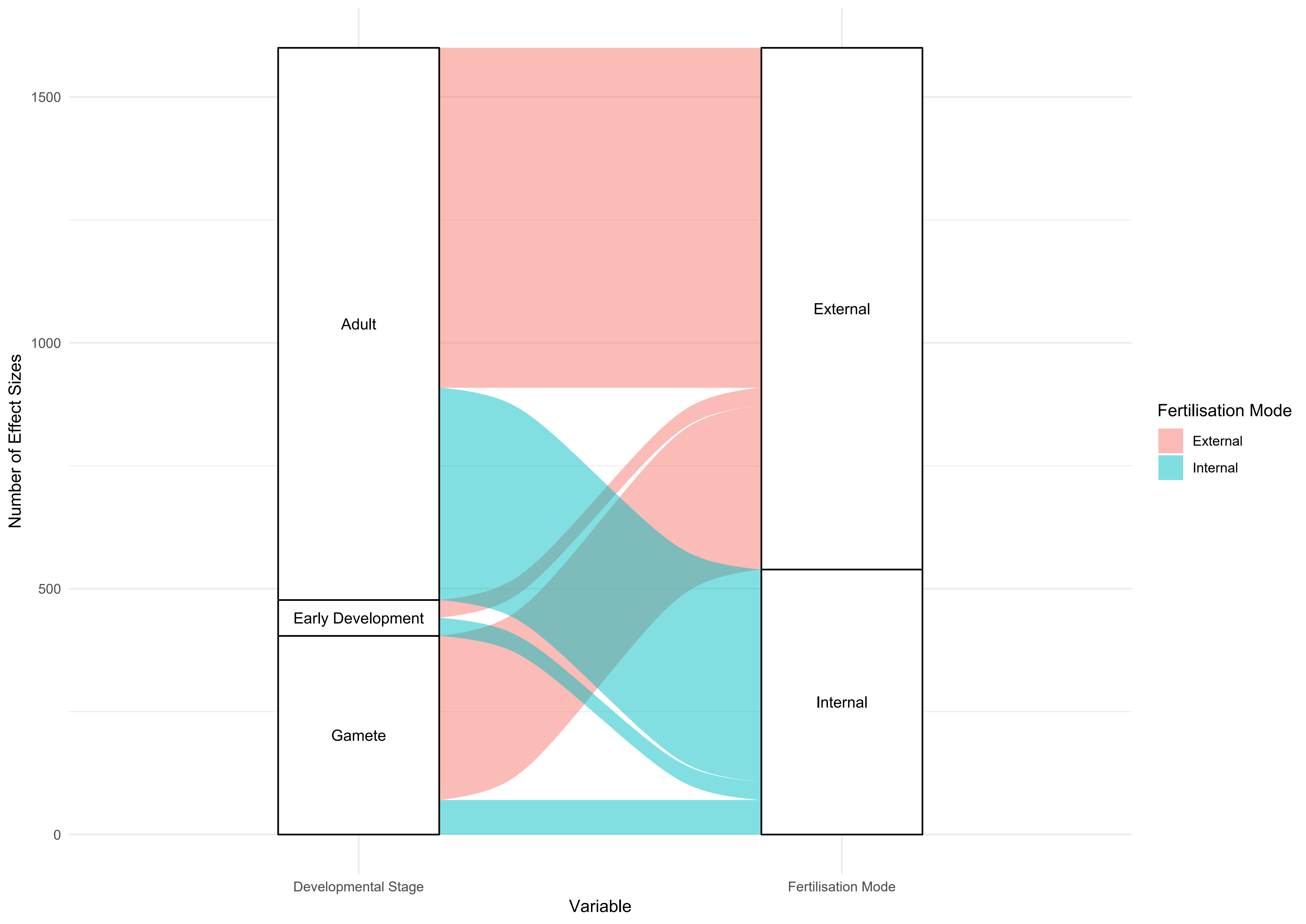

Number of Effect Sizes
1500
1000
500
0
Adult
Early Development
Gamete
External
Internal
Developmental Stage
Fertilisation Mode
Variable
Fertilisation Mode
External
Internal

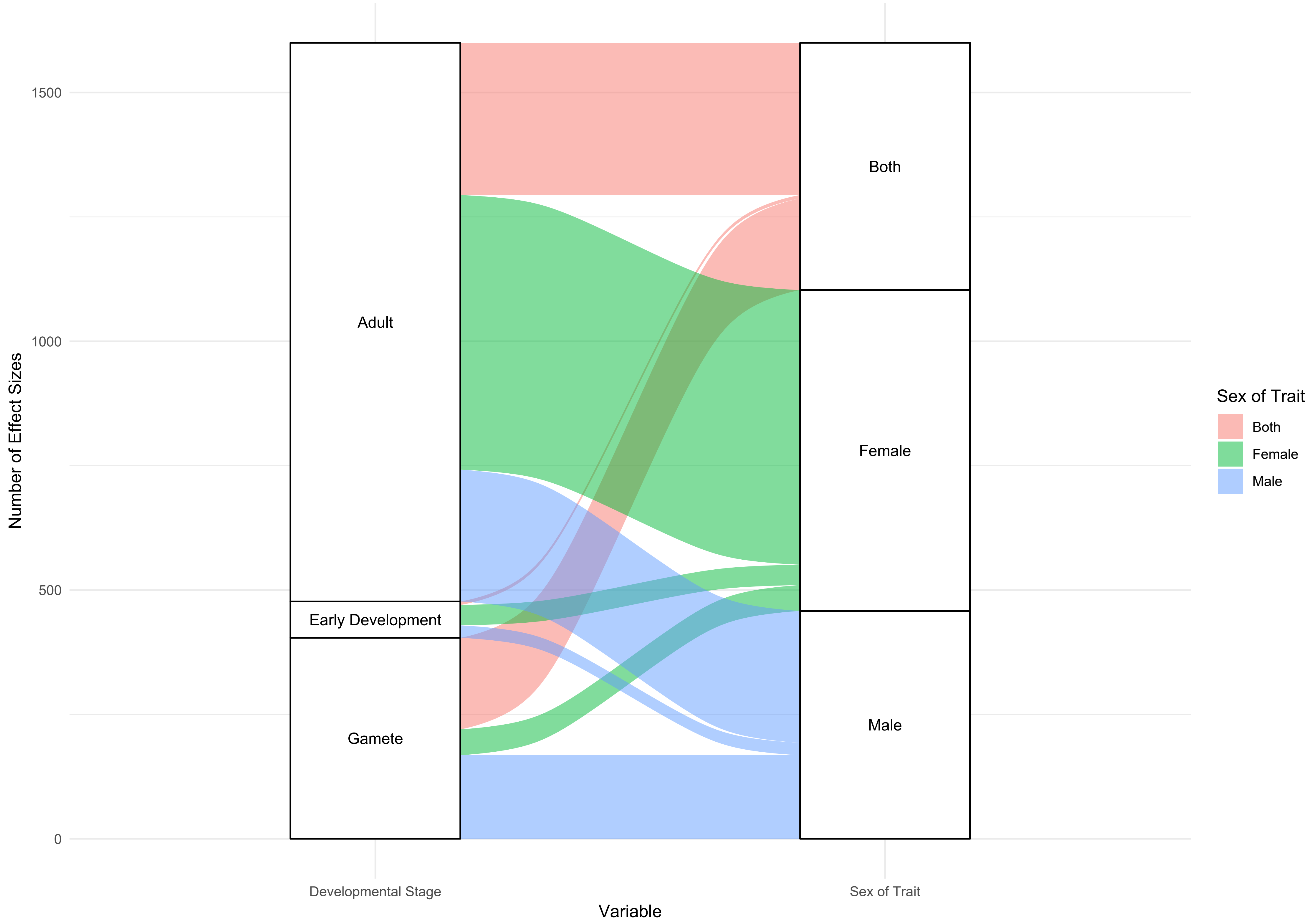
Number of Effect Sizes
1500
1000
500
0
Adult
Early Development
Gamete
Both
Female
Male
Developmental Stage
Sex of Trait
Variable
Sex of Trait
Both
Female
Male

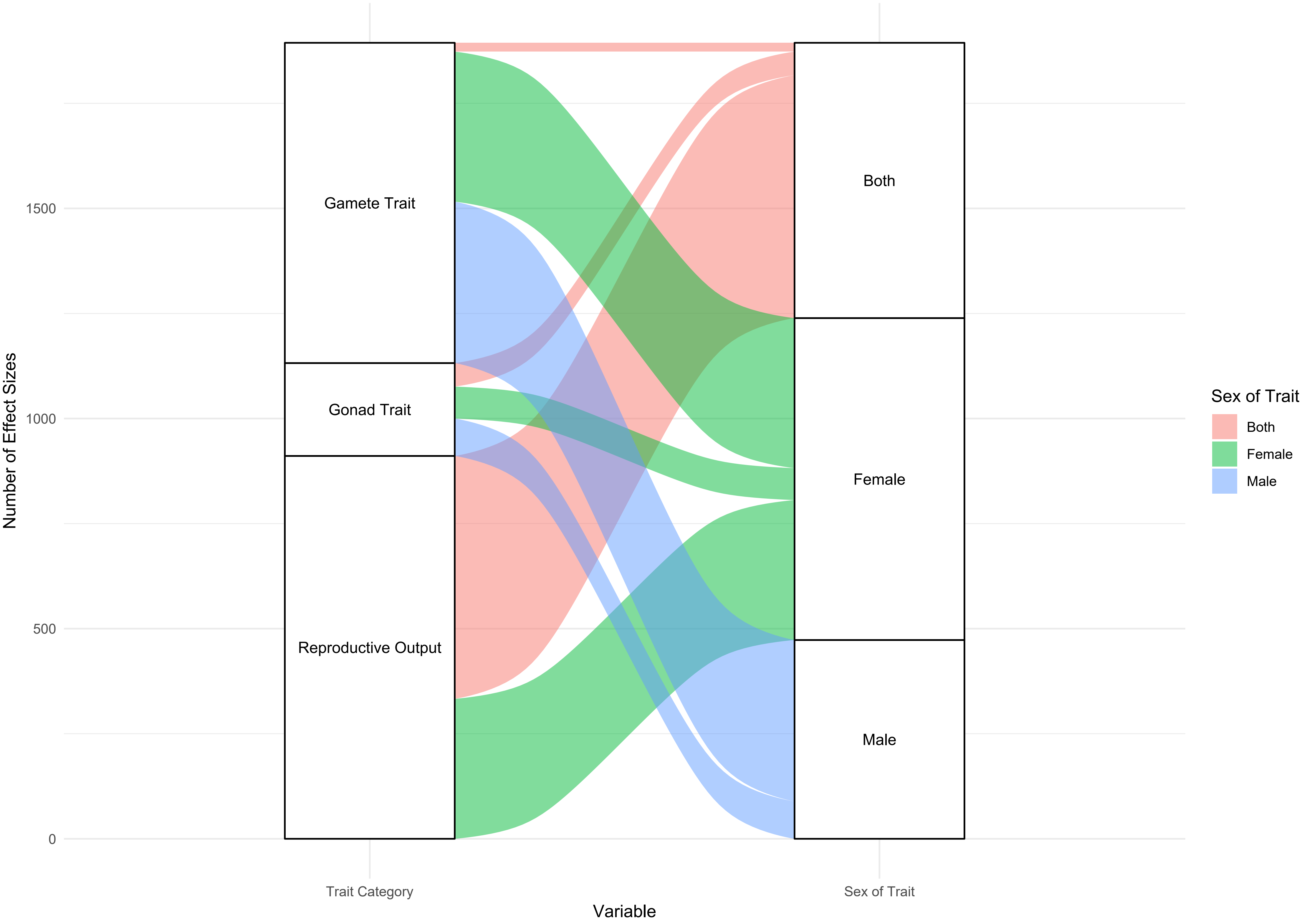

Number of Effect Sizes
0
500
1000
1500
Gamete Trait
Gonad Trait
Reproductive Output
Both
Female
Male
Trait Category
Sex of Trait
Variable
Sex of Trait
Both
Female
Male

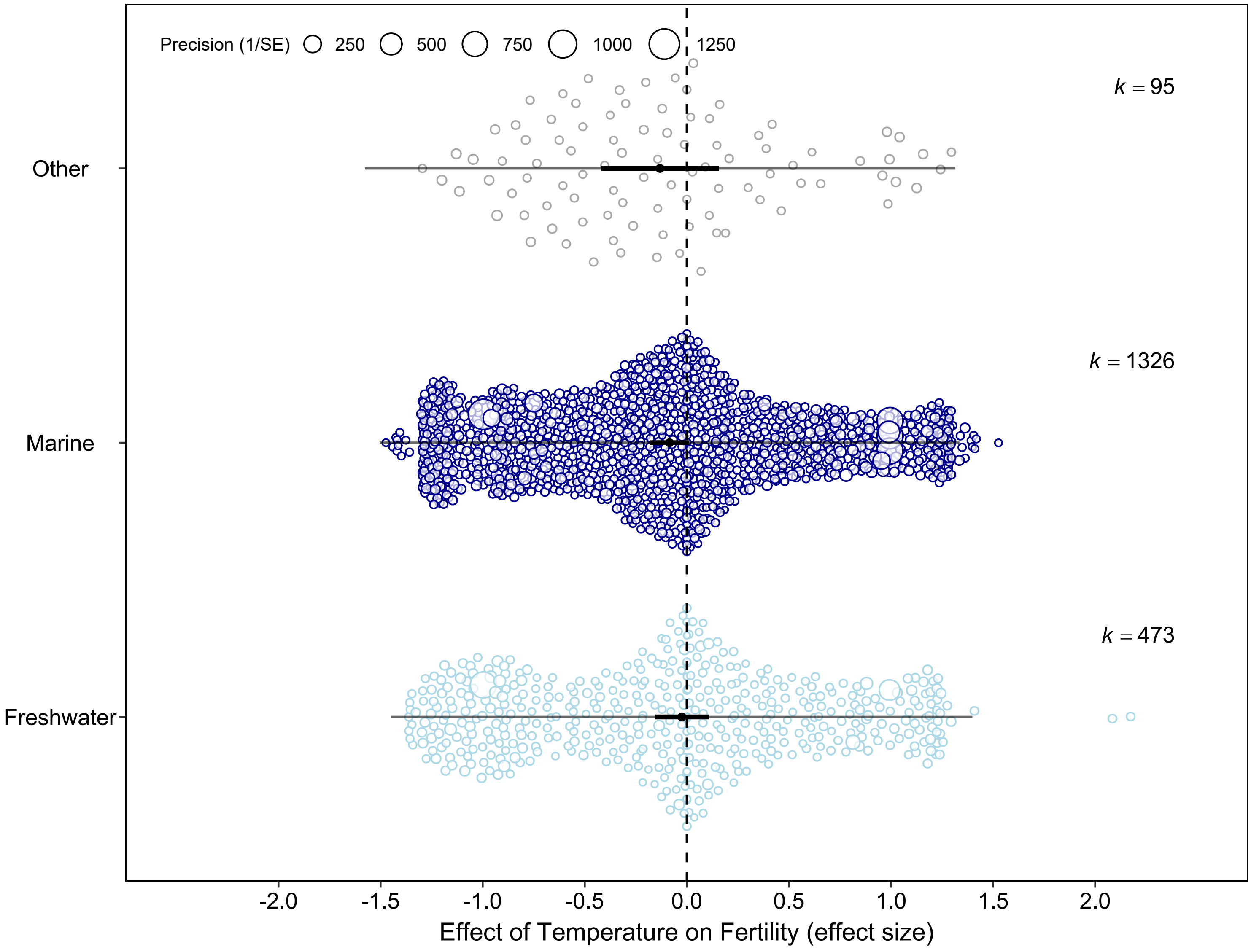
Precision (1/SE)
250
500
750
1000
1250
k = 95
k = 1326
k = 473
Other
Marine
Freshwater
-2.0
-1.5
-1.0
-0.5
0.0
0.5
1.0
1.5
2.0
Effect of Temperature on Fertility (effect size)

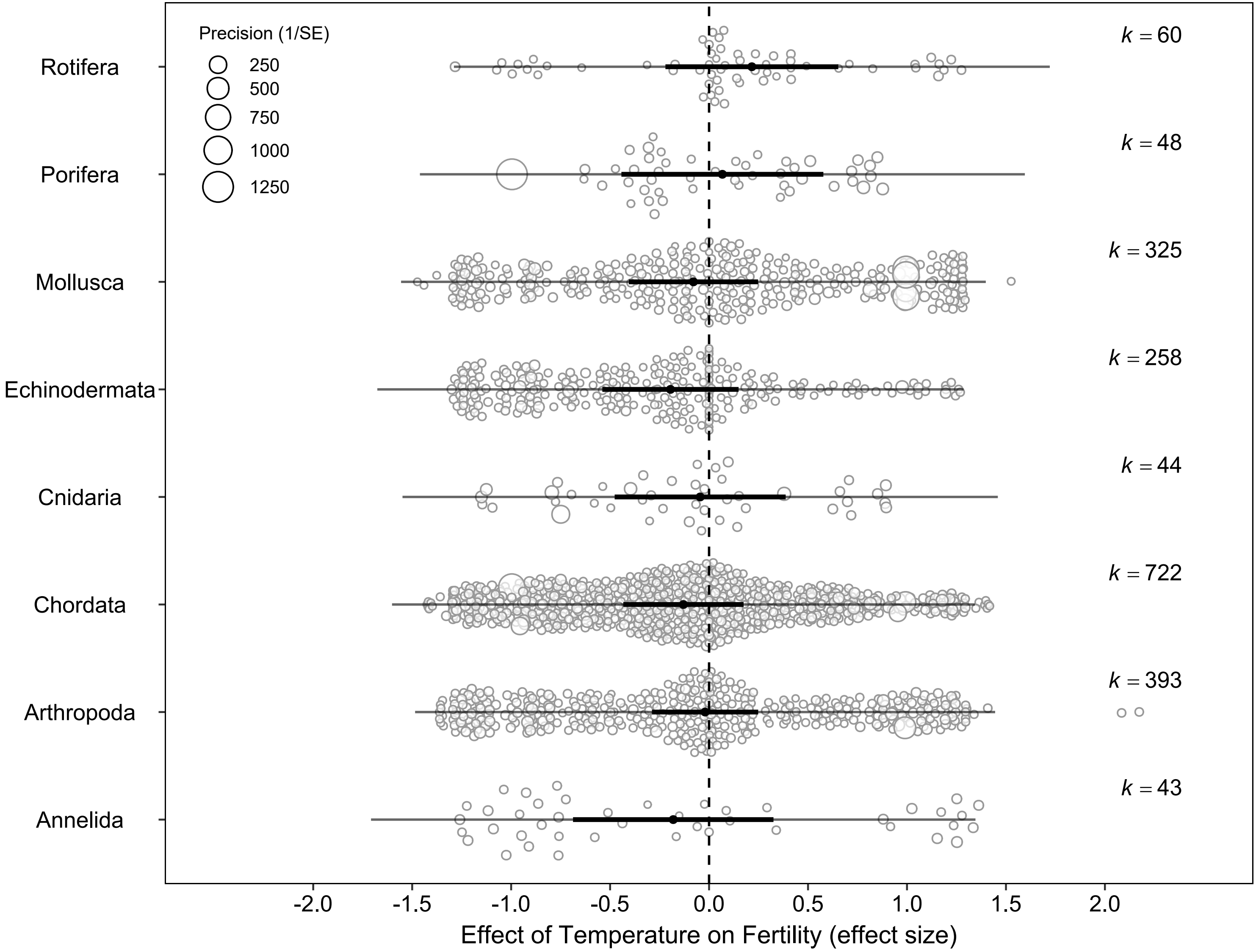

Precision (1/SE)
250
500
750
1000
1250
Rotifera
Porifera
Mollusca
Echinodermata
Cnidaria
Chordata
Arthropoda
Annelida
k = 60
k = 48
k = 325
k = 258
k = 44
k = 722
k = 393
k = 43
-2.0
-1.5
-1.0
-0.5
0.0
0.5
1.0
1.5
2.0
Effect of Temperature on Fertility (effect size)

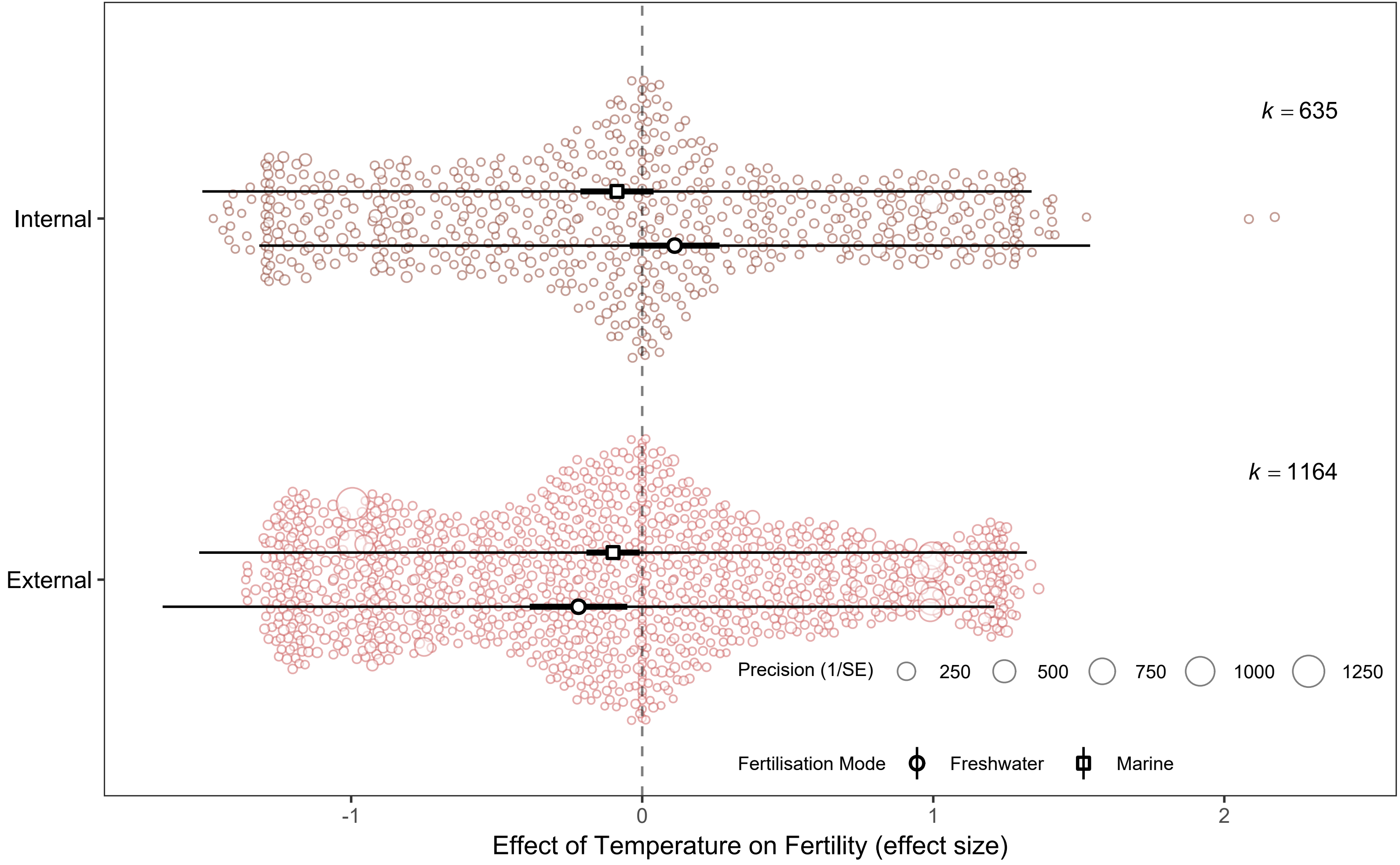

k = 635
k = 1164
Internal
External
Precision (1/SE)
250
500
750
1000
1250
Fertilisation Mode
Freshwater
Marine
-1
0
1
2
Effect of Temperature on Fertility (effect size)

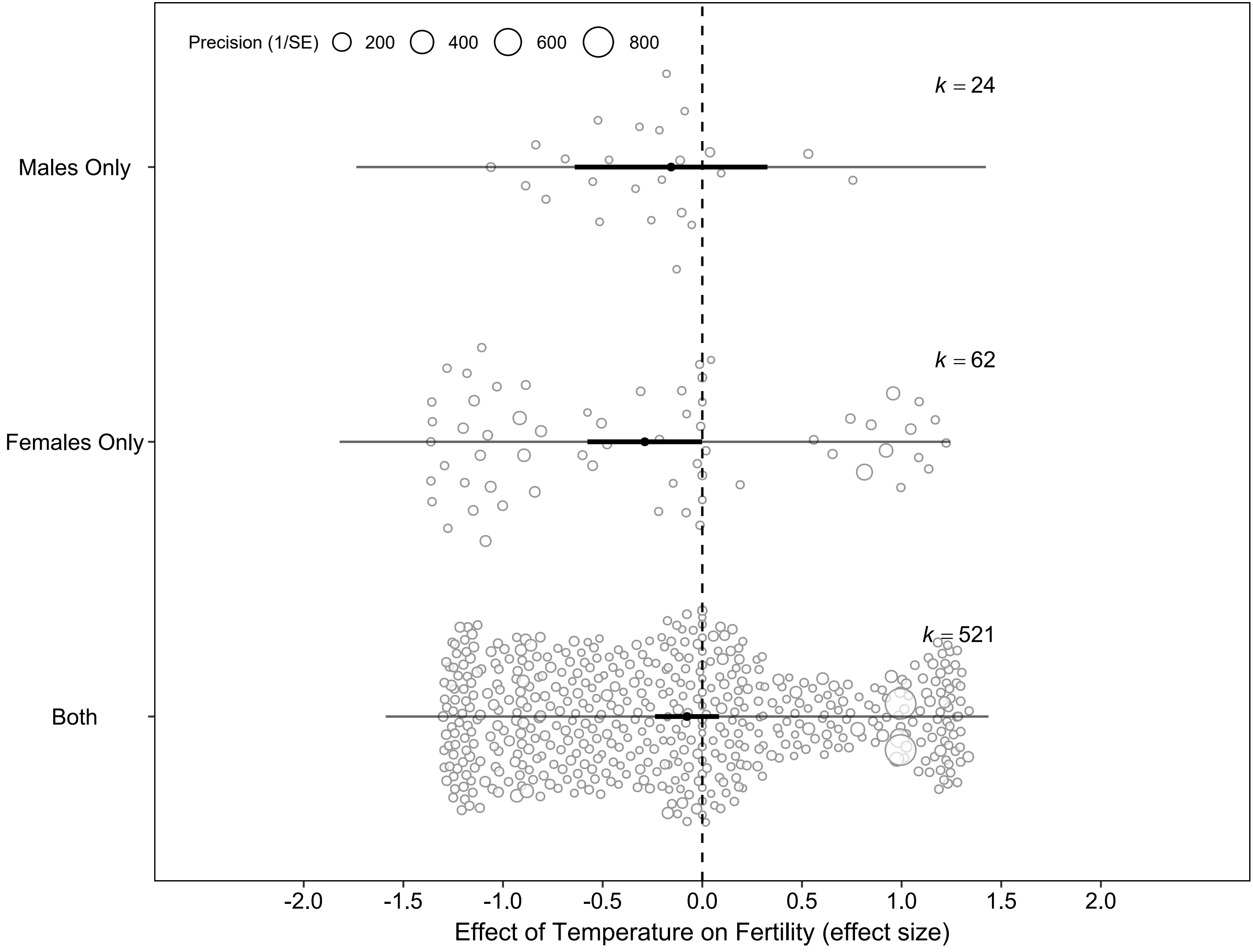
Precision (1/SE)
200
400
600
800
k = 24
k = 62
k = 521
Males Only
Females Only
Both
-2.0
-1.5
-1.0
-0.5
0.0
0.5
1.0
1.5
2.0
Effect of Temperature on Fertility (effect size)

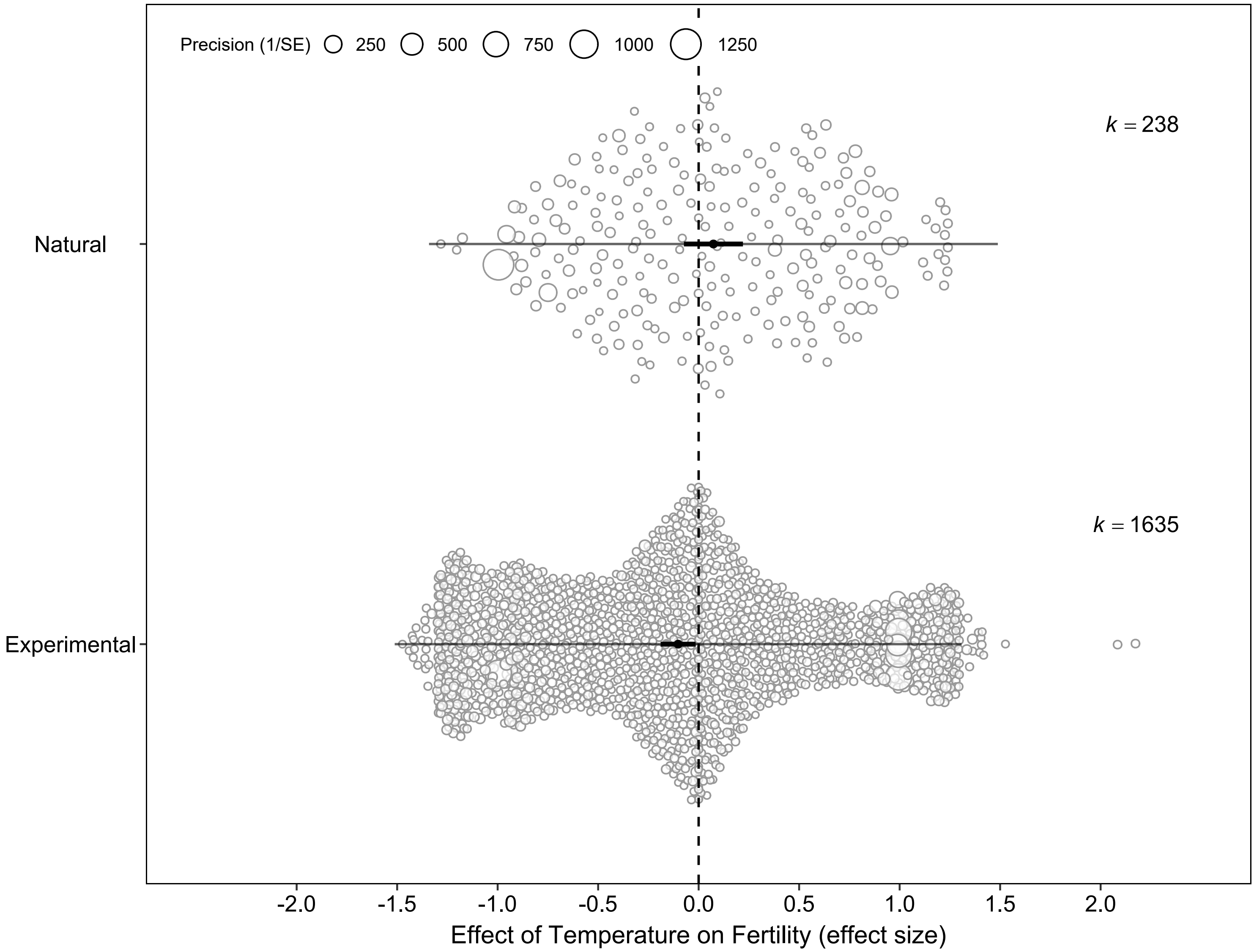
Precision (1/SE) 250 500 750 1000 1250
$k = 238$
$k = 1635$
Natural
Experimental
-2.0 -1.5 -1.0 -0.5 0.0 0.5 1.0 1.5 2.0
Effect of Temperature on Fertility (effect size)

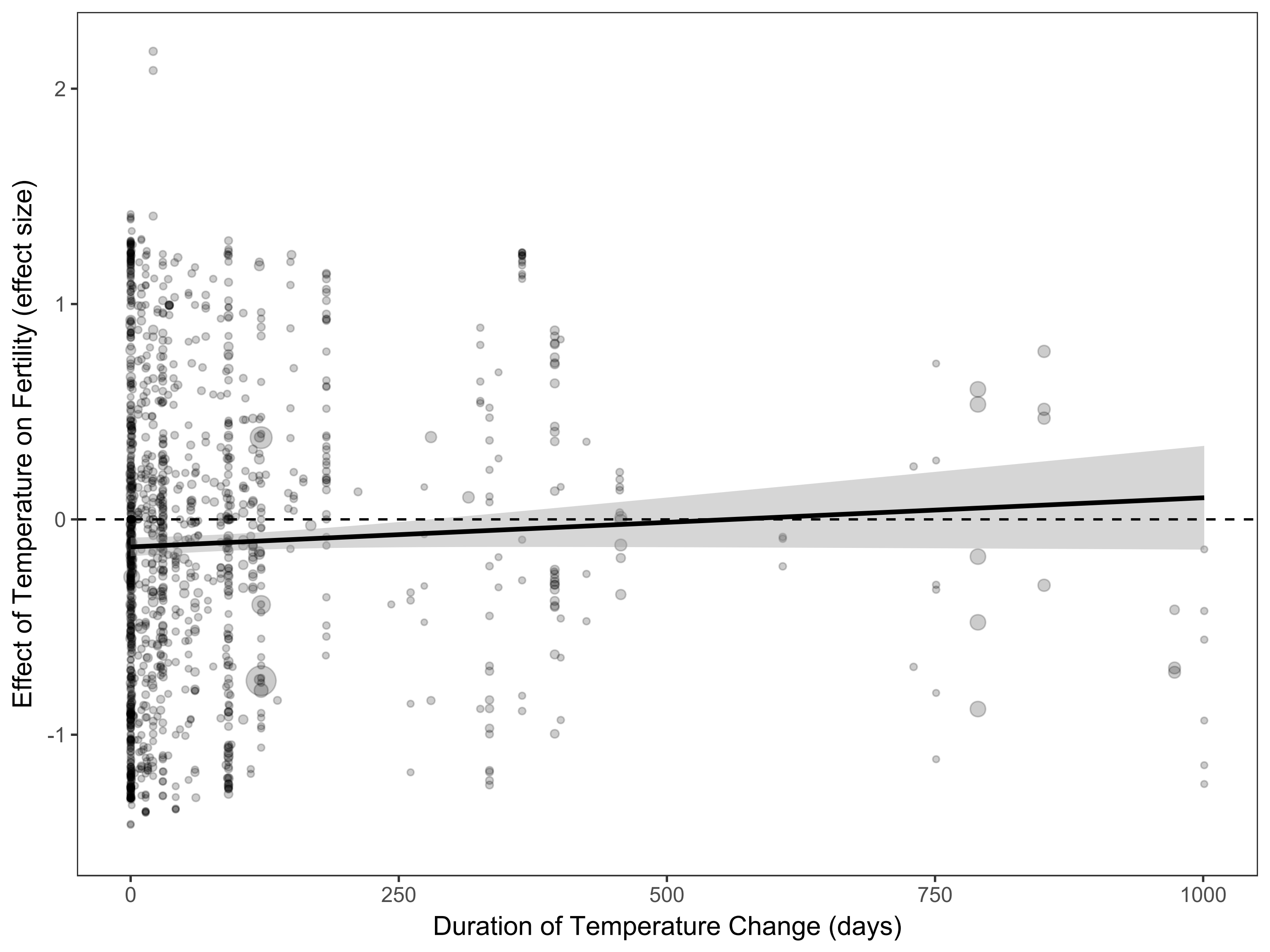

Effect of Temperature on Fertility (effect size)
2
1
0
-1
0
250
500
750
1000
Duration of Temperature Change (days)

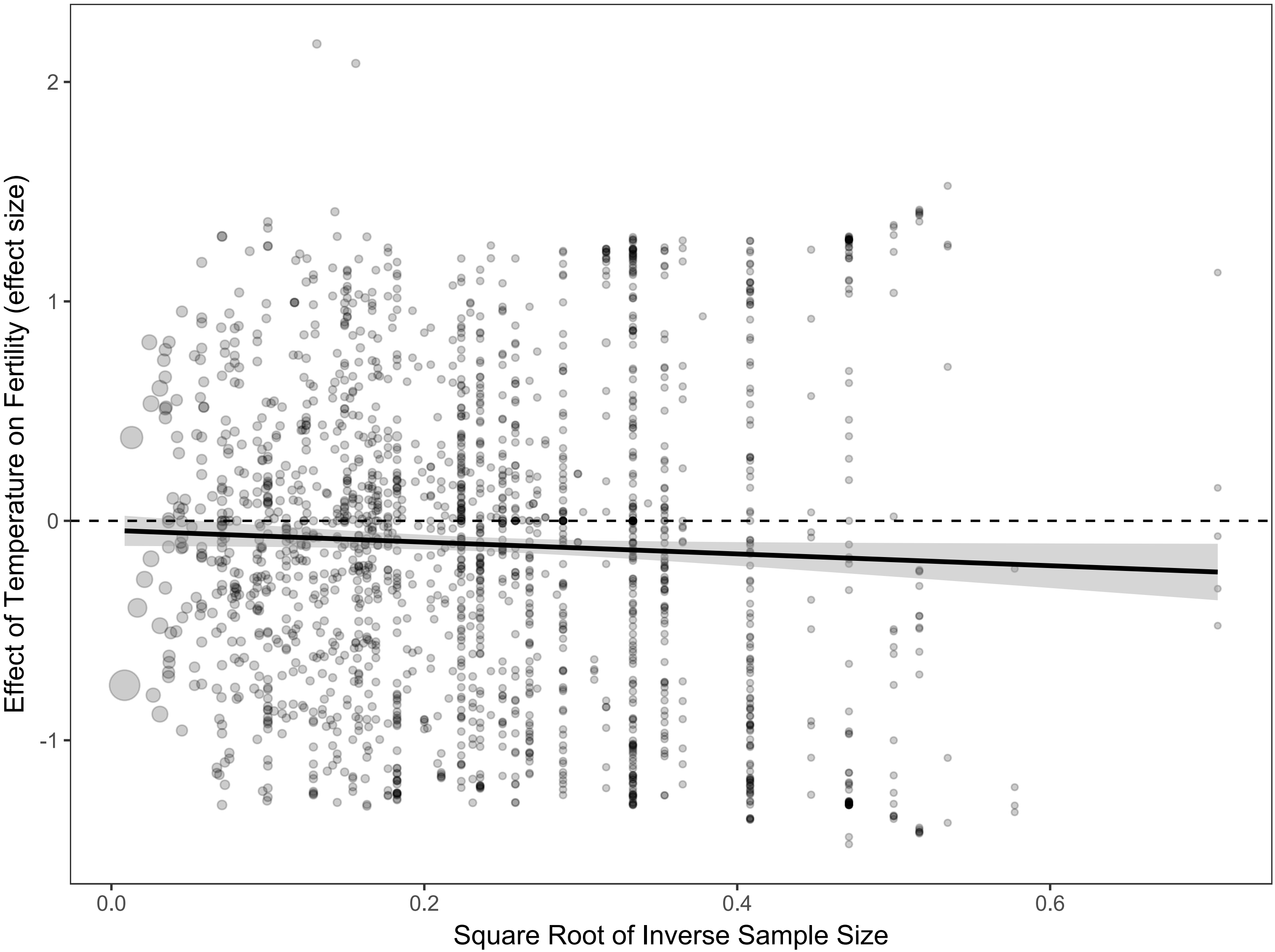

2
1
0
-1
0.0
0.2
0.4
0.6
Effect of Temperature on Fertility (effect size)
Square Root of Inverse Sample Size

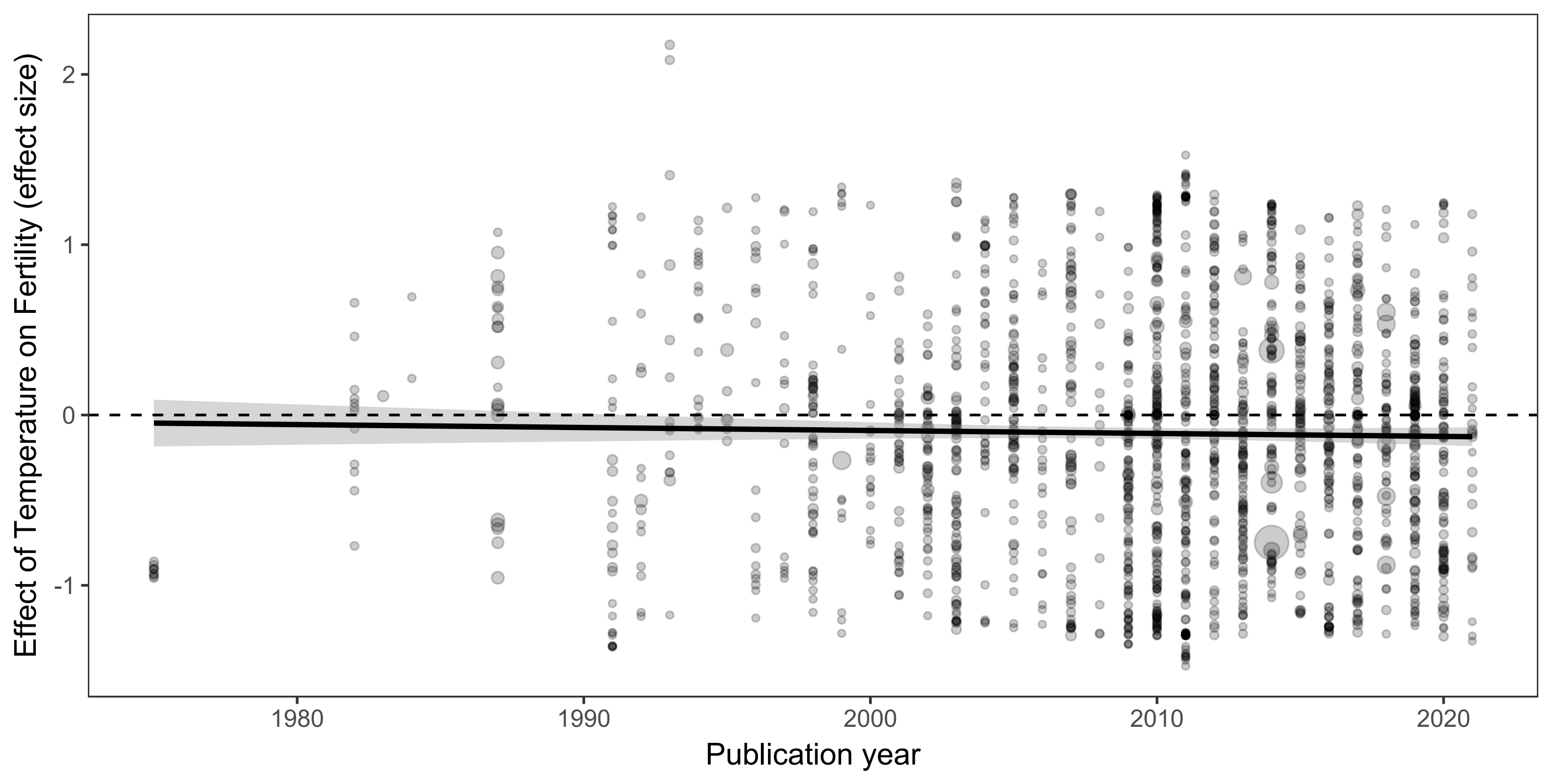

Effect of Temperature on Fertility (effect size)
2
1
0
-1
1980
1990
2000
2010
2020
Publication year
